# Molecular Characterization, Gene Evolution, and Expression Analysis of the Fructose-1, 6-bisphosphate Aldolase (FBA) Gene Family in Wheat (*Triticum aestivum* L.)

**DOI:** 10.3389/fpls.2017.01030

**Published:** 2017-06-14

**Authors:** Geng-Yin Lv, Xiao-Guang Guo, Li-Ping Xie, Chang-Gen Xie, Xiao-Hong Zhang, Yuan Yang, Lei Xiao, Yu-Ying Tang, Xing-Lai Pan, Ai-Guang Guo, Hong Xu

**Affiliations:** ^1^College of Life Sciences, Northwest A & F UniversityYangling, China; ^2^State Key Laboratory of Crop Stress Biology for Arid AreasYangling, China; ^3^Department of Food Crop Science, Cotton Research Institute, Shanxi Academy of Agricultural Sciences (CAAS)Yuncheng, China

**Keywords:** FBA, wheat, chromosomal localization, genomic structure, protein structure, expression analysis, light/dark treatment, abiotic stress responses

## Abstract

Fructose-1, 6-bisphosphate aldolase (FBA) is a key plant enzyme that is involved in glycolysis, gluconeogenesis, and the Calvin cycle. It plays significant roles in biotic and abiotic stress responses, as well as in regulating growth and development processes. In the present paper, 21 genes encoding TaFBA isoenzymes were identified, characterized, and categorized into three groups: class I chloroplast/plastid *FBA* (CpFBA), class I cytosol *FBA* (cFBA), and class II chloroplast/plastid *FBA*. By using a prediction online database and genomic PCR analysis of *Chinese Spring* nulli-tetrasomic lines, we have confirmed the chromosomal location of these genes in 12 chromosomes of four homologous groups. Sequence and genomic structure analysis revealed the high identity of the allelic *TaFBA* genes and the origin of different *TaFBA* genes. Numerous putative environment stimulus-responsive *cis*-elements have been identified in 1,500-bp regions of *TaFBA* gene promoters, of which the most abundant are the light-regulated elements (LREs). Phylogenetic reconstruction using the deduced protein sequence of 245 *FBA* genes indicated an independent evolutionary pathway for the class I and class II groups. Although, earlier studies have indicated that class II FBA only occurs in prokaryote and fungi, our results have demonstrated that a few class II CpFBAs exist in wheat and other closely related species. Class I TaFBA was predicted to be tetramers and class II to be dimers. Gene expression analysis based on microarray and transcriptome databases suggested the distinct role of TaFBAs in different tissues and developmental stages. The *TaFBA 4–9* genes were highly expressed in leaves and might play important roles in wheat development. The differential expression patterns of the *TaFBA* genes in light/dark and a few abiotic stress conditions were also analyzed. The results suggested that LRE cis-elements of *TaFBA* gene promoters were not directly related to light responses. Most *TaFBA* genes had higher expression levels in the roots than in the shoots when under various stresses. Class I cytosol *TaFBA* genes, particularly *TaFBA10/12/18* and *TaFBA13/16*, and three class II *TaFBA* genes are involved in responses to various abiotic stresses. Class I CpFBA genes in wheat are apparently sensitive to different stress conditions.

## Introduction

Fructose-1,6-bisphosphate aldolase (EC 4.1.2.13, FBA, or FBPA), also known as aldolase (ALD), is a key metabolic enzyme that catalyzes the reversible aldol cleavage of fructose-1,6- bisphosphate (FBP) into dihydroxyacetone phosphate (DHAP) and glyceraldehyde-3-phosphate (GAP), either in glycolysis or gluconeogenesis and in the Calvin-Benson cycle (Rutter, 1964; Ronimus and Morgan, 2003; Berg et al., 2010). These reactions are involved in carbon fixation and sucrose metabolism and are present in the chloroplast stroma and in the cytosol of green plants (Anderson et al., 2005).

FBAs could be classified into two groups based on different catalytic mechanisms and independent occurrences in evolution (Rutter, 1964; Flechner et al., 1999). Class I FBAs are not inhibited by ethylene diamine tetraacetic acid (EDTA) or affected by potassium ions, and occur in some bacteria, archaea, higher plants, and animals (Penhoet et al., 1967; Tolan et al., 1987). Class II FBAs are inhibited by EDTA (Gross and Al, 1999) and are found in *Giardia lamblia*, most bacteria, fungi, and yeast (Marsh and Lebherz, 1992; Henze et al., 1998). Some non-green algae (e.g., *Cyanophora paradoxa*), which is a phylogenetically diverse group, have either class I or class II aldolases or both (Gross et al., 1994; Antia, 2007). Class I FBAs are commonly homotetramers (Perham, 1990; Lorentzen et al., 2004); these activate their donor substrate by forming an intermediate Schiff base with the ÃĆÂęÅ-amino group of a conserved lysine residue (Lebherz and Rutter, 1969; Plaumann et al., 1997). Class II FBAs are dimers, which may use divalent metal cations (mostly Zn^2+^, Fe^2+^, or Co^2+^) to activate their donor homologs, and they belong to the family of (β/α)8 TIM barrel enzymes (Lorentzen et al., 2003; Du et al., 2011). Class II enzymes are divided into subgroups “A” and “B,” depending on their amino acid sequences (Sánchez et al., 2002). Type A aldolases are zinc-dependent and participate in glycolysis and gluconeogenesis, whereas type B aldolases have different divalent metal cofactors and diverse metabolic roles and substrate specificities (Sauvé and Sygusch, 2001; Labbé et al., 2011).

In higher plants, both the cytosolic FBA (cFBA) and chloroplast/plastid FBA (CpFBA) belong to the class I-type of isoenzymes (Anderson and Advani, 1970; Krüger and Schnarrenberger, 1983; Lebherz et al., 1984). These proteins play a vital role in carbohydrate metabolism (Anderson et al., 1995) and in signal transduction (Cho and Yoo, 2011). CpFBA are bifunctional for the formation of fructose-1, 6-bisphosphate (FBP) and sedoheptulose-1,7- bisphosphate (SuBP) in the Calvin cycle (Flechner et al., 1999), as well as function as a sedoheptulose/fructose-bisphosphate aldolase (SFBA) (Peltier et al., 2006). FBP aldolase activity is also part of the glycolytic and gluconeogenic reaction sequence in the cytoplasm, whereas the function of SuBP aldolase in plants is limited to the Calvin cycle that occurs in plastids (Flechner et al., 1999).

Different members of the *FBA* gene family have been identified and characterized in various plant species, including maize (Dennis et al., 1988), *Arabidopsis* (Lu et al., 2012), rice (Kagaya et al., 1995), spinach (Pelzer-Reith et al., 1993), tobacco (Yamada et al., 2000), *Sesuvium portulacastrum* (Fan et al., 2009), and tomato (Cai et al., 2016). *FBA* genes have been shown to be involved in various important physiological and biochemical processes, e.g., plant development (Zhang et al., 2014), signal transduction (Oelze et al., 2014), regulation of secondary metabolism (Zeng et al., 2014), plant defense and response to biotic (Mohapatra and Mittra, 2016), and abiotic stresses, including salt (Lu et al., 2012), cadmium (Sarry et al., 2006), drought (Khanna et al., 2014), chilling (Purev et al., 2008), and heat (Michelis and Gepstein, 2000), and post-translational modification (Mininno et al., 2012). Furthermore, the presence of *FBAs* in the nucleus implies that aldose isoenzymes could bind to DNA and directly regulate gene expression (Páez-Valencia et al., 2008).

Common wheat (*Triticum aestivum* L.) is a hexaploid species (6× = 2*n* = 42, AABBDD) that originated from two inter-specific hybridizations that are estimated to have taken place ~0.5 million and 10,000 years ago (Dubcovsky and Dvorak, 2007). Its predicted most closely related extant diploid species (2*n* = 14) include *Triticum monococcum* or *T. urartu* (donors for A genome), *Aegilops speltoides* (S genome related to the B genome), and *A.tauschii* (D genome). The hexaploid genome of wheat has three homeologous sets of seven pairs of chromosomes (1A to 7A, 1B to 7B, and 1D to 7D), each carrying highly similar gene copies (Choulet et al., 2014).

Despite extensive studies on the *FBA* genes in various plant species, our understanding of its function in wheat remains unclear. A previous study has revealed that CpFBA accounts for 86% of the total aldolase activity in wheat leaves (Schnarrenberger and Krüger, 1986), and wheat *FBA* genes could be activated by various abiotic stresses (Xue et al., 2008). FBA has also been linked to male sterility (Li et al., 2012). Consequently, in-depth research investigations on the *FBA* genes of wheat may provide insights on methods in modifying photosynthesis in increase crop yield.

In the present study, 21 members of the *FBA* gene family in wheat were identified and cloned. We investigated the molecular characteristics, chromosomal location, gene evolution, protein structures, and expression patterns of these genes. Genome-wide identification, phylogenetic structural and functional analysis revealed the role of the *FBA* gene family in wheat, which subsequently may be applied to crop production and improvement.

## Materials and methods

### Identification of FBA genes

A total of 21 *FBA* genes from *Triticum aestivum* L. (*TaFBA*) were identified by BLAST analysis against the URGI (https://urgi.versailles.inra.fr) and Ensembl Plants (http://plants.ensembl.org) databases using the gene sequences of previously annotated *FBA* genes in wheat (GenBank No. FJ403591.1, AJ420778.1, FJ625793.3, KR139996.1, KR139997.1) and *Arabidopsis* (GenBank No: AT2G21330, At4G38970, AT2G01140, AT5G03690, AT4G26530, AT2G36460, AT4G26520, AT3G52930, AT1G18270) with E-value < 1e^−10^. Using these 21 *TaFBA* genes as queries, another 31 *FBA* genes from *T. monococcum* (6 genes), *T. urartu* (7 genes), *A. speltoides* (5 genes), *A. sharonensis* (6 genes), and *A. tauschii* (7 genes) were further identified from the URGI database. Using the genome sequences of these *FBA* genes downloaded from the URGI database as queries, the FBA cDNA sequences of *T. aestivum, T. urartu, A. sharonensis*, and *A. tauschii* were obtained from the WheatExp database (http://wheat.pw.usda.gov/WheatExp/) and NCBI's TSA and SRA databases. The FBA cDNA sequences of *T. monococcum* and *A. speltoides* were predicted by homology analysis.

### Chromosomal localization of wheat *FBA* gene family

We used URGI, Ensembl Plants databases, wheat_2014_90K SNP gene chip, and *Chinese Spring*_Deletion gene chip to predict the chromosomal localization of the *TaFBA* genes. To further confirm our results, gDNA PCR amplification of 21 nulli-tetrasomic lines (NA-TD, NB-TD, and ND-TB) of *T. aestivum L*.var. *Chinese Spring* was performed. The gene-specific primers used in PCR analysis are listed in Table S2. Nulli-tetrasomic *Chinese Spring* genotypes contain four copies of one chromosome pair (tetrasomic) to compensate for the lack of a homoeologous chromosome pair (nullisomic) (Sears et al., 1966). For instance, Nulli1A-Tetra1D (N1AT1D) lacks chromosome 1A (nullisomic-1A), but contains two chromosome pairs of chromosome 1D (tetrasomic-1D), resulting in a line containing no copies of chromosome 1A, two doses of chromosome 1B, and four doses of chromosome 1D. PCR analysis of genomic DNA from nulli-tetrasomic lines allows one to determine which chromosome gene is located on based on failure to amplify specific nullisomic lines (Ohnishi et al., 2008). PCR amplification was performed as follows: 94°C for 5 min; 94°C for 30 s, annealing at 60°C for 30 s, and extension at 72°C for 1 min, 35 cycles; and 72°C for 10 min for the final extension. PCR reactions were performed using the proofreading enzymes LA Taq or PrimeSTAR® HS DNA Polymerase (TaKaRa, Japan) depending on the application.

### DNA cloning and sequence alignment

21 *TaFBA* genes (including 1.5-kb promoter regions) were cloned by genomic PCR of *T. aestivum* L. var. *Chinese Spring*. PCR protocols were the same as that used in chromosomal localization described in 2.2. The promoter sequences of two *TaFBA* genes (*TaFBA6* and *TaFBA12*) were obtained by high-efficiency thermal asymmetric interlaced PCR (hiTAIL-PCR) (Liu and Chen, 2007) due to the incomplete sequencing of the wheat genome. The primers used in this study are listed in Table S2. The PCR products were purified using an AxyPrep DNA gel extraction kit (Axygen, USA) and ligated into the pMD19-T vector (TaKaRa, Japan). The recombinant plasmid DNA clones were sequenced by Invitrogen (USA). The generated *TaFBA* gene sequences were then submitted to GenBank of NCBI (Accession No. were listed in Table 1).

**Table 1:** The molecular characteristics of *TaFBA* genes and the prediction of chromosomal and subcellular location.

**Genes**	**Chr**.	**GenBank accession no**.	**Subfamily**	**gDNA Length(bp)**	**Protein Length (aa)**	**MW (kDa)**	**pI**	**GRAVY**	**Subcellular location**
*TaFBA1*	3AS	KY930446	I	3,662	387	41.69	7.61	−0.163	Chlo
*TaFBA2*	3BS^*^	KY930447	I	3,720	385	41.36	8.21	−0.168	Chlo
*TaFBA3*	3DS	KY930448	I	3,975	387	41.66	8.21	−0.156	Chlo
*TaFBA4*	4AL	KY930449	I	3,180	388	41.97	5.94	−0.148	Chlo
*TaFBA5*	4BS	KY930450	I	3,058	388	41.87	6.78	−0.121	Chlo
*TaFBA6*	4DS	KY930451	I	3,066	388	41.97	5.94	−0.148	Chlo
*TaFBA7*	5AS	KY930452	I	1,810	385	41.64	6.08	−0.165	Chlo
*TaFBA8*	5BS	KY930453	I	1,981	385	41.62	6.08	−0.165	Chlo
*TaFBA9*	5DS	KY930454	I	1,807	385	41.64	6.08	−0.165	chlo
*TaFBA10*	3AL	KY930455	I	2,539	358	38.81	6.85	−0.229	Cyto
*TaFBA11*	3AL	KY930456	I	2,369	358	38.91	6.4	−0.25	Cyto
*TaFBA12*	3BL^*^	KY930457	I	2,161	358	38.81	6.85	−0.223	Cyto
*TaFBA13*	3BL^*^	KY930458	I	3,378	358	38.90	6.85	−0.241	Cyto
*TaFBA14*	3DL	KY930459	I	2,463	358	38.80	6.39	−0.223	Cyto
*TaFBA15*	3DL	KY930460	I	2,629	358	38.78	6.85	−0.23	Cyto
*TaFBA16*	3DL	KY930461	I	2,466	358	38.88	7.51	−0.256	Cyto
*TaFBA17*	5AS	KY930462	I	996	244	25.93	9.37	−0.149	Cyto
*TaFBA18*	5BS	KY930463	I	7,970	519	55.39	8.85	−0.016	Cyto
*TaFBA19*	7AS	KY930464	II	17,081	1,383	148.61	5.73	0.089	Chlo/plastid
TaFBA20	7BS	KY930465	II	21,180	1,383	148.28	5.61	0.105	Chlo/plastid
TaFBA21	7DS	KY930466	II	14,360	1,383	148.36	5.83	0.098	Chlo/plastid

*GRAVY, the grand average of hydropaths. gDNA length is the length of sequence from the 5′ UTR to 3′ UTR of the TaFBA genomic DNA sequences*.

* *Means the chromosomal location predicted on GrainGenes-SQL databases*.

Multiple sequences alignment of 21 *TaFBA* genes and 31 *FBA* genes from wheat relatives were performed by MUSCLE (http://www.ebi.ac.uk/Tools/msa/muscle/). A rooted UPGMA phylogenetic tree was constructed with MEGA 6 under default parameters and bootstrapping with 1,000 replicates (Tamura et al., 2011). Genome sequences, cDNA sequences and protein primary sequences of FBAs were aligned by DNAMAN 6.0 software with pairwise method and were clustered by MeV 4.9 using the average linkage hierarchical clustering (HCL) method.

### Annotation of *TaFBA* gene structures and cis-regulatory elements in the promoter region

The exon-intron structure of *TaFBA* genes was determined by aligning the cDNA sequences to the corresponding genomic sequences using Gene Structure Display Server (GSDS, http://gsds.cbi.pku.edu.cn). The transposable elements of *TaFBA* genes were predicted on TREP (http://botserv2.uzh.ch/kelldata/trep-db/blast/blastTREP.html) and LTR_FINDER (http://tlife.fudan.edu.cn/ltr_finder/). Putative cis-acting regulatory DNA elements in the promoter sequences (1.5-kb upstream of the 5′-UTR) of *TaFBA* genes were annotated with PlantCARE (http://bioinformatics.psb.ugent.be/webtools/plantcare/html/), and displayed in Argo Genome Browser v1.0.31 (http://www.broadinstitute.org/annotation/argo/). The exon-intron structure of the *TaFBA* genes was determined by aligning the cDNA sequences to the corresponding genomic sequences using the Gene Structure Display Server (GSDS, http://gsds.cbi.pku.edu.cn). The transposable elements of the *TaFBA* genes were predicted by using TREP (http://botserv2.uzh.ch/kelldata/trep-db/blast/blastTREP.html) and LTR_FINDER (http://tlife.fudan.edu.cn/ltr_finder/). Putative cis-acting regulatory DNA elements in the promoter region (1.5-kb upstream of the 5'-UTR) of the *TaFBA* genes were annotated with PlantCARE (http://bioinformatics.psb.ugent.be/webtools/plantcare/html/) and displayed in the Argo Genome Browser v1.0.31 (http://www.broadinstitute.org/annotation/argo/).

### The primary structure and three-dimensional (3D) structure prediction of the FBA proteins

The physicochemical characteristics of the protein sequence of each FBA such as the number of amino acids (aa), molecular weight (MW), isoelectric point (pI), and grand average of hydropathy (GRAVY) value were calculated with the ProtParam tool in ExPASy (http://web.expasy.org/protparam/). The subcellular location of each FBA was predicted by using WoLF PSORT (Horton et al., 2007). Functional domains and conserved motifs were annotated with the Pfam database (Finn et al., 2016) and the MEME website (http://meme-suite.org/tools/meme).

A total of 245 FBA proteins of archaea, bacteria, and eukaryotes were identified by PHMMER searches on the Ensembl Plants database and key word searches of “fructose-1,6-bisphosphate aldolase” in the UniprotKB/Swiss-Prot databases. HMMER 3.0 was employed to perform an HMM search (Zhang and Wood, 2003), with the family-specific Glycolytic (PF00274) and F_bP_aldolase domain (PF01116).

Amino acid sequence alignment of the FBAs was performed by using the Clustal Omega method (Sievers et al., 2011), and a rooted maximum likelihood (ML) phylogenetic tree was constructed using MEGA 6. The phylogenetic tree files were edited by Evolview (http://www.evolgenius.info/evolview/). We used the I-TASSER program (Zhang, 2008) and Swiss-PdbViewer v4.1.0 (Schwede et al., 2003) to remodel and analyze the tertiary structure, and SWISS-MODEL to construct the quaternary structure of the TaFBAs.

### Gene expression profile analysis

To analyze the expression profiles of the *TaFBA* genes, the microarray data of the *TaFBA* genes were downloaded from PLEXdb (Dash et al., 2012) and the RNA-seq data from wheat transcriptome database WheatExp. Combined with the analysis using Genevestigator (Hruz et al., 2008), the expression profiles of different tissues and developmental stages of the *TaFBA* genes were generated by using OriginPro 9.1.

### Quantitative real-time PCR (qRT-PCR) analysis of the response of the *TaFBA* genes to different abiotic stresses

Seeds of *Chinese Spring* were sterilized with 75% alcohol for 30 s and 15% sodium hypochlorite for 5 min, rinsed 5 times, placed on moistened filter paper in Petri dishes, and germinated in the dark for 2 days. Then, the wheat seedlings were cultivated in Hoagland liquid culture medium at 23°C and 16 h light/8 h dark cycles. Treatment of the wheat seedlings was performed as described Zeng et al. (2016), with minor modifications. After 10 days of growth, salinity (200 mM NaCl), drought (15 % PEG 6000), ABA (50 μM ABA), heat (40°C), and cold (4°C) treatments were conducted. The shoots and roots of the seedlings were collected at 0, 1, 2, 6, 12, 24, and 48 h after treatment.

We investigated the influence of light on the expression of the *TaFBA* genes as described by Zhao et al. (2014). The etiolated wheat seedlings were cultured under continuous light, whereas the normal-cultured seedlings were placed in the dark after being exposure to light for 24 h. Wheat leaves were collected at 1, 2, 4, and 8 h after light or dark treatment, immediately frozen in liquid nitrogen, and kept at −80°C until RNA isolation. Next, total RNA were isolated by using the RNAiso plus reagent (TaKaRa, Japan) and treated with gDNA Eraser (TaKaRa, Japan) for 2 min at 42°C to degrade any residual genomic DNA. Then, first-strand cDNAs were synthesized from the total RNA by using a PrimeScript™ RT reagent kit (TaKaRa, Japan) according to the manufacturer's instructions. Finally, qRT- PCR was performed in optical 96-well plates (VIOX, UK) with a CFX96 Touch Real-Time PCR Detection System (BIO-RAD, USA) by using the SYBR Green method. The wheat ADP ribosylation factor (*ADP-RF*) gene was used as reference gene (Paolacci et al., 2009) to normalize the expression data. The thermal cycle protocol was set up as follows: 95°C for 30 s; 95°C for 5 s, and 60°C for 30 s for 40 cycles. The gene specific primers used for qRT-PCR were listed in Table S8. The quantitative analysis was accomplished with the 2^−ΔΔCT^ method. PCR was performed based on three technical replicates for each of the biological duplicates.

## Results

### Genome-wide identification and chromosomal localization of the FBA genes in wheat

To investigate the *TaFBA* gene families, BLAST analysis with *FBA* genes of wheat and *Arabidopsis thaliana* (GenBank Accession Nos. are shown in 2.1) was performed using the IWGSC database. A total of 21 *TaFBA* genes (named as *TaFBA1*~*21*) were identified and cloned (Data sheet DOC1, TXT2). The cDNA sequences (Data Sheet TXT1) were obtained from NCBI and the WheatExp Database.

We aligned the cDNA and predicted protein sequences of the *TaFBA* genes by using DNAMAN with the pairwise method (Table S1). Phylogenetic and sequence analyses revealed that the *TaFBA* genes could be classified into two subfamilies, classes I and II. There were 18 *TaFBA* genes in class I, with *TaFBA1*~*3, TaFBA4*~*6, TaFBA7*~*9*, and *TaFBA10*~*16* showing high identity (>90%) respectively. Meanwhile, three class II *TaFBA* genes (*TaFBA19*~*21*) were highly homologous and showed lower identity (< 30%) with those of class I.

The molecular characteristics of the *TaFBA* genes and their subcellular location are listed in Table 1. The predicted MW of class I TaFBA proteins ranged from 25.93 to 55.39 kDa, with TaFBA17 apparently being the smallest polypeptide, whereas class II TaFBAs were about 148 kDa, bigger than class I. WoLF PSORT was used to predict the subcellular location of 21 putative TaFBA proteins. Similar to *Arabidopsis*, rice, and tomato, 18 class I TaFBAs were located in two compartments: TaFBA1~9 were in the chloroplast/plastid and were thus designated as CpFBAs, whereas TaFBA10~18 were localized to the cytoplasm, and hereby named as cFBA. Three class II proteins TaFBA19~21 might also be located in the chloroplast/plastid, and thus described as CpFBAs.

### Chromosomal location of the *FBA* genes in wheat

Based on the 0.6× *Chinese Spring* genome draft sequence (Mayer et al., 2014), we predicted the chromosomal location of the *TaFBA* genes (Table 1). In addition, the *TaFBA* genes were queried on the mapped loci using EST-derived probes in GrainGenes-SQL databases. Therefore, the *TaFBA1, 2, 3, 4, 7, 8, 11, 19*, and *20* genes were mapped onto different gene loci through a wheat_2014_90K SNP gene chip (Figure S1A), and the *TaFBA10, 12, 14*, and *15* genes were mapped using a *Chinese Spring*_Deletion gene chip (Figure S1B).

**Figure 1:**
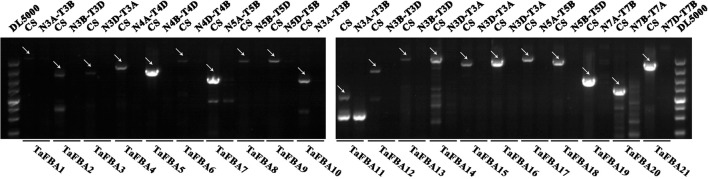
Genomic PCR amplification results of the TaFBA genes using Chinese spring nulli-tetrasomic lines. PCR amplification was performed using *Chinese Spring* (CS) and nulli-tetrasomic lines of *Chinese Spring* (N4-T4) genomic DNA. DL5000 is DNA marker (100, 250, 500, 750, 1,000, 1,500, 2,000, 3,000, 5,000 bp). The white arrows indicated the target PCR bands.

To confirm the chromosomal location and identify the primer specificity of *TaFBA* genes, 21 *Chinese Spring* nulli-tetrasomic lines were used to amplify genomic DNA (The gene -specific primers used are listed in Table S2). The PCR results (Figure 1) confirmed the location of each *TaFBA* gene copy, as indicated by the failed amplification in the specific nulli-tetrasomic line. These findings validated that the genes were located within the same locus as predicted in Table 1 and Figure S1.

A total of 21 *TaFBA* genes were localized to 12 chromosomes of 4 homoeologous groups: *TaFBA1*~*3* genes in the short arms and *TaFBA10*~*16* in the long arms of homoeologous group 3; the *TaFBA5/6* genes in the short arm, and *TaFBA4* in the long arm of homoeologous group 4; the *TaFBA7*~*9* and *TaFBA17/18* genes in the short arms of homoeologous group 5; and the *TaFBA19*~*21* genes located in the short arms of homoeologous group 7.

### Phylogenetic analysis of the FBA genes

**Figure 2:**
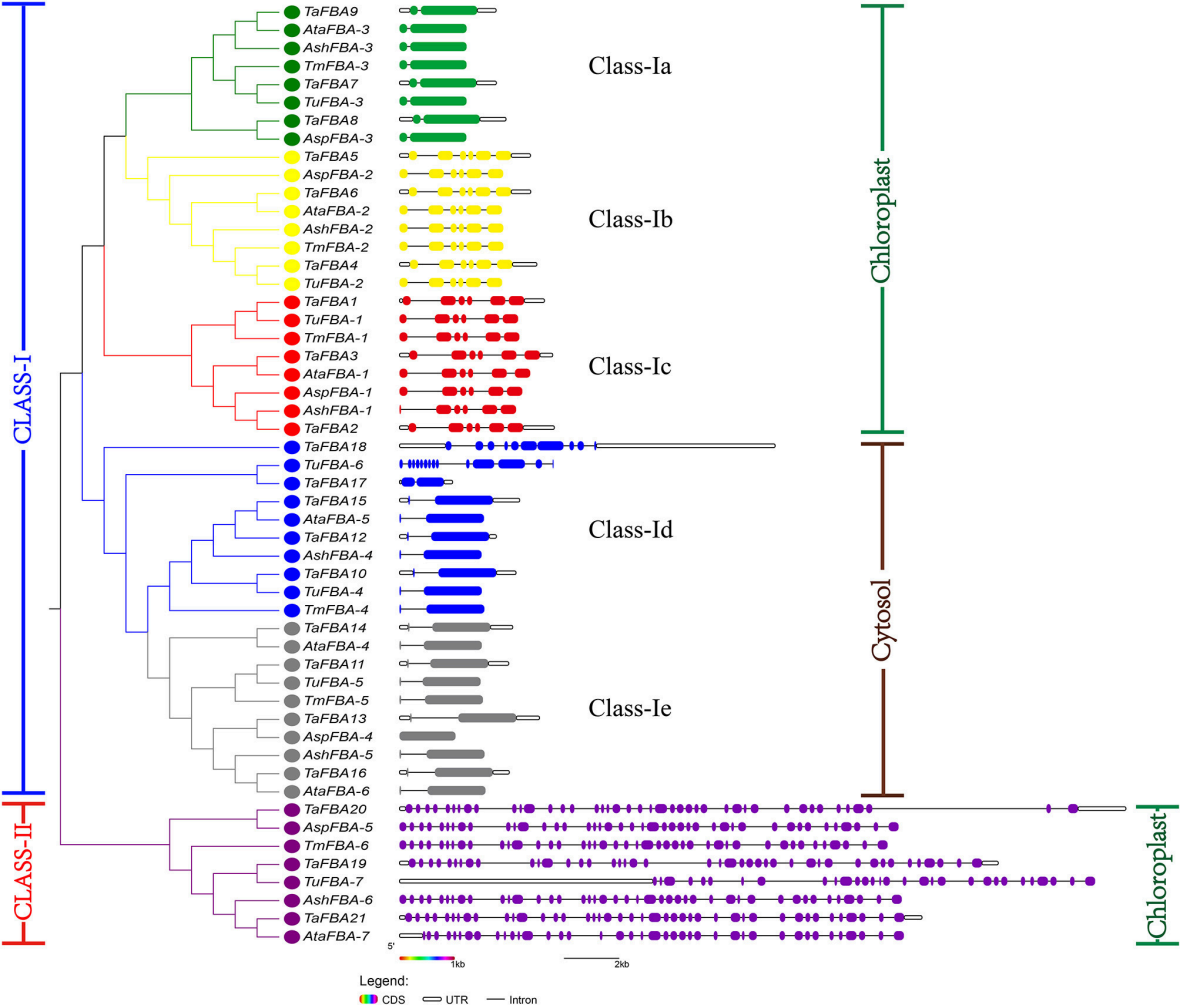
Exon–intron organization and the phylogenetic analysis of the FBA gene families in wheat and wheat relatives. Gene structures of *FBA*s analysed by GSDS. Exons are shown as Double-sided wedges, and different colours indicate different *FBA* gene groups. Introns are shown as thin lines. Untranslated regions (UTRs) are shown as blank boxes. The exon and intron sizes can be estimated using the scale at the bottom. *TaFBA, TmFBA, TuFBA, AspFBA, AshFBA, AtaFBA* indicated the *FBA* genes from *T. Aestivum, T. monococcum, T. urartu, A. speltoides, A. Sharonensis, A. tauschii* respectively. The unrooted tree was generated using MUSCLE in MEGA6.

For the investigation of the molecular evolution of the *TaFBA* gene family, 31 *FBA* genes from other closely related wheat species were identified: six *TmFBA* genes from *T. monococcum* (*A*^*m*^*A*^*m*^), seven *TuFBA* genes from *T. urartu* (*A*^*u*^*A*^*u*^), five *AspFBA* genes from *A. speltoides* (SS), six *AshFBA* genes from *A. sharonensis* (*S*^*sh*^*S*^*sh*^), and seven *AtaFBA* genes from *A. tauschii* (DD). The gene IDs and sequences are listed in Table S3, and Data Sheets TXT4, TXT 5. Then, an unrooted phylogenetic tree was constructed using MUSCLE in MEGA6 (Figure 2), and pairwise alignments were conducted using the hierarchical clustering method by MeV (Figure S2).

Owing to the transcript annotation of the wheat genome, the analysis and comparison of the structural features of the *FBA*s in different subfamilies were also conducted (Figure 2). The organization (number, order, and length) of the exons were mostly conserved within different FBA groups, whereas the introns and UTRs showed variable lengths and distribution. Based on the exon numbers and intron lengths, a total of 52 *FBA* genes from wheat and other closely related species could be divided into six independent subgroups: class Ia ~ class Ie and class II. 21 *TaFBA* genes belonged to different subgroups: *TaFBA7/8/9* genes in class Ia, *TaFBA4/5/6* genes in class Ib, *TaFBA1/2/3* genes in class Ic, *TaFBA10/12/15/17/18* genes in class Id, *TaFBA11/13/14/16* genes in class Ie, and *TaFBA19/20/21* genes in class II. Each subgroup contained five wheat relative *FBA* genes, except for six in class Ie. Class II *FBA* genes existed in both wheat and its closely related species.

Various patterns of exon-intron architecture were identified. For instance, class Ia genes and most cytoplasmic *TaFBA* genes had one intron, and class Ib and class Ic genes had five introns. On the other hand, class II *TaFBA* genes contained 41 introns because of the integration of F_bP_aldolase domain and other functional domains. Generally, a higher number of introns could cause alternative splicing and different splice variants, and regulate gene expression at the posttranscriptional level. Whether there are different transcripts of class II genes is thus worth exploring further.

In addition, the annotation of genome sequences suggested that different kinds of transposable elements (TEs) were distributed unevenly in the promoter and intron regions of the *TaFBA* genomes (Figure S3, Table S4) such as long terminal repeats (LTRs), short interspersed nuclear elements (SINEs), terminal inverted repeats (TIRs), and other TEs. Several TEs were detected in the *TaFBA19* and *TaFBA20* intron regions, which might influence RNA splicing.

### Evolutionary analysis of FBA proteins

**Figure 3:**
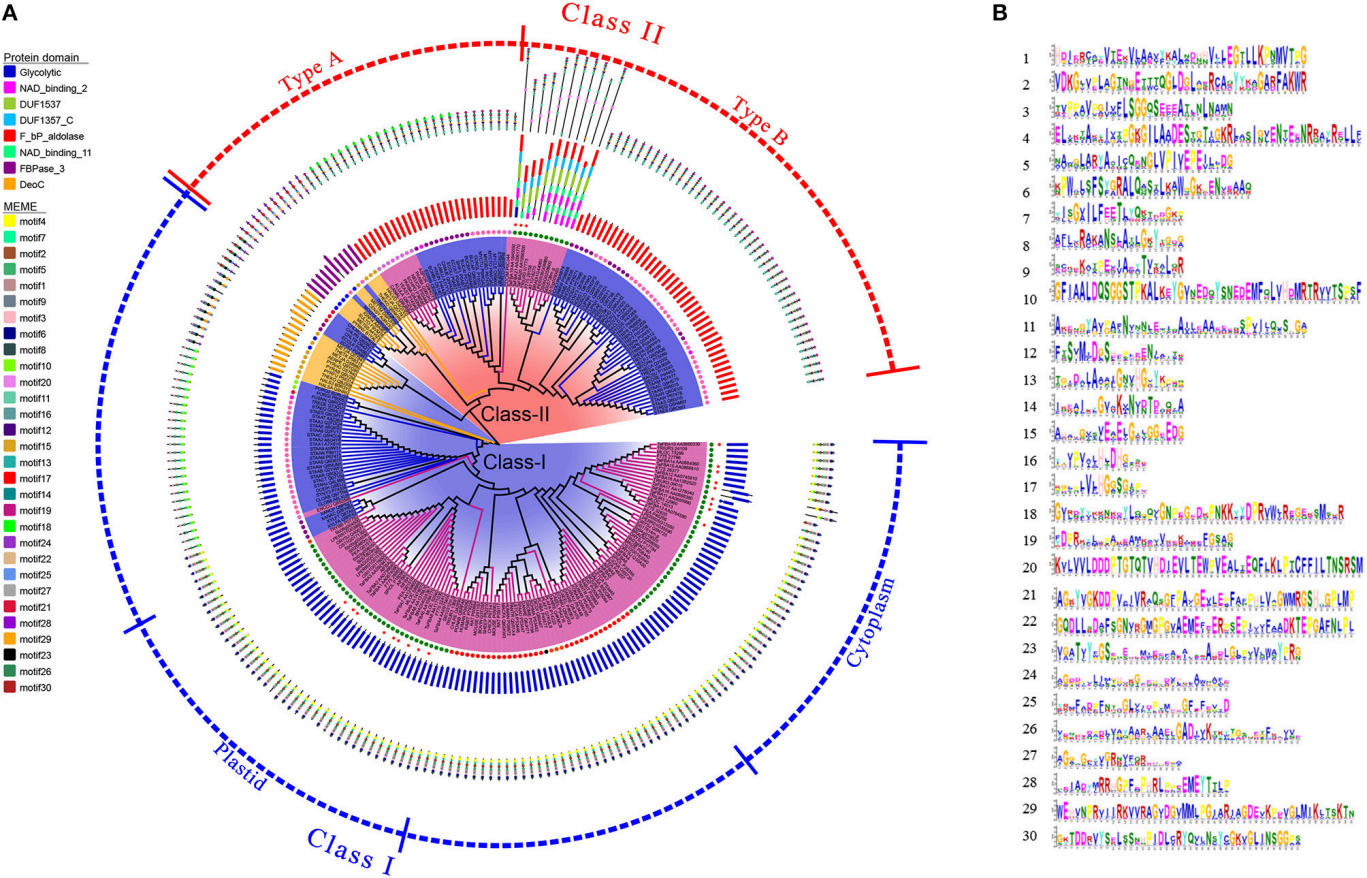
Phylogenetic and structure analysis of the FBA proteins. **(A)** The phylogenetic tree of FBA proteins constructed with Clustal Omega. Class I and class II *FBA* subfamilies are distinguished by blue and red colours respectively. with the full-length amino acid sequences of the 245 FBA proteins. 19 archaea FBAs are indicated by orange branches. 97 bacteria FBAs are indicated by blue branches. 129 eukaryota FBAs are indicated by purple branches. Red circle represents bilaterian. Green circle represents viridiplantae. Crimson circle represents chlamydia. Black circle represents *Dictyostelium discoideum*. Gold circle represents *Euryarchaeota*. Orchid circle represents fungi. Orange circle represents *Plasmodium*. Chartreuse circle represents *Porphyromonas gingivalis*. DarkMagenta circle represents Proteobacteria. Magenta circle represents Spirochaetales. Blue circle represents TACK group. Pink circle represents terrabacteria group. 21 TaFBAs were shown by little red circle. The annulus next to the tree represents the protein domains of FBAs and the outside annulus represents the MEME motifs. **(B)** Motif LOGOs of FBA proteins generated by MEME.

Using HMMER 3.0, a Uniprot KB/Swiss-Prot database search was performed using the query “fructose 1,6-bisphosphate aldolase,” and 211 sequences from different species were downloaded for FBA protein evolutionary analysis. Approximately 23 of these sequences were removed by pairwise comparison and the absence of complete adolase-type TIM domain. Together with plant FBA protein sequences identified by PHMMER from EnsemblPlants database, a total of 245 FBA proteins were obtained, which consisted of 19 archaeal FBAs, 97 bacterial FBAs, and 129 eukaryotic FBAs (listed in Table S5, Data Sheets TXT3, TXT6). Multiple primary sequences of FBA protein were aligned by using Clustal Omega, and a rooted ML phylogenetic tree was constructed by using MEGA6 with default parameters (Figure 3).

Evolutionary analysis revealed that the FBAs in various species were clearly classified into two groups, namely, classes I and II (Figure 3A). The class I FBAs in plants were classified into two subgroups, class I cFBAs, and class I CpFBA, occurring in plastids and the cytoplasm, respectively. The class I cFBAs in plants were more closely related to the FBAs in animals than class I CpFBAs. There were two subgroups (types A and B) in class II FBAs. Most FBAs in fungi belonged to type A, and 10 FBAs in plants (including TaFBA19~21) belonged to type B. Both plant cFBAs and CpFBAs were more distantly related to the FBAs in bacteria and fungi.

To investigate the conserved domain and residues of TaFBA proteins, we aligned the wheat FBA protein sequences and conserved sites by using Pfam and MEME. A total of 8 conserved protein domains and 30 motifs were defined (Figure 3). Most of the class I FBAs possessed a glycolytic protein domain, and the other class I FBAs possessed a DeoC (deoxyribose-phosphate aldolase) domain. Type A of class II FBAs possessed one F_bP_aldolase domain or FBPase_3 (fructose-1,6-bisphosphatase) domain. All of the type B of class II FBAs possessed an FBPase_3 domain, but the class II FBAs of plants possessed the other 4 domains, including NAD_binding_2 (NAD-binding domain of 6-phosphogluconate dehydrogenase), NAD_binding_11 (NAD-binding of NADP-dependent 3-hydroxyisobutyrate dehydrogenase), DUF1537 (putative sugar-binding N-terminal domain), and DUF1357_C domain (putative nucleotide-binding of sugar-metabolizing enzyme), which suggest that class II FBAs are NADP-dependent and probably participate in sugar sensing and signaling in plants. Several identical regions of plant FBAs were fairly conserved such as “EGTLLKPNMVTPG,” “GARFAKWR,” “LSGGQSEEEA,” “GILAADES,” “LVPIYEPE,” “RALQ,” “ILFEET,” and “RAKANS” for class I FBAs (Figure 3B). Among these regions, the “LSGGQSEEEA” (TIM _phosphate_binding superfamily), “LVPIYEPE,” and “ILFEET” matched well with that of animal/fungi/bacterial FBAs, suggesting that FBAs were highly conserved during evolution.

### Higher-order structure of the TaFBA proteins

**Figure 4:**
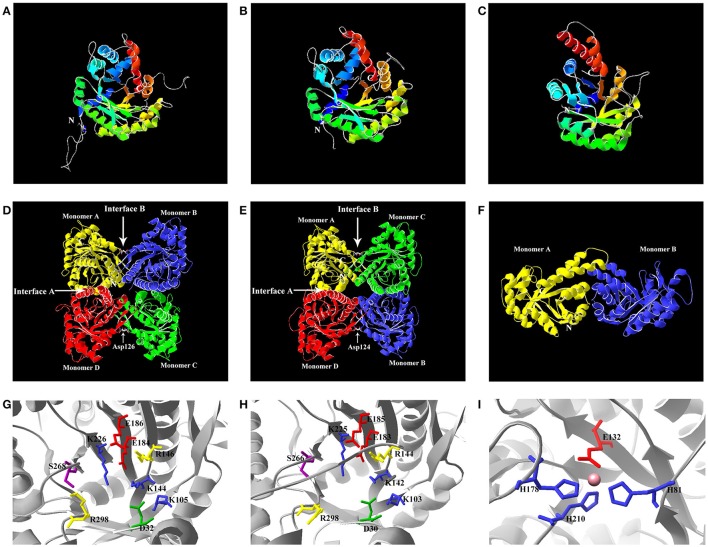
Predicted structures of TaFBA protein monomers and homo-oligomers. **(A)** Monomer TaFBA4. **(B)** Monomer TaFBA10. **(C)** Monomer TaFBA20. The C- and N-termini are labeled for monomer. **(D)** Tetrameric TaFBA4. **(E)** Tetrameric TaFBA10. **(F)** Dimeric TaFBA20. **(G)** Active site residues of TaFBA4. **(H)** Active site residues of TaFBA10. **(I)** Active site residues of TaFBA20. For **(A–F)**, Each monomer is colored separately, and A and B interfaces are indicated by arrows. The sites of the Asp126/124 substitutions are indicated on interface B, the C- and the N-termini are labeled for monomer A. The active site residues involved in substrate binding and catalysis are shown in different colours. For **(G–I)**, Lys226/225 **(G,H)** situated at the centre of the active region responsible for Schiff base formation, the filled circle of represents divalent metal cation in the three open protomers of TaFBA20.

The quaternary structure of three wheat FBA proteins (TaFBA4, TaFBA10, and TaFBA20) without N-terminal transit peptides were predicted by using SWISS-MODEL and I-TASSER (Figure 4). TaFBA4 and TaFBA10 belong to class I subfamily and could form tetramers based on their relatively high similarity to class I rabbit muscle aldolase (Blom and Sygusch, 1997) and human muscle aldolase (Dalby et al., 1999) respectively. TaFBA20 is a class II protein that is predicted to form dimers based on its similarity to class II aldolases of *Thermus aquaticus* (Izard and Sygusch, 2004).

Similar to other tetrameric FBAs, interfaces A and B were observed in the TaFBA4 and TaFBA10 tetramers (Figures 4D,E). In contrast to interface A, which comprised helix-packing interactions, interface B exhibited loop-loop interactions that were formed by buried hydrogen bonds. In TaFBA4, each interface B had six hydrogen bonds that were involved in the carboxylate “O” atoms of Asp126, interacting with the backbone amides of three consecutive residues (G124-L125-D126) (Sherawat et al., 2008) (Figures 4A–C). Different from TaFBA4, interface B of TaFBA10 was related to Asp124, which interacts with G122-F123-D124.

The catalytic residues in the active region of class I FBAs are highly conserved (Maurady et al., 2002). TaFBA4 contained nine catalytic residues, D32-K105-K144-R146-E184-E186-K226-S268-R298. The catalytic residues in TaFBA10 included D30-K103-K142-R144- E183-E185-K225-S266-R298. Although, class I TaFBAs had the same catalytic residue sequences, different conformations in the active sites would affect the spatial structures (Figures 4G,H). Additionally, class II TaFBAs were the same as the FBAs in *Thermus aquaticus*, which had active sites including imidazole rings of H81, H178, and H210, and the carboxylate of E132, and the sites also acted as divalent metal ion-binding sites (Figure 4I).

The amino acid sequence alignment of the TIM barrel domain (Figure S4) indicated that the active sites of TaFBA1~TaFBA16 (class I) were homologous to those of rabbit and human FBA isozymes, and the active sites among TaFBA19~21 (class II) corresponded to those of FBA isozymes in *Thermus aquaticus*. A few substitution mutations and deletion sites were observed in TaFBA17 and TaFBA18, and the catalytic active site sequence of TaFBA17 was D-K-T-P-K-S-R, and that of TaFBA18 was D-K-K-R-K-S-R.

### Cis-elements in the *TaFBA* gene promoters

**Figure 5:**
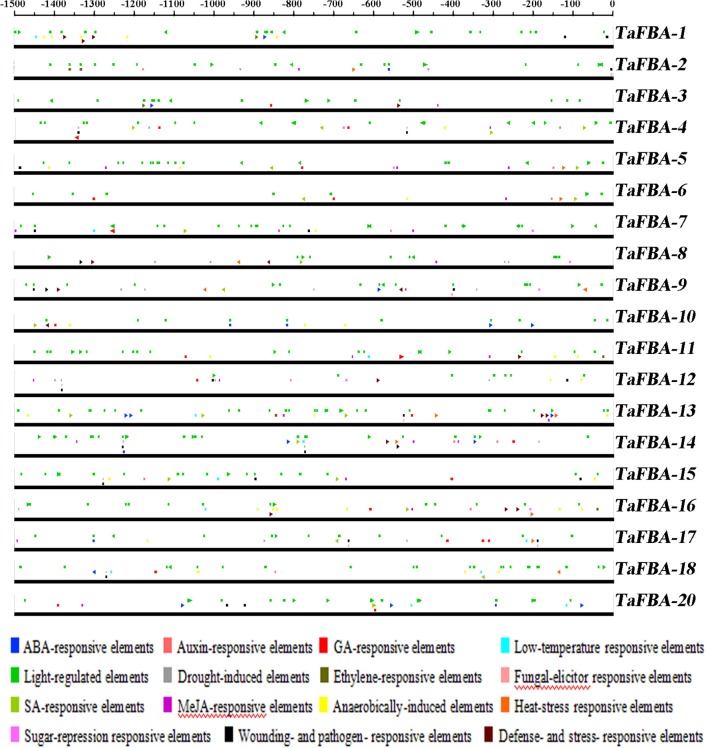
Phytohormone- and stress-responsive cis-elements in the promoter regions of TaFBA genes. 15 Putative ABA-responsive elements (ABRE), Auxin-responsive elements (ARE), GA-responsive elements (GARE), Low-temperature responsive elements (LTRE), Light-regulated elements (LRE), Drought-induced elements (DIRE), Ethylene-responsive elements(ERE), Fungal-elicitor responsive elements (FERE), SA-responsive elements (SARE), MeJA-responsive elements (MeJARE), Anaerobically-induced elements (AIRE), Heat-stress responsive elements (HSRE), Sugar-repression responsive elements (SSRE), Wounding-and pathogen-responsive elements (WPRE), and Defense- and stress-responsive elements (DSRE) core sequences in the 1,500-bp promoter regions of *TaFBA* genes were predicted using Plant CARE. The lines denote promoter sequences. The elements represented by different colours are indicated above the lines.

Cis-elements are important molecular switches that are involved in the regulation of gene transcription during plant growth, development, and responses to biotic and abiotic stresses. The 1.5-kb promoter sequences upstream of the 5′-UTR of 19 *TaFBA* genes (excluded *TaFBA19/21*) were identified from wheat genome sequences and used in plant cis-acting regulatory elements analysis in the PlantCARE database (Figure 5, Table 2).

**Table 2:** *Cis*-elements in the promoter region of 19 *TaFBA* genes.

**Gene**	***Cis*****-elements**														
	**ABRE**	**AIRE**	**ARE**	**DIRE**	**DSRE**	**ERE**	**FERE**	**GARE**	**HSE**	**LRE**	**LTRE**	**MeJARE**	**SARE**	**SRRE**	**WPRE**
*TaFBA1*	1	5			3	1				20	1				2
*TaFBA2*	1		1	1		2	1		1	17		1		1	1
*TaFBA3*	1			1		1		1		15		1			
*TaFBA4*		1	1	1			2	3		16		1	4	1	2
*TaFBA5*	2		1					1	1	18		3	2	1	1
*TaFBA6*		1	1					2	1	7		1	2		
*TaFBA7*		1	1					1		18	1	3	1	1	2
*TaFBA8*				4	2				1	9		1	1	1	1
*TaFBA9*	1			4	2		1		2	15		1	1	1	3
*TaFBA10*	4	3			1			1		10			1		
*TaFBA11*		3			1	1		2		19	1	2			
*TaFBA12*		1	1	2	1		1	1		7		2		1	2
*TaFBA13*	3	3			2		1	2	2	24	1	2	3		1
*TaFBA14*	3		2	3	1			1	1	18	1	3	1		3
*TaFBA15*		2	1				1	1		16	1	1	2		3
*TaFBA16*		6	1	1	3	1		1	1	13	1	1	1	1	
*TaFBA17*	1	1		2			2	3	1	12	1	1	1		2
*TaFBA18*	1	3	1				1	1	1	20	2		1		1
*TaFBA20*	4							2		13	2	1	1		2

*ABA-responsive elements (ABRE), Auxin-responsive elements(ARE), GA-responsive elements (GARE), Low-temperature responsive elements (LTRE), Light-regulated elements (LRE), Drought-induced elements (DIRE), Ethylene-responsive elements(ERE), Fungal-elicitor responsive elements (FERE), SA-responsive elements (SARE), MeJA-responsive elements (MeJARE), Anaerobically-induced elements (AIRE), Heat-stress responsive elements (HSRE), Sugar-repression responsive elements (SSRE), Wounding-and pathogen-responsive elements (WPRE) and Defense- and stress-responsive elements (DSRE)*.

Various putative environment stimulus-responsive cis-elements were identified (Table S6). Because *TaFBA* genes mostly possess multiple phytohormone and abiotic stress-responsive elements, we considered that the expression pattern of *TaFBA* genes could be regulated by various environmental factors. LREs were highly enriched in 18 *TaFBA* gene promoters. These findings indicate that the *TaFBA* genes might be involved in light responses.

### Expression profiles of the *TaFBA* genes in different tissues and developmental stages

**Figure 6:**
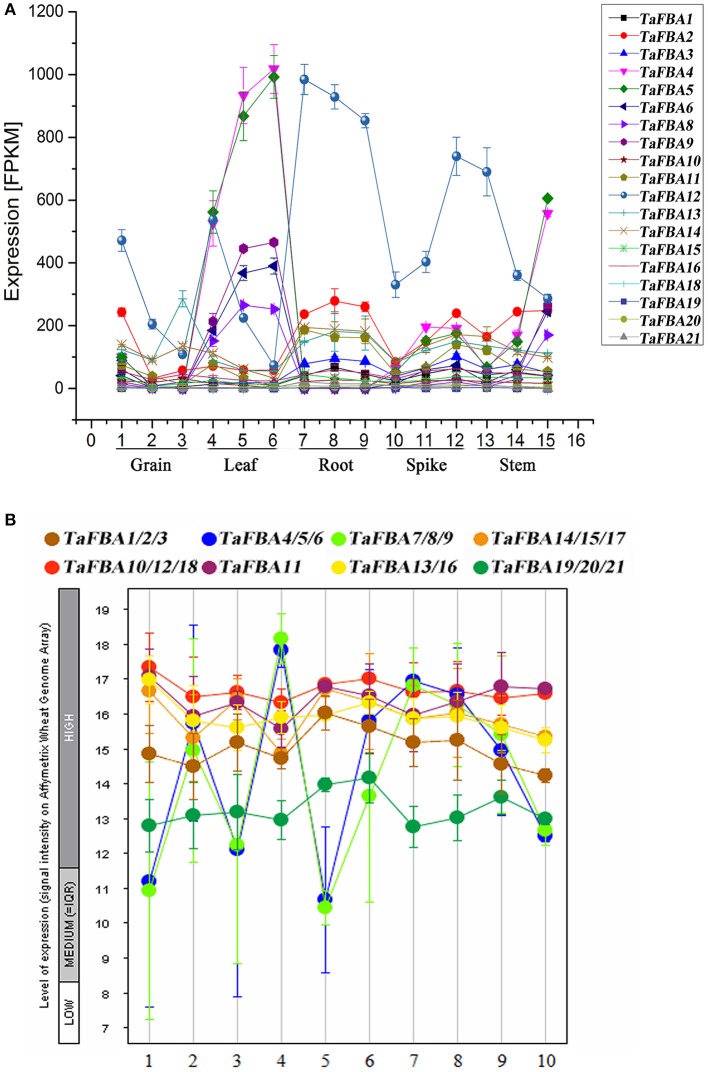
Expression profiles of TaFBAs in different tissues and different developmental stages. **(A)** Expression profiles of *TaFBA* genes in different tissues. **(B)** Expression profiles of *TaFBA* genes at different development stages. Number 1–10 indicate: 1-germination, 2-seedling growth, 3-tillering, 4-stem elongation, 5-booting, 6-inflorescence emergence, 7-anthesis, 8- milk, 9-dough, and10- ripening stages respectively.

The expression profiles of different tissues and developmental stages were downloaded from the WheatExp database and PLEXdb database respectively, and the corresponding probe ID of the *TaFBA* genes are shown in Table S7. Combined with the results of Genevestigator, we analyzed the spatiotemporal expression profiles of the members of the *TaFBA* gene family (Figure 6).

The expression of the *TaFBA* genes showed tissue specificity (Figure 6A). *TaFBA4-9* (class I *CpFBA* genes) showed similar expression patterns; these were upregulated in leaves and downregulated in the roots, particularly *TaFBA 4* and *5*. On the other hand, most class I *cFBA* genes were apparently upregulated in the roots. *TaFBA12* showed significantly higher expression levels in all tissues, particularly in the roots. *TaFBA19/20/21* (class II *CpFBA* genes) were slightly and constantly expressed in all tissues.

The wheat *TaFBA* genes were expressed at different developmental stages (Figure 6B). The expression level of *TaFBA10/12/18* was higher than that of the other genes, whereas that of *TaFBA19/20/21* was lower. The expression profiles of *TaFBA4/5/6* and *TaFBA7/8/9* varied with different developmental stages. Based on the results of differential expression and phylogenetic analyses, the *TaFBA* genes were classified into eight gene clusters: *TaFBA1/2/3, TaFBA4/5/6, TaFBA7/8/9, TaFBA14/15/17, TaFBA10/12/18, TaFBA11, TaFBA13/16*, and *TaFBA19/20/21*.

### Responses of *TaFBA* genes to light

**Figure 7:**
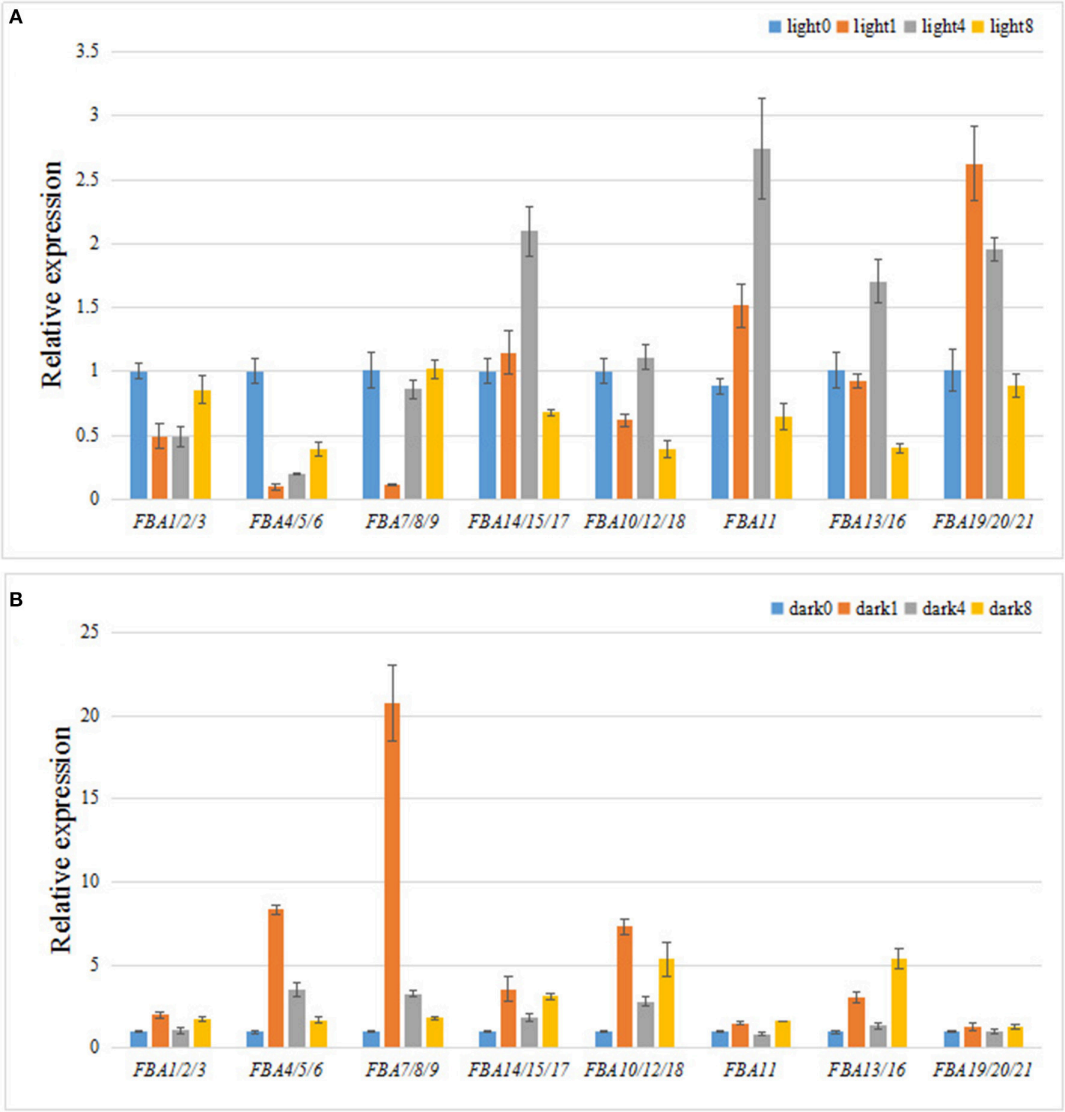
Differential expression analysis of TaFBA genes with light/dark treatments. **(A)** Light treatment. Total RNA was isolated from etiolated two-leaf seedling at 0, 1, 4, and 8 h after exposure to light. **(B)** Dark treatment. Two-leaf seedlings cultured under 16 h light/8 h dark were used and placed in darkness for 0, 1, 4, and 8 h after 24 h of continuous illumination. The data came from qRT-PCR analysis with 2^−ΔΔCt^ method.

Most *TaFBA* gene promoters were highly enriched with LREs (Figure 5 and Table 2). Thus, the expression patterns of the *TaFBA* genes under light and dark treatment were investigated by using qRT-PCR (Figure 7). *TaFBA1-9* (*CpFBA*) and *TaFBA10/12/18* (*cFBA*) were downregulated under short-term light treatment (Figure 7A), whereas the other *TaFBA*s were upregulated. Meanwhile, all *TaFBA* genes were immediately upregulated within 1 h of transferring from light to dark, especially *TaFBA4-9* (Figure 7B). *TaFBA14/15/17, TaFBA11, TaFBA13/16*, and *TaFBA19/20/21* were induced by light, whereas *TaFBA4/5/6, TaFBA7/8/9*, and *TaFBA10/12/18* were induced by short-term exposure to the dark. These results demonstrate that the LRE cis-elements of the *TaFBA* genes were not directly related to light responses. Therefore, FBA isozymes might play different roles in various biological processes involved in light signal transduction.

### Expression profiles of the *TaFBA* genes under abiotic stresses

**Figure 8:**
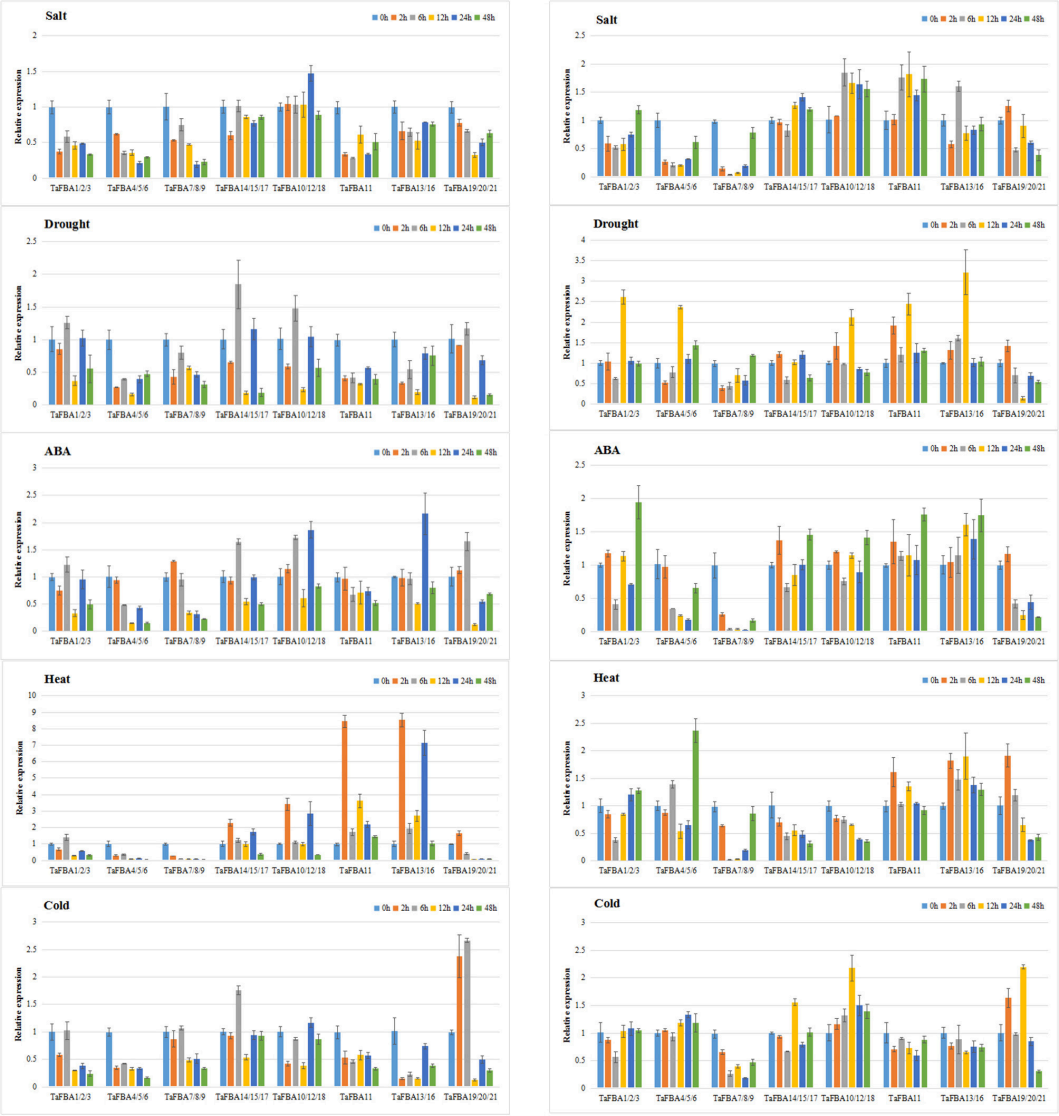
Expression profiles of TaFBA genes in shoots (A) and roots (B) under abiotic stresses. The data came from qRT-PCR analysis with 2^−ΔΔCt^ method. For each of the *TaFBA* genes, the relative expression value under normal condition was defined as 1.

The expression profiles of *TaFBA* gene clusters under different conditions (salt, drought, ABA, heat, and low temperature) were detected using qRT-PCR. The results revealed the complicated expression patterns of the *TaFBA* gene family in response to various abiotic stresses (Figure 8). The gene specific primers used for qRT-PCR are listed in Table S8.

In shoots (Figure 8A), the class I cytosol *TaFBA* genes displayed active responses to multiple abiotic stresses. The expression of *TaFBA11* and *TaFBA13/16* was significantly induced by high temperature. *TaFBA14/15/17* and *TaFBA10/12/18* were expressed during exposure to drought and ABA. Under salt stress, most *TaFBA* genes were downregulated except for *TaFBA10/12/18*. On the other hand, all class I *CpFBA* genes were apparently sensitive to multiple stresses, as indicated by a repression in expression. On the other hand, class II *CpFBA* genes *TaFBA19/20/21* were induced after short-term exposure to ABA, heat, and low temperature, implying the role of class II *TaFBA* genes in stress responses.

The expression pattern of *TaFBA* genes in roots (Figure 8B) was obviously different from shoots. ABA might lead to the similar change as drought to each gene cluster. Both *cFBA* and *CpFBA* were induced by drought, ABA, heat and cold, but only *cFBA* was upregulated by salt. Among *cFBA* genes, *TaFBA10/12/18* were up-regulated in multiple conditions except heat, *TaFBA11* and *TaFBA13/16* were up-regulated in different stresses except low temperature. In *CpFBA* genes, *TaFBA1/2/3* could be induced by drought and ABA, *TaFBA4/5/6* could be induced by drought and heat, and *TaFBA19/20/21* could be induced by all stresses, especially by heat and cold.

## Discussion

One key factor in the success of wheat as a global food crop is its adaptability to a wide variety of climatic conditions. This is attributed, in part, to its allohexaploid genome structure, which arose as a result of two polyploidization events (Mayer et al., 2014). In the present study, 21 *TaFBA* genes were identified in *Chinese spring*, including 18 class I FBA genes and 3 class II genes. Therefore, in-depth information on the FBA gene family has been annotated only in two plants: wheat and *Arabidopsis*. There are 8 *AtFBA* gene members in *Arabidopsis* (Lu et al., 2012), AtFBA1~3 occur in plastids, whereas AtFBA4~8 has been localized to the cytoplasm.

A total of 21 *TaFBA* genes have been localized to 12 chromosomes of 4 homoeologous groups, and most *TaFBA* gene loci have three copies distributed across three genomes of wheat. We have observed that *TaFBA4* may be in the wrong location (Chr.4AL instead of Chr.4AS) because of its high identity with the *TaFBA5/6* genes (97%). This could have been caused by chromosomal rearrangements, in that the proximal segment of Chr.4AL was co-linear with the proximal segments of Chr.4BS and 4DS (Devos et al., 1995). We believe that whole-genome duplication and segmental duplication may have contributed to the expansion of the *TaFBA* gene family. Gene duplication not only expands genome content but also diversifies gene function to ensure optimal adaptability and evolution of plants (Xu et al., 2012). Multiple isozymes ensure the essential function of FBA in the Calvin cycle, glycolysis, and gluconeogenesis, as well as lead to gene redundancy.

The *TaFBA* genes were most closely related to recent progenitors (Figure 2). For example, *TaFBA1* (located in Chr.3AS) was more closely related to *TmFBA-1* (97.7% identity) and *TuFBA-1* (98.1% identity), which corresponded to the hypothesis that *T. monococcum* and *T. urartu* may be the A-genome donor relatives (Dvorak et al., 1988; Dubcovsky et al., 1995). In addition, the *TaFBA* genes on the A and B genomes had higher identity to the allelic genes on the D genome relative to each other. For instance, the *TaFBA2* on Chr.3B was more similar to the *TaFBA3* on Chr.3D than to the *TaFBA1* on Chr.3A, and the *TaFBA4* on Chr.4A was more similar to *TaFBA6* on Chr.4D than that in Chr.*TaFBA5* on 4B (Figure 2). This observation was consistent with models of interline age hybridization in the *Triticeae* (Escobar et al., 2011; Civáň et al., 2013) and phylogenomics analyses that the D genome is a product of homoploid hybrid speciation between the A and B genome ancestors >5 million years ago (Marcussen et al., 2014; Mayer et al., 2014).

It has been proposed that certain Calvin cycle enzymes also function in glycolysis or gluconeogenesis, thus photosynthetic eukaryotes would be predicted to have cytosol enzymes derived from the eukaryotic host and chloroplast/plastid enzymes from the cyanobacterial endosymbiont (Rogers and Keeling, 2004; Allen et al., 2012). The evolutionary study on plant FBA genes is useful in investigating the origin of chloroplast and photosynthetic genes. Plaumann et al. (1997) reported that class II CpFBAs in *Euglena gracilis* and ascomycetes originated from eubacteria via endosymbiotic gene transfer. In the present study, we identified three different groups of FBAs in wheat, and each group appeared to have distinct evolutionary origin and function. Phylogenetic analysis (Figure 3) showed that class I CpFBAs in plants are closely related to the proteins of green algae, indicating that class I CpFBAs might have been derived from the endosymbiotic transfer of genes between green algae and its plant eukaryotic host. Class I cytosol FBAs are conserved in animals and plants, indicating that these might have been derived from a eukaryotic host. Class II TaFBAs were homologous to FBAs of bacteria and seemed to be of eubacterial origin. We speculated that class I CpFBAs, class I cFBAs, and class II CpFBAs might have been derived from green algae, eukaryotic hosts, and eubacteria, respectively, which was similar to the findings of Rogers and Keeling (2004) and Willard and Gibbs (1968).

Although, the exact function of each *TaFBA* gene is unclear, we observed that different subgroups of *TaFBA* genes have tissue, stage, and stress-response specificity. For instance, *TaFBA4/5/6* had more tissue and development specificity than the others, and *TaFBA10/12/18* could be induced by all kinds of abiotic stresses. In addition, *TaFBA4/5/6* was upregulated in leaf tissues (Figure 6A), might be involved in the regulation of enzyme activities in leaves, and play important regulatory roles during wheat development.

Several research studies on the responses of plant FBA genes to abiotic stress have been reported. Plastid aldose *AldP2* is upregulated by salt stress in *Nicotiana* (Yamada et al., 2000). Abiotic stimuli could rapidly trigger a significant induction of *FBA* genes in *Sesuvium portulacastrum* (Fan et al., 2009). An FBA-dependent fructose signaling pathway acts downstream of the abscisic acid pathway in *Arabidopsis thaliana* (Cho and Yoo, 2011). Class I cFBAs in *Medicago sativa* has been identified to have an NMH7 MADS domain (Páez-Valencia et al., 2008), and FBA is involved in the control of RNA polymerase III-directed transcription (Cieśla et al., 2014). These results demonstrate that FBA displays an important role in responses to abiotic stress in plants, but the difference between CpFBAs and cFBAs is unkown.

In the present study, we investigated the responses of wheat *FBA* genes family to salt, drought, ABA, heat, and low temperature. The results indicate that most *TaFBA* genes in the roots showed higher expression levels than in shoots under abiotic conditions. Most cytosol *TaFBA* genes, especially *TaFBA10/12/18* and *TaFBA13/16*, could be induced by abiotic stresses, whereas most class I CpFBAs in wheat might be sensitive to stress conditions. Furthermore, the expression level of class II gene *TaFBA19/20/21* was immediately upregulated under stresses in both roots and shoots. These results suggest that cytosolic *TaFBAs* and class II *TaFBA* genes could play important roles in the responses to abiotic stresses, which coincided with the findings of previous studies. We plan to conduct additional experiments to investigate the molecular mechanism underlying TaFBA biofunction.

The FBA of the Benson-Calvin cycle is not identical to the analogous enzymes of the Embden-Meyerhof-Parnas pathway, and there exists no cross-reactivity between the cytosol and chloroplast aldolase (Krüger and Schnarrenberger, 1983). Each member of the FBA gene family might thus have different biochemical activity. *TaFBA4/5/6* and *TaFBA7/8/9* genes (Class I CpFBA) could be induced by short-term exposure to darkness and repressed by photostimulation, but *TaFBA14/15/17, TaFBA11*, and *TaFBA13/16* genes (Class I cFBA) showed the contrary response to light/dark (Figure 7). The discrepancies in expression profiles are indicative of the multiple functions of different *TaFBA* genes, and FBAs might play a more complicated role in the regulation of glycolysis and carbon concentration. According to the predicted protein structure, the active sites of dimeric class II FBAs also serve as the divalent metal cation-binding sites (Figure 4). Under light irradiation, photosynthesis induces the production of NADPH, and the light-stimulated H^+^ shift is countered by Mg^2+^ and other cations moving from thylakoids to stroma, which in turn activate chloroplast class II FBAs. Hence, we considered that class II FBAs are more active in the Calvin cycle.

Different sugar signals generated by photosynthesis and carbon metabolism in light/dark condition might modulate multiple HXK-dependent and HXK-independent pathways and could regulate transcription, translation, protein stability, and enzymatic activity (Rolland et al., 2006), and sugar signals also affect the activities of FBA. There is growing interest in carbon-concentrating mechanisms (CCMs) in plants. Because FBA is an important enzyme involved in the Calvin cycle, improving the activity of the enzyme could certainly boost CO_2_ concentrations in plant green tissues.

In sum, the *TaFBA* gene family has significant biofunctions during plant development, metabolism, and abiotic stress responses. The *TaFBA* genes may be utilized in the development and selection of high-yield and multi-resistant wheat cultivars.

## Author contributions

Chromosomal location and express level experiment: GL and XG. Bioinformatics analysis and data processing: GL, LPX, and LX. Cultivating the wheat plants, sample collection, and mRNA extraction: YY and YT. Plant material, experiment design, discussion writing, and manuscripts review: CX, XZ, XP, AG, and HX.

### Conflict of interest statement

The authors declare that the research was conducted in the absence of any commercial or financial relationships that could be construed as a potential conflict of interest.

## Supplementary material

Figure S1 Chromosomal location of TaFBA genes. **(A)** *TaFBA1, 2, 3, 4, 7, 8, 11, 19*, and *20* genes were mapped on different gene loci by wheat_2014_90K SNP gene chip. **(B)** *TaFBA7, 8, 10, 12, 14*, and *15* genes were mapped by *Chinese_Spring*_Deletion gene chip.

Figure S2 Pairwise alignments of FBA genes of wheat and wheat relatives. The picture was generated by MeV using hierarchical clustering method. The number above the dash line are the identity between cDNA sequences, and the number below the dash line represent the identity between gDNA sequences.

Figure S3 Transposable elements on TaFBA genomic DNA. Gene structures of *FBA*s analyzed by GSDS. Exons are shown as blue rectangles; introns are shown as thin lines. Untranslated regions (UTRs) are shown as double-sided wedges. Promoters are shown as light-blue rectangles. Long terminal repeat(LTRs) are shown as purple rectangles. Terminal inverted repeat (TIRs) are shown as red rectangles. Short interspersed nuclear element (SINEs) are shown as green rectangles. Other Transposable element (TEs) are shown as pink rectangles.

Figure S4 The alignment of FBA protein sequences among wheat and other species. **(A)** The activity sites among the TaFBA1~18 corresponded to those sites among FBA isozymes of rabbit and human. The activity sites are indicated in black frames. **(B)** The activity sites among TaFBA19~21 corresponded to those sites among FBA isozymes of *Thermus aquaticus*. The activity sites are indicated in black frames. The red stars represent substitution mutations and deletion sites.

Table S1 Pairwise alignments of TaFBA genes. The number above the dash line are the identity between cDNA sequences, and the number below the dash line represent the identity between amino acid sequences.

Table S2 Gene-specific primers for mapping and cloning.

Table S3 *FBA* genes of wheat and wheat relatives.

Table S4 Annotation of transposable elements on *TaFBA* genes.

Table S5 HMM search hits of FBA proteins.

Table S6 Annotation of cis-regulatory elements involved in phytohormone and abiotic stress response of plants.

Table S7 BLAST results of *FBA* genes from PLEXdb.

Table S8 Primers used for qRT–PCR.

DOC1 Promoter sequences of *TaFBA* genes.

TXT1 The TaFBA cDNA sequences.

TXT2 The TaFBA gDNA sequences.

TXT3 The TaFBA protein sequences.

TXT4 The FBA cDNA sequences of wheat relatives.

TXT5 The FBA gDNA sequences of wheat relatives.

TXT6 The FBA protein sequences of other species.

## Abbreviations

ADP-RF: ADP ribosylation factor
ALD: Aldolase
cFBA: cytosol fructose-1,6-bisphosphate aldolase
CpFBA: Chloroplast/plastid Fructose-1, 6-bisphosphate aldolase
DeoC: Deoxyribose-phosphate aldolase
DHAP: Dihydroxyacetone phosphate
DUF1357_C: Putative nucleotide-binding domain of sugar-metabolizing enzyme
DUF1537: Putative sugar-binding N-terminal domain
EDTA: Ethylene diamine tetraacetic acid
F_bP_aldolase: Fructose-bisphosphate aldolase class-II domain
FBA/FBPA: Fructose-1, 6-bisphosphate aldolase
FBP: Fructose-1, 6-bisphosphate
FBPase_3: Fructose-1, 6-bisphosphatase
GAP: Glyceraldehyde-3-phosphate
hiTAIL-PCR: high-efficient thermal asymmetric interlaced PCR
HXK: Hexokinase
LTR: Long terminal repeat
NAD: Nicotinamide adenine dinucleotide
NAD_binding_11: NAD-binding domain of NADP-dependent 3-hydroxyisobutyrate dehydrogenase
NAD_binding_2: NAD binding domain of 6-phosphogluconate dehydrogenase
NADP: Nicotinamide adenine dinucleotide phosphate
qRT-PCR: quantitative real-time PCR
SFBA: Sedoheptulose/Fructose-bisphosphate aldolase
SINE: Short interspersed nuclear element
SuBP: Sedoheptulose-1, 7-bisphosphate
TaFBA: Fructose-1, 6-bisphosphate aldolase of *Triticum aestivum* L.
TE: Transposable element
TIM: Triose-phosphate isomerase
TIR: Terminal inverted repeat.
