# Supplementary figures and images for: Molecular Characterization, Gene Evolution, and Expression Analysis of the Fructose-1, 6-bisphosphate Aldolase (FBA) Gene Family in Wheat (Triticum aestivum L.)
Source: Front Plant Sci. 2017 Jun 14;8:1030. doi: 10.3389/fpls.2017.01030 (PMC5470051; doi:10.3389/fpls.2017.01030)

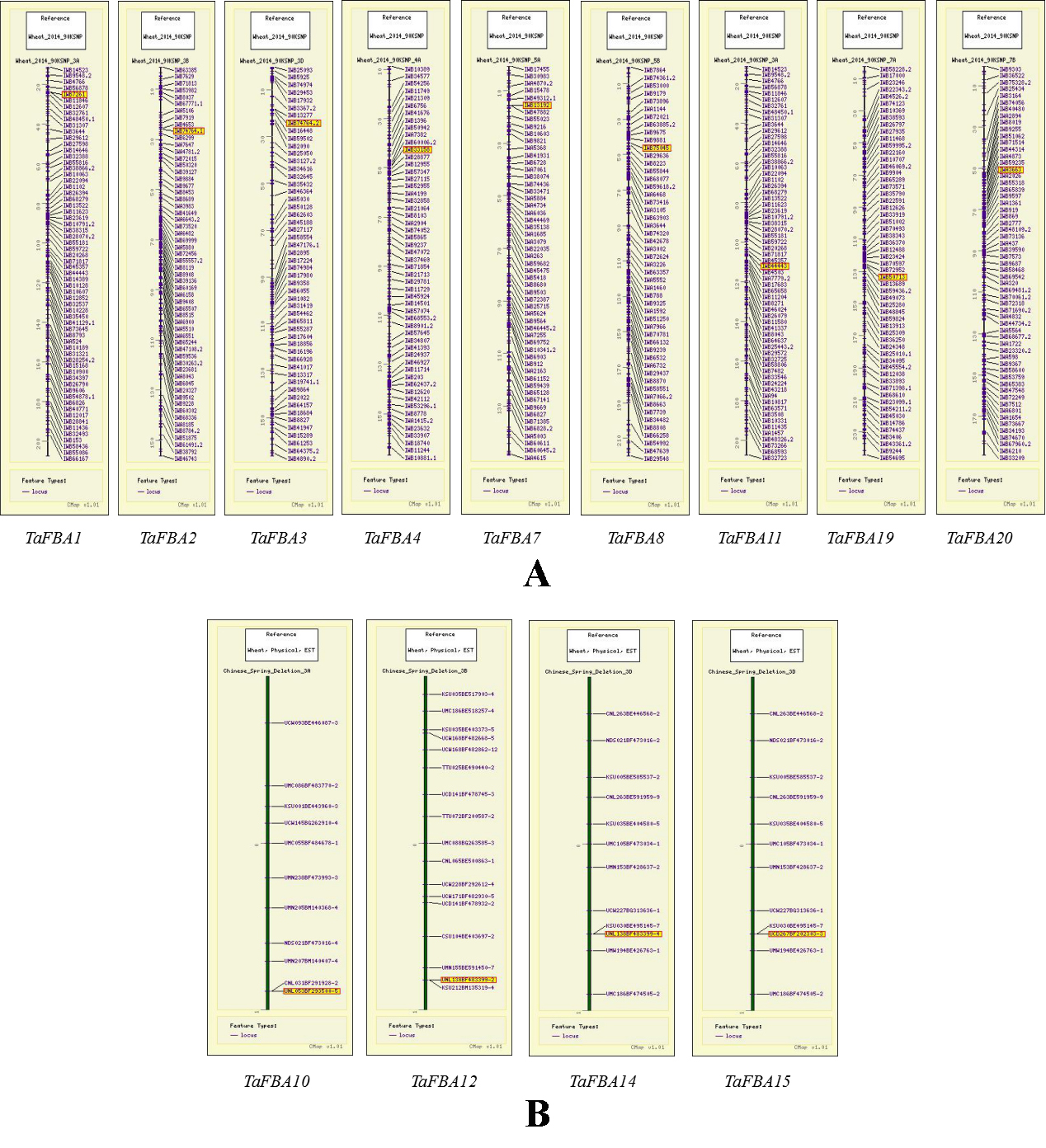

Supplement: Figure S1 — Chromosomal location of TaFBA genes. (A) TaFBA1, 2, 3, 4, 7, 8, 11, 19, and 20 genes were mapped on different gene loci by wheat_2014_90K SNP gene chip. (B) TaFBA7, 8, 10, 12, 14, and 15 genes were mapped by Chinese_Spring_Deletion gene chip. [file Image1.JPEG]

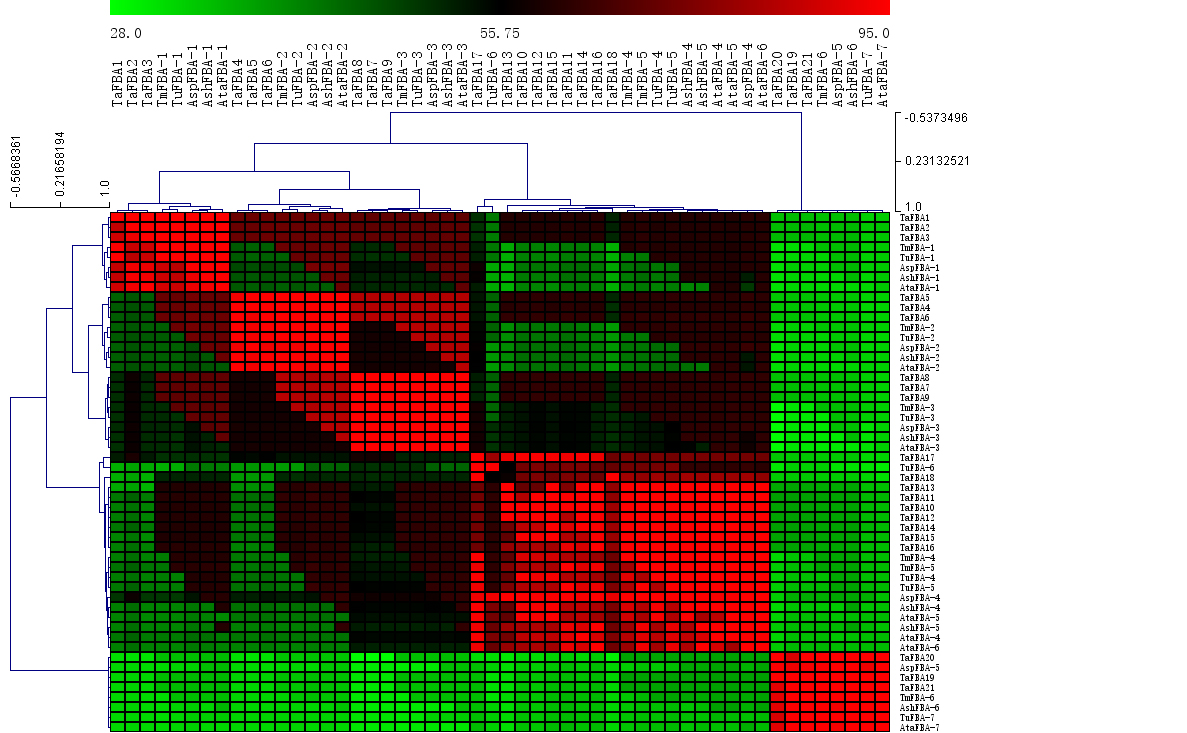

Supplement: Figure S2 — Pairwise alignments of FBA genes of wheat and wheat relatives. The picture was generated by MeV using hierarchical clustering method. The number above the dash line are the identity between cDNA sequences, and the number below the dash line represent the identity between gDNA sequences. [file Image2.JPEG]

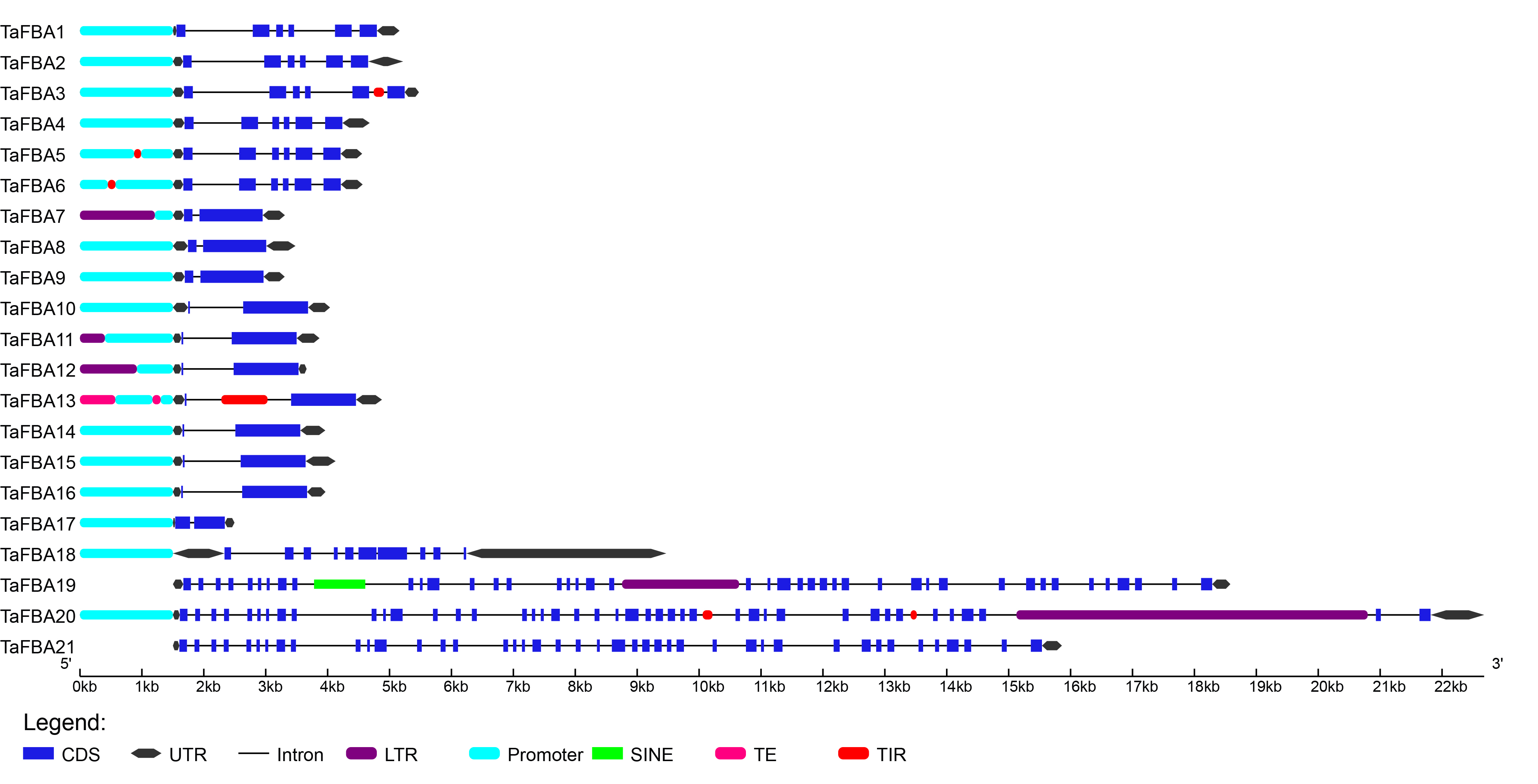

Supplement: Figure S3 — Transposable elements on TaFBA genomic DNA. Gene structures of FBAs analyzed by GSDS. Exons are shown as blue rectangles; introns are shown as thin lines. Untranslated regions (UTRs) are shown as double-sided wedges. Promoters are shown as light-blue rectangles. Long terminal repeat(LTRs) are shown as purple rectangles. Terminal inverted repeat (TIRs) are shown as red rectangles. Short interspersed nuclear element (SINEs) are shown as green rectangles. Other Transposable element (TEs) are shown as pink rectangles. [file Image3.JPEG]

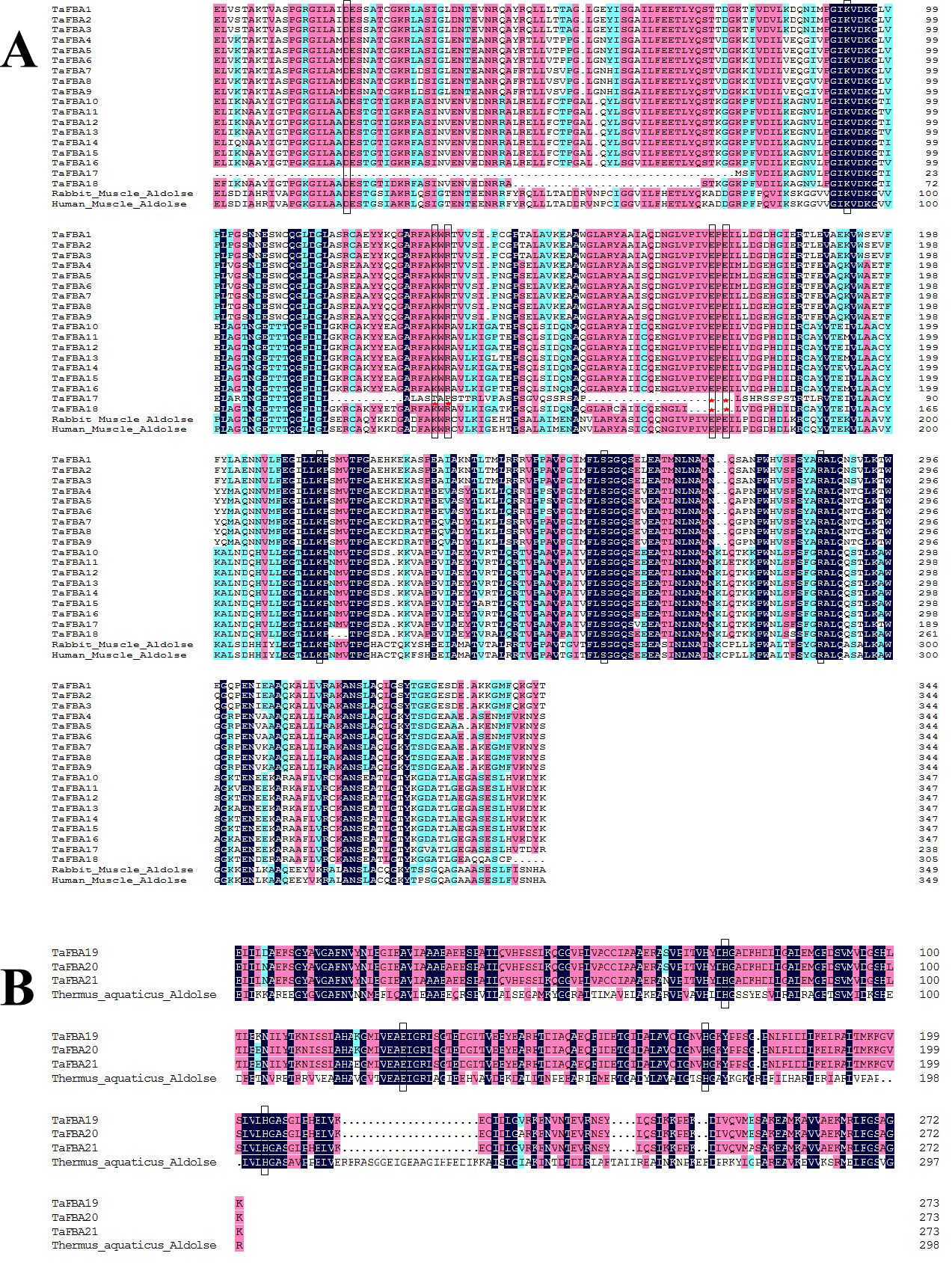

Supplement: Figure S4 — The alignment of FBA protein sequences among wheat and other species. (A) The activity sites among the TaFBA1~18 corresponded to those sites among FBA isozymes of rabbit and human. The activity sites are indicated in black frames. (B) The activity sites among TaFBA19~21 corresponded to those sites among FBA isozymes of Thermus aquaticus. The activity sites are indicated in black frames. The red stars represent substitution mutations and deletion sites. [file Image4.JPEG]
