# Supplementary material for: Molecular Characterization, Gene Evolution, and Expression Analysis of the Fructose-1, 6-bisphosphate Aldolase (FBA) Gene Family in Wheat (Triticum aestivum L.)
Source: Front Plant Sci. 2017 Jun 14;8:1030. doi: 10.3389/fpls.2017.01030 (PMC5470051; doi:10.3389/fpls.2017.01030)
Supplement: Table S1 — Pairwise alignments of TaFBA genes. The number above the dash line are the identity between cDNA sequences, and the number below the dash line represent the identity between amino acid sequences. [file Table1.DOCX]

**Table S1. Pairwise alignments of *TaFBA* genes.**

|  | <20 | 24 | 28 | 32 | 36 | 40 | 44 | 48 | 52 | 56 | 60 | 64 | 68 | 72 | 76 | 80 | 84 | 88 | 92 | 96 | 100 |
| --- | --- | --- | --- | --- | --- | --- | --- | --- | --- | --- | --- | --- | --- | --- | --- | --- | --- | --- | --- | --- | --- |
|  |  |  |  |  |  |  |  |  |  |  |  |  |  |  |  |  |  |  |  |  |  |
|  |  |  |  |  |  |  |  |  |  |  |  |  |  |  |  |  |  |  |  |  |  |
| Protein.  Identity.  Nucleotide. | *TaFBA1* | *TaFBA2* | *TaFBA3* | *TaFBA4* | *TaFBA5* | *TaFBA6* | *TaFBA7* | *TaFBA8* | *TaFBA9* | *TaFBA10* | *TaFBA11* | *TaFBA12* | *TaFBA13* | *TaFBA14* | *TaFBA15* | *TaFBA16* | *TaFBA17* | *TaFBA18* | *TaFBA19* | *TaFBA20* | *TaFBA21* |
| TaFBA1 |  | 96.3 | 97.9 | 72.4 | 72.2 | 72.6 | 70.6 | 70.6 | 70.5 | 61.5 | 62 | 61.5 | 61.5 | 61.1 | 61 | 62.1 | 61 | 50.7 | 36.2 | 35.9 | 35.8 |
| TaFBA2 | 98.7 |  | 97.4 | 72.1 | 72 | 72.5 | 70.6 | 70.4 | 70.5 | 61.2 | 61.7 | 61.2 | 61 | 60.9 | 61 | 61.7 | 60.6 | 50.7 | 36.1 | 35.8 | 35.7 |
| TaFBA3 | 99.5 | 99.2 |  | 72.2 | 72 | 72.4 | 71.1 | 71 | 70.9 | 61.2 | 61.8 | 61.2 | 61.2 | 60.8 | 61 | 61.9 | 60.6 | 50.4 | 35.7 | 35.4 | 35.3 |
| TaFBA4 | 74.5 | 74.7 | 74.5 |  | 97.2 | 97.9 | 83.9 | 83.9 | 83.9 | 62.8 | 62.8 | 62.8 | 62.3 | 62.3 | 62.2 | 62.1 | 61.3 | 49.5 | 37.4 | 36.9 | 37.2 |
| TaFBA5 | 74.5 | 74.7 | 74.5 | 98.7 |  | 97.4 | 83.5 | 83.2 | 83.4 | 63.1 | 63.4 | 63.1 | 63 | 62.8 | 62.8 | 62.7 | 61.6 | 50.5 | 37.1 | 36.7 | 37.1 |
| TaFBA6 | 74.5 | 74.7 | 74.5 | 99.5 | 98.7 |  | 84.2 | 83.9 | 84.1 | 63.7 | 63.8 | 63.7 | 63.7 | 63.2 | 63.2 | 63.2 | 62 | 50.5 | 37.5 | 36.9 | 37.3 |
| TaFBA7 | 74.4 | 74.5 | 74.4 | 94 | 93.5 | 94 |  | 99 | 99.1 | 63.4 | 63.2 | 63.4 | 63.2 | 63 | 63.6 | 62.7 | 60.7 | 50 | 35.7 | 35.2 | 35.7 |
| TaFBA8 | 74.2 | 74.3 | 74.2 | 93.8 | 93.2 | 93.8 | 99.7 |  | 99.1 | 63.8 | 63.6 | 63.8 | 63.4 | 63.4 | 63.9 | 63.1 | 61.5 | 50.4 | 35.6 | 35 | 35.6 |
| TaFBA9 | 74.4 | 74.5 | 74.4 | 94 | 93.5 | 94 | 100 | 99.7 |  | 63.6 | 63.2 | 63.6 | 63.2 | 63.2 | 63.6 | 62.7 | 60.7 | 50.3 | 35.3 | 34.8 | 35.3 |
| TaFBA10 | 56.5 | 56.5 | 56.5 | 56.3 | 55.7 | 56.3 | 56 | 56.3 | 56 |  | 94.9 | 100 | 95 | 97.7 | 97.6 | 94.5 | 94.5 | 80.2 | 37 | 37 | 37 |
| TaFBA11 | 56.8 | 56.8 | 56.8 | 56.3 | 55.7 | 56.3 | 56 | 56.3 | 56 | 97.2 |  | 94.9 | 96.8 | 94.6 | 95.2 | 96.7 | 92.4 | 77.9 | 35.7 | 35.7 | 35.6 |
| TaFBA12 | 56.5 | 56.5 | 56.5 | 56.3 | 55.7 | 56.3 | 56 | 56.3 | 56 | 100 | 97.2 |  | 95 | 97.7 | 97.6 | 94.5 | 94.5 | 80.2 | 37 | 37 | 37 |
| TaFBA13 | 57.1 | 57.1 | 57.1 | 56.5 | 56 | 56.5 | 56.3 | 56.5 | 56.3 | 97.5 | 99.2 | 97.5 |  | 94.4 | 94.8 | 97.7 | 92.2 | 78.2 | 36.2 | 36.1 | 36.1 |
| TaFBA14 | 56.5 | 56.5 | 56.5 | 56 | 55.4 | 56 | 55.7 | 56 | 55.7 | 99.4 | 96.9 | 99.4 | 97.2 |  | 97.2 | 94 | 94 | 80.1 | 36.7 | 36.6 | 36.6 |
| TaFBA15 | 56.5 | 56.5 | 56.5 | 56.3 | 55.7 | 56.3 | 56 | 56.3 | 56 | 99.7 | 96.9 | 99.7 | 97.2 | 99.2 |  | 94.2 | 94 | 79.3 | 36.8 | 36.7 | 36.7 |
| TaFBA16 | 57.1 | 57.1 | 57.1 | 56.5 | 56 | 56.5 | 56.3 | 56.5 | 56.3 | 97.2 | 99.2 | 97.2 | 99.4 | 96.9 | 96.9 |  | 92 | 78 | 36.9 | 36.8 | 36.8 |
| TaFBA17 | 48.5 | 48.5 | 48.5 | 45.5 | 44.7 | 45.5 | 46 | 46.4 | 46 | 82.4 | 80.8 | 82.4 | 81.6 | 82 | 82 | 81.6 |  | 91.5 | 34.5 | 34.5 | 34.9 |
| TaFBA18 | 47.7 | 48 | 47.7 | 46.1 | 45.8 | 45.8 | 46.5 | 46.8 | 46.5 | 90.9 | 89 | 90.9 | 89.3 | 90.3 | 90.6 | 89 | 75.5 |  | 33.4 | 33.3 | 33.3 |
| TaFBA19 | 22.7 | 22.3 | 22.4 | 20.5 | 20.7 | 20.5 | 21.7 | 22 | 21.7 | 22.3 | 22 | 22.3 | 22.3 | 22 | 22.3 | 22 | 22.5 | 18.5 |  | 98.3 | 98.5 |
| TaFBA20 | 22.4 | 22 | 22.1 | 20.2 | 20.5 | 20.2 | 21.4 | 21.7 | 21.4 | 22 | 21.7 | 22 | 22 | 21.7 | 22 | 21.7 | 22.5 | 18.7 | 98.2 |  | 98.4 |
| TaFBA21 | 22.4 | 22 | 22.1 | 20.2 | 20.5 | 20.2 | 21.4 | 21.7 | 21.4 | 22 | 21.7 | 22 | 22 | 21.7 | 22 | 21.7 | 22.5 | 18.7 | 98.2 | 98.6 |  |

The number above the dash line are the identity between cDNA sequences, and the number below the dash line represent the identity between amino acid sequences.
