# Supplementary material for: Molecular Characterization, Gene Evolution, and Expression Analysis of the Fructose-1, 6-bisphosphate Aldolase (FBA) Gene Family in Wheat (Triticum aestivum L.)
Source: Front Plant Sci. 2017 Jun 14;8:1030. doi: 10.3389/fpls.2017.01030 (PMC5470051; doi:10.3389/fpls.2017.01030)
Supplement: Table S2 — Gene-specific primers for mapping and cloning. [file Table2.DOCX]

**Table S2. Gene-specific primers for mapping and cloning.**

| Gene | Primer | Primer Sequences |
| --- | --- | --- |
| TaFBA1 | FBA1-F | CCTAGTGCGTGCCTTGAGA |
|  | FBA1-R | CGGAGAGGAACTGCGGAAT |
| TaFBA2 | FBA2-F | ATCCTCGTAGCTCCGCCTA |
|  | FBA2-R | TCATCGTCGCCTCCAGTTC |
| TaFBA3 | FBA3-F | GTTTGTTTAGTTTGGGTTGG |
|  | FBA3-R | AAGACCATACCATGATTCCA |
| TaFBA4 | FBA4-F | AACGGTTCATCAGCCAAG |
|  | FBA4-R | ACCAACAACGACCTTCAG |
| TaFBA5 | FBA5-F | CGTCTGCTGCTCTCCTCAA |
|  | FBA5-R | CTCCGTCCATCTCTTCCATCA |
| TaFBA6 | FBA6-F | GGAGAAGTCACATACACCAT |
|  | FBA6-R | AGGATGAGGAAGAGCAAGA |
| TaFBA7 | FBA7-F | GCCGCAGGTGATGATGTCA |
|  | FBA7-R | GAGACGAACCAACAAACGCA |
| TaFBA8 | FBA8-F | GGCAACCAGAGAAGGAAG |
|  | FBA8-R | ATGGCACAGTCCAGTAGA |
| TaFBA9 | FBA9-F | CTGGAGTGAAGAAAGTACAATC |
|  | FBA9-R | CGGACACAAGATATGAGAAC |
| TaFBA10 | FBA10-F | GTAGCACCAACCATAAGTCCATAA |
|  | FBA10-R | GCCAGTATCATCATTCGCACAA |
| TaFBA11 | FBA11-F | GACTTGACTGCCTTCTGATTGAT |
|  | FBA11-R | ATGATTCTGCTACTGCTTCTTCC |
| TaFBA12 | FBA12-F | TTGCTCTTCTGCGTGCTTAG |
|  | FBA12-R | CTCTCGTGGTGTTGTTAGGATAG |
| TaFBA13 | FBA13-F | CAGTGTCGTCAAGGAAGATGTC |
|  | FBA13-R | TGTGGTGCGGTTATGTTGTG |
| TaFBA14 | FBA14-F | CGAGGTGTGGAGAGGATGAG |
|  | FBA14-R | AAGGAAGGAAGTGGCAGTAGAA |
| TaFBA15 | FBA15-F | GCGTGCGATTGCTACCTTG |
|  | FBA15-R | CCTTCTCCTCATTCTCCGTCTT |
| TaFBA16 | FBA16-F | AATCCGTAGTAGAACCGACCAT |
|  | FBA16-R | CACCACACCGAAGGCAGAT |
| TaFBA17 | FBA17-F | CGCCTCCAATCGGTAGACA |
|  | FBA17-R | CAGTGAGCGACAGCAGTATC |
| TaFBA18 | FBA18-F | CGCCTCCAATCGGTAGACA |
|  | FBA18-R | AAGCCAAGCAAGACACAAGAAT |
| TaFBA19 | FBA19-F | CGTCTCCATTCTTCTGCCATC |
|  | FBA19-R | CTAAGCCATCCACTGTTCCATC |
| TaFBA20 | FBA20-F | GTGGACCGAACTTGAGACTTG |
|  | FBA20-R | GTGACGAGATGCTTGCTGAG |
| TaFBA21 | FBA21-F | TGATACTCCCTCTGTTCATT |
|  | FBA21-R | GAGAGGTAGACGGTGGATAAGAA |
| TaFBA6-  Promoter | FBA6-SP0 | CAGCCAAATAATACTCCTCTGCCTGACAA |
|  | FBA6-SP1 | ACGATGGACTCCAGTCCGGCCCGGACAACCATGGAGACGGTCATC |
|  | FBA6-SP2 | CGGACAACCATGGAGACGGTCATC |
| TaFBA12-  Promoter | FBA12-SP0 | GCAGGAGGAAGAGGACGGATCATAC |
|  | FBA12-SP1 | ACGATGGACTCCAGTCCGGCCACGGAATCGCGAGCATCGGCAGATC |
|  | FBA12-SP2 | CGCCTCGAACGAGCTAGAACCAAACAC |
| Arbitrary degenerate (AD) primer | AC1 | ACGATGGACTCCAGAG |
|  | LAD1 | ACGATGGACTCCAGAGCGGCCGC(G/C/A)N(G/C/A)NNNGGAA |
|  | LAD2 | ACGATGGACTCCAGAGCGGCCGC(G/C/T)N(G/C/T)NNNGGTT |
|  | LAD3 | ACGATGGACTCCAGAGCGGCCGC(G/C/A)(G/C/A)N(G/C/A)NNNCCAA |
|  | LAD4 | ACGATGGACTCCAGAGCGGCCGC(G/C/T)(G/A/T)N(G/C/T)NNNCGGT |
