# Supplementary material for: Molecular Characterization, Gene Evolution, and Expression Analysis of the Fructose-1, 6-bisphosphate Aldolase (FBA) Gene Family in Wheat (Triticum aestivum L.)
Source: Front Plant Sci. 2017 Jun 14;8:1030. doi: 10.3389/fpls.2017.01030 (PMC5470051; doi:10.3389/fpls.2017.01030)
Supplement: Table S3 — FBA genes of wheat and wheat relatives. [file Table3.DOCX]

**Table S3.** *FBA* genes of wheat and wheat relatives

| Organism | Gene ID (cDNA) | Gene name | Subfamily | Gene ID (gDNA) | Gene Length  (bp) |
| --- | --- | --- | --- | --- | --- |
| *Triticum aestivum*  (AABBDD) | [Traes_3AS_2F7B3D3C1](http://plants.ensembl.org/Triticum_aestivum/Gene/Summary?db=core;g=Traes_3AS_2F7B3D3C1;tl=HE3QlynNgbpypv9e-8871704-170102066) | *TaFBA1* | I | [TGACv1_scaffold_210508_3AS](https://urgi.versailles.inra.fr/blast/data/146915739034.blast1.html#CpFBA-3AS:TGACv1_scaffold_210508_3AS) | 3662 |
|  | TRAES3BF050800020CFD_g | *TaFBA2* | I | [TGACv1_scaffold_221301_3B](https://urgi.versailles.inra.fr/blast/data/146915739034.blast1.html#CpFBA-3AS:TGACv1_scaffold_221301_3B) | 3720 |
|  | [Traes_3DS_D42FCD586](http://plants.ensembl.org/Triticum_aestivum/Gene/Summary?db=core;g=Traes_3DS_D42FCD586;tl=HE3QlynNgbpypv9e-8871706-170126847) | *TaFBA3* | I | [TGACv1_scaffold_273209_3DS](https://urgi.versailles.inra.fr/blast/data/146915739034.blast1.html#CpFBA-3AS:TGACv1_scaffold_273209_3DS) | 3975 |
|  | [Traes_4AL_C3729F680](http://plants.ensembl.org/Triticum_aestivum/Gene/Summary?db=core;g=Traes_4AL_C3729F680;tl=HE3QlynNgbpypv9e-8871707-170095382) | *TaFBA4* | I | [TGACv1_scaffold_289694_4AL](https://urgi.versailles.inra.fr/blast/data/146915739034.blast1.html#CpFBA-3AS:TGACv1_scaffold_289694_4AL) | 3180 |
|  | [Traes_4BS_D12DBE6D3](http://plants.ensembl.org/Triticum_aestivum/Gene/Summary?db=core;g=Traes_4BS_D12DBE6D3;tl=HE3QlynNgbpypv9e-8871708-170095567) | *TaFBA5* | I | [TGACv1_scaffold_328216_4BS](https://urgi.versailles.inra.fr/blast/data/146915739034.blast1.html#CpFBA-3AS:TGACv1_scaffold_328216_4BS) | 3058 |
|  | [Traes_4DS_F311D64BE](http://plants.ensembl.org/Triticum_aestivum/Gene/Summary?db=core;g=Traes_4DS_F311D64BE;tl=HE3QlynNgbpypv9e-8871709-170095645) | *TaFBA6* | I | [TGACv1_scaffold_361640_4DS](https://urgi.versailles.inra.fr/blast/data/146915739034.blast1.html#CpFBA-3AS:TGACv1_scaffold_361640_4DS) | 3066 |
|  | predicted | *TaFBA7* | I | [TGACv1_scaffold_392499_5AS](https://urgi.versailles.inra.fr/blast/data/146915739034.blast1.html#CpFBA-3AS:TGACv1_scaffold_392499_5AS) | 1810 |
|  | Traes_5BS_017F8702A | *TaFBA8* | I | [TGACv1_scaffold_423387_5BS](https://urgi.versailles.inra.fr/blast/data/146915739034.blast1.html#CpFBA-3AS:TGACv1_scaffold_423387_5BS) | 1981 |
|  | Traes_5DS_D693664F7 | *TaFBA9* | I | [TGACv1_scaffold_456793_5DS](https://urgi.versailles.inra.fr/blast/data/146915739034.blast1.html#CpFBA-3AS:TGACv1_scaffold_456793_5DS) | 1807 |
|  | [Traes_3AL_441C0AE1B](http://plants.ensembl.org/Triticum_aestivum/Gene/Summary?db=core;g=Traes_3AL_441C0AE1B;tl=DYAdbsLCpFI743lM-8871713-170128866) | *TaFBA10* | I | TGACv1_scaffold_196429_3AL | 2539 |
|  | [Traes_3AL_E9A97403A](http://plants.ensembl.org/Triticum_aestivum/Gene/Summary?db=core;g=Traes_3AL_E9A97403A;tl=DYAdbsLCpFI743lM-8871714-170129048) | *TaFBA11* | I | TGACv1_scaffold_195966_3AL | 2369 |
|  | [TRAES3BF044000030CFD_g](http://plants.ensembl.org/Triticum_aestivum/Gene/Summary?db=core;g=TRAES3BF044000030CFD_g;tl=DYAdbsLCpFI743lM-8871715-170129372) | *TaFBA12* | I | TGACv1_scaffold_221609_3B | 2161 |
|  | [TRAES3BF107400010CFD_g](http://plants.ensembl.org/Triticum_aestivum/Gene/Summary?db=core;g=TRAES3BF107400010CFD_g;tl=DYAdbsLCpFI743lM-8871716-170135985) | *TaFBA13* | I | TGACv1_scaffold_222439_3B | 3378 |
|  | [Traes_3DL_0E549AF9C](http://plants.ensembl.org/Triticum_aestivum/Gene/Summary?db=core;g=Traes_3DL_0E549AF9C;tl=DYAdbsLCpFI743lM-8871717-170129606) | *TaFBA14* | I | TGACv1_scaffold_251713_3DL | 2463 |
|  | Traes_3DL_C5D9E24D5.1 | *TaFBA15* | I | TGACv1_scaffold_250475_3DL | 2629 |
|  | [Traes_3DL_2AAF732B7](http://plants.ensembl.org/Triticum_aestivum/Gene/Summary?db=core;g=Traes_3DL_2AAF732B7;tl=DYAdbsLCpFI743lM-8871719-170129948) | *TaFBA16* | I | TGACv1_scaffold_252211_3DL | 2466 |
|  | TRIAE_CS42_5AS_TGACv1_394081_AA1278040:TRIAE_CS42_5AS_TGACv1_394081_AA1278040.1 cds:_protein_coding | *TaFBA17* | I | gnl\|TA_TGACv1.30.dna.genome\|TGACv1_scaffold_394081_5AS dna:scaffold:1:45711:1 | 996 |
|  | TRIAE_CS42_5BS_TGACv1_423736_AA1382520:  TRIAE_CS42_5BS_TGACv1_423736_AA1382520.1 cds:_protein_coding | *TaFBA18* | I | TGACv1_scaffold_423736_5BS | 7970 |
|  | Traes_7AS_E14E2CD4B.1;  Traes_7AS_7577D92CD.1;  Traes_7AS_3B66B513A.2;  Traes_7AS_75AD1275F.1 | *TaFBA19* | II | TGACv1_scaffold_571185_7AS | 17081 |
|  | Traes_7BS_803489ECB.1 | *TaFBA20* | II | TGACv1_scaffold_594185_7BS | 21180 |
|  | Traes_7DS_A60E1357C.1 | *TaFBA21* | II | TGACv1_scaffold_624510_7DS | 14360 |
| *Triticum monococcum*  (A^m^A^m^) | predicted | *TmFBA1* | I | TGAC_WGS_monococcum_v1_contig_70785;  TGAC_WGS_monococcum_v1_contig_2209488 | 3179 |
|  | predicted | *TmFBA2* | I | TGAC_WGS_monococcum_v1_contig_911932 | 2598 |
|  | predicted | *TmFBA3* | I | TGAC_WGS_monococcum_v1_contig_2206181 | 1271 |
|  | predicted | *TmFBA4* | I | TGAC_WGS_monococcum_v1_contig_913352 | 1997 |
|  | predicted | *TmFBA5* | I | TGAC_WGS_monococcum_v1_contig_80588 | 1944 |
|  | predicted | *TmFBA6* | II | TGAC_WGS_monococcum_v1_contig_2200674  TGAC_WGS_monococcum_v1_contig_905792 | 13541 |
| *Triticum urartu*  ( A^u^A^u^) | gb\|GAKL01024514.1\|:343-1506 TSA: Triticum urartu UCW_Tu-k51_contig_2936 transcribed RNA sequence | *TuFBA1* | I | gi\|457606570\|gb\|AOTI010257369.1\| Triticum urartu cultivar G1812 contig257369 | 3153 |
|  | gb\|GAKL01071595.1\|:1650-2816 TSA: Triticum urartu UCW_Tu-k63_contig_11296_merged transcribed RNA sequence | *TuFBA2* | I | gi\|456165423\|gb\|AOTI011116922.1\| Triticum urartu cultivar G1812 contig1116923 | 2550 |
|  | gb\|GAKL01061013.1\|:486-1643 TSA: Triticum urartu UCW_Tu-k55_contig_17698 transcribed RNA sequence | *TuFBA3* | I | TGAC_WGS_urartu_v1_contig_1368502 | 1271 |
|  | gb\|GAKL01065239.1\|:167-1243 TSA: Triticum urartu UCW_Tu-k63_contig_925 transcribed RNA sequence | *TuFBA4* | I | scaffold47124 dna:supercontig supercontig:ASM34745v1 | 1903 |
|  | gb\|GAKL01061891.1\|:358-1434 TSA: Triticum urartu UCW_Tu-k41_contig_1969 transcribed RNA sequence | *TuFBA5* | I | scaffold41299 dna:supercontig supercontig:ASM34745v1 | 1861 |
|  | TRIUR3_09015:TRIUR3_09015-T1 cds:NOVEL_protein_coding | *TuFBA6* | I | >scaffold100981 dna:supercontig supercontig:ASM34745v1:scaffold100981:44469:48467:1 | 3999 |
|  | TRIUR3_07773 | *TuFBA7* | II | TGACv1_scaffold_571185_7AS dna:scaffold:1:39410:1  scaffold31780 dna:supercontig supercontig:ASM34745v1:scaffold31780:40463:53589:-1  TGAC_WGS_urartu_v1_contig_172733  TGAC_WGS_urartu_v1_contig_165021  TGAC_WGS_urartu_v1_contig_1354629  TGAC_WGS_urartu_v1_contig_187439  TGAC_WGS_urartu_v1_contig_1383218  gi\|457149728\|gb\|AOTI010609382.1\| Triticum urartu cultivar G1812 contig609383, whole genome shotgun sequence  gb\|AOTI010110028.1\|:2892-3596 Triticum urartu cultivar G1812 contig110028, whole genome shotgun sequence  7AS dna:scaffold:1:39410:1  TGAC_WGS_urartu_v1_contig_170979 | 17524 |
| *Aegilops speltoides*  *(SS)* | predicted | *AspFBA1* | I | TGAC_WGS_speltoides_v1_contig_232381 | 3285 |
|  | predicted | *AspFBA2* | I | TGAC_WGS_speltoides_v1_contig_206499 | 2591 |
|  | predicted | *AspFBA3* | I | TGAC_WGS_speltoides_v1_contig_1587160 | 1264 |
|  | predicted | *AspFBA4* | I | TGAC_WGS_speltoides_v1_contig_204306 | 1014 |
|  | predicted | *AspFBA5* | II | TGAC_WGS_speltoides_v1_contig_237339  TGAC_WGS_speltoides_v1_contig_247567  TGAC_WGS_speltoides_v1_contig_325600  TGAC_WGS_speltoides_v1_contig_204480  TGAC_WGS_speltoides_v1_contig_218741  TGAC_WGS_speltoides_v1_contig_493927  TGAC_WGS_speltoides_v1_contig_405515  TGAC_WGS_speltoides_v1_contig_245924 | 13938 |
| *Aegilops sharonensis*  *(S^sh^S^sh^)* | SRA | *AshFBA1* | I | TSL_WGS_sharonensis_v1_contig_1115908;  TSL_WGS_sharonensis_v1_contig_2448421 | 3175 |
|  | SRA | *AshFBA2* | I | TSL_WGS_sharonensis_v1_contig_1087903 | 2596 |
|  | SRA | *AshFBA3* | I | TSL_WGS_sharonensis_v1_contig_223171 | 1273 |
|  | SRA | *AshFBA4* | I | TSL_WGS_sharonensis_v1_contig_114437 | 1895 |
|  | SRA | *AshFBA5* | I | TSL_WGS_sharonensis_v1_contig_89477 | 2002 |
|  | SRA | *AshFBA6* | II | TSL_WGS_sharonensis_v1_contig_2070  TSL_WGS_sharonensis_v1_contig_21402  TSL_WGS_sharonensis_v1_contig_2453827  TSL_WGS_sharonensis_v1_contig_1088569  TSL_WGS_sharonensis_v1_contig_21402 | 14055 |
| *Aegilops tauschii*  *(DD)* | gi\|1033543732:197-1360 TSA: Aegilops tauschii mRNA, contig: PI499262_c31692_g1_i1, strain: PI499262, transcribed RNA sequence | *AtaFBA1* | I | gi\|451137717\|gb\|AOCO010029850.1\| Aegilops tauschii cultivar AL8/78 contig29850 | 3572 |
|  | gi\|1027341591:325-1491 TSA: Aegilops tauschii mRNA, contig: KU2124_c26345_g1_i1, strain: KU-2124, transcribed RNA sequence | *AtaFBA2* | I | gi\|450840298\|gb\|AOCO010325492.1\| Aegilops tauschii cultivar AL8/78 contig325492 | 2543 |
|  | gi\|1033579074:477-1634 TSA: Aegilops tauschii mRNA, contig: PI499262_c5748_g1_i1, strain: PI499262, transcribed RNA sequence | *AtaFBA3* | I | gi\|450387101\|gb\|AOCO010778689.1\| Aegilops tauschii cultivar AL8/78 contig778689 | 1273 |
|  | F775_27796:EMT05696 | *AtaFBA4* | I | Scaffold85892 dna:supercontig supercontig:ASM34733v1 | 1903 |
|  | F775_26377:EMT15181 | *AtaFBA5* | I | Scaffold42352 dna:supercontig supercontig:ASM34733v1 | 1983 |
|  | F775_32635:EMT31985 | *AtaFBA6* | I | Scaffold3341 dna:supercontig supercontig:ASM34733v1 | 2036 |
|  | F775_29158:EMT12443 | *AtaFBA7* | II | TGAC_WGS_tauschii_v1_contig_105742  TGAC_WGS_tauschii_v1_contig_25047 | 13926 |
