# Supplementary material for: Molecular Characterization, Gene Evolution, and Expression Analysis of the Fructose-1, 6-bisphosphate Aldolase (FBA) Gene Family in Wheat (Triticum aestivum L.)
Source: Front Plant Sci. 2017 Jun 14;8:1030. doi: 10.3389/fpls.2017.01030 (PMC5470051; doi:10.3389/fpls.2017.01030)
Supplement: Table S8 — Primers used for qRT–PCR. [file Table8.DOCX]

**Table S8. Primers used for qRT–PCR**

| Gene | Primer | Primer Sequences |
| --- | --- | --- |
| FBA1/2/3 | FBA1/2/3-F | GGACTTGCTCGCTATGCTGCTA |
|  | FBA1/2/3-R | ACCACACCTTCTCTGCCACCT |
| FBA4/5/6 | FBA4/5/6-F | CATCCTCCTGAAGCCAAGCAT |
|  | FBA4/5/6-R | GACTGACCGCCAGACAAGAA |
| FBA7/8/9 | FBA7/8/9-F | GCCGAGACCTTCTACCAGATGG |
|  | FBA7/8/9-R | TGCCCTCCCGACAGGAACAT |
| FBA13/16 | FBA13/16-F | AGTCCTCAAGATCGGCGCCACC |
|  | FBA13/16-R | GCCACCTTCTTGGAGTCGGAAC |
| FBA10/12/18 | FBA10/12/18-F | CTCAGCGGCGTGATCCTGTTTG |
|  | FBA10/12/18-R | GTTGGTTCCAGCAAGCTCAATGGT |
| FBA14/15/17 | FBA14/15/17-F | GTCCCAGCCATTGTCTTCCTCTCT |
|  | FBA14/15/17-R | GCCTTCTCCTCGTTCTCCACCTT |
| FBA11 | FBA11-F | GTCCCAGCCATTGTCTTCCTCTCT |
|  | FBA11-R | GCCTTCTCCTCGTTCTCCACCTT |
| 2FBA | 2FBA-F | GTCACCAGCCGCCAACTCATTA |
|  | 2FBA-R | CCACAAAGGCACACCAGCTAAAG |
| ADP-RF | ADP-RF-F | GCTCTCCAACAACATTGCCAAC |
|  | ADP-RF-R | GCTTCTGCCTGTCACATACGC |
| Actin2 | Actin-R | CTCCATGTCATCCCAGTTG |
|  | Actin-F | CACTGGAATGGTCAAGGCTG |
