# Supplementary material for: Molecular Characterization, Gene Evolution, and Expression Analysis of the Fructose-1, 6-bisphosphate Aldolase (FBA) Gene Family in Wheat (Triticum aestivum L.)
Source: Front Plant Sci. 2017 Jun 14;8:1030. doi: 10.3389/fpls.2017.01030 (PMC5470051; doi:10.3389/fpls.2017.01030)
Supplement: DOC1 — Promoter sequences of TaFBA genes. [file DataSheet1.zip › Data sheet/DOC1-Promoter sequences of TaFBA genes.docx]

gene name promoter UTR CDS

>TaFBA1

GGGTCAATCACCTCCCACTAGTGGGCCGGCCCAATAACACACTTTCATGCTGTTTTCGGAAGCTTCTATGGACTGGTTTTAGAAGCTTCCATGTGGTTTTTATTTCTGTGTACTTCCCCTATCATTTTTTTCACATTCTTTTACTTTTTTTGTGTGTTTTTTCTCTTGGTTTTCTTCATCTTTCGAATAGATGTCGATTTTTTTCAAATGCATGTCGAACATTTTTTGTATATAGGTTGAACATTTTTTGATACACATTAAACACTTTTTAAAGACATCATAAACCATTATATAGGTTGAACATTTTTTGAACATTGAACATTTTTTAAGACATAGTGAACATTAACACATGTTGCACATTGTTTTTAAATTTCATGAACGTTTTCTGCAAAACATGTGAACGGTTGTTAAATGTTTAGAACCAAATTATTAAATGACACGATCATTTTTATATGTTATATATTTTTTTAATTATGTGAACATTTTTGTTAGTAATTTACATTGCAAATTAAGTGGACTTCAATTATTACTCGATTGTGTCCGTGCTATATATTCTCTACATGGGAAATTTAACATGCATTATGTATATATAACTTTAGCAACATATTTCAAAAGCCCGTGACAACGCACGTGCATTCTATTAGAAACAACAGTATCCGGGGCATGCCTAAGGGAGGTACTAACCACATCAGACATCACCAGTCACCAATCAATATATCAGGGACAAGCCAAAGGAAGTTATTTAAATTATAACACAATAGCATGTAATTTTAAAAGTGTCCAGATATTTAAATAAATATTAACATAACTTCTATAAAATGGTTATGTGTATTAAAATAAATTTTCCATGTATTTGATAAAGTAGATGTATTATATAAGAGTGTTCATGCTTTAATAAAATGGTTATGTTGTTTTAAAAAGCGTTCATGTGTTTTGCACACACAAAAATAGGAGGTATGGAAAAATATATACAAGCCTAGTGCGTGCCTTGAGATTTTAGATCCTCAAATGAATACATGAATATTTTATAAAAAAAATAATGTTTTGAAAATTATATGAACATCTATTGAAATTACAAACATTTAAAAAATACATCAATATTCTTATTAAATGTGAACACTTTTTAAAACTCCACAAACATTTTGAAAATTCTAAAAAAGAACCAACACGTTTTTTTTCTATATTATAATCTAAAAAAATAACACAAACTTTTTTTTTCTTTGTAATGTGTGCTGACAGATGGGTCCCAGCGGTAAGCCCTGTCGTCGGCATCTCTTCTGGCCTCGGCGGATCCGCCCTGCGACGGGCGGAGAGATCCACGGCCAATATGAAAAGGAAACTATTTCACTCTGCCCCCTCACCCATCGCATATCACTCCAGCCACCTCGGCCCATCGAGAAGAGGGTTGGTCAGGGGCTCCAGCGAAAATACATAGCGAATCGAGGGCCAGGATGCATAAAACCGCGGTCGTACTTCCTCCGCCAGCCACCAACCCACTCCGCCCCTCCCTATTTCTCGCCGTCGCGTCCGAACACAGAGCGCATCGACCGATCGAACCAATGGCGATGGTGACCGCGAAGCTCAGCTCCCCGGCCACCGCCCGGCTCGCCCCCGTCCGGTCCGCGCAGCCCGCCCGCCGCGCCTCGCTCGTCCGCGTCCGCGCCTCCGGCGGCTCCTACGCCGACGAGCTCGTCTCCACCGCGGTGAGCGCCCCCCGCCGCCTCCTCCCTCTGATCCCCGTAGCTTCGCCTAGGTCTAGGAGCCGGTGGCCCGCGGGAGGATCTCGCCCTTGGGGCGTTGCGCGCGTCGTGTGCCGCGATCTGTCCTGGTGGCTCCGGGTTTGTTTGGTTGGCGTCTAGCGATCCGTGACGGGGACGGGTGTTGTGGGGTTGGAGAAAATGAGAGCGTCCGTGCGTTTCTCCGTCGAATTAAATCTCGATTGGAAACGACCTCGTCTCGATGTATTTTTCCAATGTATTTTTCCGTTGCAATCGAATTTGGGCTGATAGATGAGCGCCTATGCTGTGTTCAAGCCTCTGTTAGTTTAGATGGCAGGTTGCGTGATATGGGAAAACCAGTGATAGATTGATTGGCCCAGTTACTCTGTTTTGTCTGCTACTTGCATCGTCCCAGATCAAGCCAGGGTAAATCTGGCCCACTTTTTAGGTTGACTTTTAAATTAAGGTTGAACAACGAGCTTGTTGAGTCTCAGGATGATTGTCCCGGTAGATACGGAAGGAGGTCATTGCGATGCTAGTGTTGCTCTGAAAAGTAAATTCTCAAATCCAAATATCATAGAATTGTTTCTAGGAGATGTGGCCAACTGCCCATCCATATGTTCATATTCATTTGGTTTGTAGATTGTTGGGTGGACTTGCTGACGTATGGTTGCTCTGTGATGGTTTCCTGTGTGGGAAAACCTACCAGCAGTACTTTAAGTCTTGGTTTTGTTAGAATTATTCTTGGCCTTTCTGCTCGGCTGAAAAGGATTTGTCTGCTTTCACGCCATCTCCTTTGCATCGTGTGTACATTAGGTTTTATTTTGTTCCAACTGTAATCTTCTGTTCAAGTGTTGATTTGTCAATTAGTGTGAAGCATGCCCTCATGTAAATGAACCACCGCAAGTCTAGAACTAACACTGATGCTCGTCTTAATTGCTTGATGTTGGGTATGCCAAACTAGTCTTATGCTATTTCCTGTTTATTTTTCTTCAGCGTTCCTTCCTCGCAGCTGCAATGCATTCTCTGTTGTGTTTCATGACTGCAAATCTAATAGTGCGCCCCATTGCAGAAAACTGTTGCTTCCCCTGGCCGTGGGATCCTTGCGATCGACGAGTCGAGTGCAACATGTGGAAAGAGATTGGCATCCATTGGTTTGGACAACACGGAAGTTAACCGCCAGGCTTACAGGCAGCTTTTGCTGACCACTGCTGGTCTTGGTGAATATATCTCTGGTGCTATTCTTTTTGAGGAAACTCTCTATCAGTCCACTACAGATGGCAAGACCTTTGTTGACGTCTTGAAGGACCAGAATATCATGCCTGGTATCAAGGTTGACAAGGTATGCATGCCATGTTCTGAAAATCGTCGATTCGTCGTGCCGTTTGTAGTCACCAAGACAGTCCTAGCATTCTGATTTTAGTACTTGATTTATTTTGTTCTCCCAATAGGGTTTGGTTCCATTGCCCGGATCCAACAATGAATCCTGGTGCCAAGGTCTTGATGGTTTGGCCTCAAGGTGCGCTGAGTACTACAAGCAGGGTGCACGCTTCGCAAAGTGGTCAGTTCATACTCTCCTTGTGCATTTCCTGACTAGTTTTATTCTCTTTCCCTTTCTAAACTTGCTATGCTATATTGATCTTCTACAGGCGAACTGTTGTTAGCATCCCCTGTGGTCCTACTGCATTAGCTGTCAAGGAAGCGGCATGGGGACTTGCTCGCTATGCTGCTATTGCTCAGGTAATACATGTTCTCAACTGCTGAACATTTTGGAATGAAGAAGTGATCCTTTATTTAGTTTGATATTTTACTCATGGAGTTGATGAACAGCATAATCATTTTTATGCTCATATTTGATATCTTATTCCTAGAAACCTGTGCGACGACATAACCTATCCTACAGTATAATCATCCATTGAACACTATCCGATAGCAAGTAGGGAGAATTAATTAACAAGTAGAAAAAGTAAATATCTGTATGCTCATGTAGATATGCTGTTATTGAAATCATATATACCAATGAACGCTAGCATGGTGGTTCTGTTCTGACACCACCAATCGGAACATTTTTCCCTTTGAAATTTCCAACTCAGGTTCTTCTTTGTAAGCTGATACGTACTAGAATTTGATGAAATGAATGCAACAATAGTACTCCCTCTGTTCGCAAATATAAGATGTTCTAACTTTTTTCTGAATCGGATGTATATAGACACATTTTAGTGTATGTTGAAATATTTATTCCATATTGTAATATTCAAAACATCTTATATTTGTGAATGGAGGTAGTACATTATATGATTCAAGAAAATTTATGCATCCAAATACACTATAATCTCGACGTACTATTTTGGGCCCCTCTTTATCTTTACTCATCTTGTTTCAAACCTGAATAATCTGCAGGACAATGGTTTAGTGCCAATTGTGGAGCCAGAGATCCTTCTTGACGGTGACCATGGCATTGAGAGAACTCTCGAGGTGGCAGAGAAGGTGTGGTCAGAGGTGTTCTTCTACCTGGCCGAAAACAACGTTCTGTTTGAGGGCATCCTGCTGAAGCCCAGCATGGTTACCCCTGGTGCTGAGCACAAGGAGAAGGCTTCTCCAGAAGCCATTGCGAAGAACACCCTCACAATGCTGAGGAGGAGAGTACCACCTGCTGTCCCTGGAATCATGGTATTGTCTTCAACCTTGAACCATCTTAGCTTCTGCTGATTATCTTTCTTCAGTCCATTTCCCGTGGACATACATAATAAACAGAGTAAGAAATAGTAGCCATAACCCCGACCGTCCCAATTCCGCAGTTCCTCTCCGGCGGACAGTCCGAGCTGGAGGCAACCATGAACCTGAACGCGATGAACCAGTCCGCCAACCCGTGGCACGTGTCCTTCTCCTACGCCCGGGCACTCCAGAACTCGGTGCTGAAGACGTGGGAGGGGCAGCCCGAGAACATCGAGGCGGCGCAGAAGGCCCTGCTGGTCCGCGCCAAGGCCAACTCCTTGGCGCAGCTCGGCAGCTACACTGGCGAGGGTGAGAGCGACGAGGCCAAGAAGGGCATGTTCCAGAAGGGATACACCTACTGATGAGGCGACGGCAGGATGATTTATATATGAGATGCACGTCATTTTGCGGGGTTTTGTTTTCTAATTATTGGTGTGAATAATCAATCACCAGCGTGTTGGTGCCAACGACTGTTAGCTGTATTGGCATTGCAAGAAACTCTAGGGTTGCCTGAAGTTATATTTATGAGAACAAACAATTACTGTATGCATACCTACCCTGAGAAAGTTGATGATAGCTCACTGTGGGCGGATACCGAGAACAGCTTGGGTTGTGGACCTGAACATCGGGTTTCTACCGACTACAAGTTTCTGAAATTTATGGAAAGTTCACATAGCATAGGTGCAAAAAATATGTACAATTTCTTGAAAAATCAATTCAAAATTTAGC

>TaFBA2

CCAGGTCTTGAAACCGTGACCTCAAGGTCACAAGGCAACAACTTCACTAGGGCGAGCAAATTTGTTTTTTATTAAAATTTGGTACAATTTTGAAAATCTGAACTTTATAAAATTTATTTTTTTGGAATTTTCATTTTTTGAAATTTAAACAAGTTTTGAACATTTTTGAAATTTCTGATTTTTTAAAAATCTACACAAATTTTGAAAATCTGAACATTTTTTGAGTATTTAAACAAATTTTAGAATTTTGAAAATTTATACAAAATTTGAATTTTTGAACAGTTTCTCAAAGATGTCATTGCCCGTGAATGCTGACCAGCGAACGACTGCTGGGGTGTAATCCCTTCGTGCATGCATGCTACGAGGGAATTTGAGCTAAGCTCATGCAGGCTACCAAACACCCCAAAGTGGGTTGAGTAGGGAACTTTGCCCTCATACACTCTTCATGCACCCTATCGAACACACCCTAGCATCCAAGTAGAACTTGCCACTCCACCCACATGCGACTTTTTTTAAAGATAACTAGGTTTAATGTCAATGGAAGATAACTAGCCTTAATGTCCTCTAACTGAAACAGTTTGTGAGAATTAAATGGGCTGAACCTAGTGCGACCAATTTTCACATCTTAGTTTTTTTTTTAATTTATAGAAGTTACTTAAATTATAACAGCAATATCCCGGACATGCCTAAGGGAGGTTCTAACCACATCACCCGTCACCAATCAATGTCAGGGACATGCCTAAAGAAGTTATTTAAAGTATAACACAATACCATGCAATTTTAAAAAAGTCTTCACATATTTAAATAAATATTAACATAACTTCTATAAAATGGTTATGTATGTTAAAATAAAATTTCCATGTATTTGATAAAGTAGATGTATTATATAAGAGTGTTCATGCTTTAATAAAATGGTTGTCTTGTTTTTCAAAGCGTTCACGTGTTTTACACAAAAATAAACAAATAAATAAAGGAGGTATGGAAAAGTAAATACAAGCCTAGTGTGCCTTGAGATTCTACATCCTCAAATGAATACATGGATATTTCATAAAAAGATAATGTTTTGATAGTTAAATGAGCATCTATTTAAATTACAAATATTTTAAAGCTACACCAATATTTTTGTTAAATGTGAACACTTTGTAAAATTCTACAAACATCTTGAAAATTCTAAAAAAGAACCAACATGTTTTTTCTTTAATATTAAAAAAAGAACTAATGTGAAAATGGGCCGTAGTGGAGTGGCCTAGCTTGGTTATGCTACTACCTTGTTCCCGGACATTAATTAGGAGGAGGAGGTTGTGTGTTCGGTTTAGTTTTTCGATTTCATTTTTTCTTTTACCTGCCGTGCGCTGGCAGATGGGTCCCAGCGGTAAGCCATGTCGTAGGCATCTCGGCCGGCCTCGGCGGCGGGCGGAGAGATCCACGGCCAATATAAAAAAGGGAACTATTTCACTCTGGCCCCTCCCCTATCGCCGTATCACGCCAGCAACCTTGACCCCTCGAGAGGAGGAGGGTTGGTCAGGGGCTCGGGCGAACATAGAGGGGAAATGGAGGGCCAGGATGCATAAAACCGCGGTCGTACTTCCTCCGCCAGCCAGCCACCAACCATCCCTATTTCTCGCCGTCGCGTCCAAACGCAGAACGCATCGACCGATCGAACCAATGGCGATGGTGACCGCGAAGCTCAGCTCCCCGGCCGCCGCCCGGCTCGCGCCCTCCGCCGGGAGCGCCCGCCGCGCCTCGCGCGTCCGCGTTCGCGCCTCCGGCGGCTCCTACGCCGACGAGCTCGTCTCCACCGCGGTGAGCGCCCCCCCTCTCTGATCCTCGTAGCTCCGCCTAGGTCTAGGAGCCGGTGGCCCGCGGGAGGATCTCGCCCTTGGGCCGTTGCGCGCGTCGTGTGCCGCGATCTGTCTGCCGTGGCGGCTCCGTTTGTTTGTCTGCTTGGCGTCTAGCGATCCGTGATGGGGATTGCTCTGTGGGGTTGGGGAAAATTGGGGCGTCGCGTGCGTTTTTTTTTCCAGCGAATTCAATCCGGATTGGAACGAACCTTTTTCTGCATGCATCAGGAGCGAAGAGGTCACAATCTCCTCCGTGCAAATCGAATTTGGGCCGCTAGATGTTCAGGAAAGTTGGTGTATATATGCCGTGTCCATGGATTTCTCCTGTTATTTTCGGTAGGGACGGCAGGTTTCATGATCTCAGTCACTCTGTTGCGTCTGCTACTTGCGTCGGCCCCAGATCAGGCCATGGTAAATCTGGTGGTGCATTTCATGTGATGCTGTCGTTGCTCTGAAGATAAACAAACTCTCACTTCCAAATACCATCGAATTGTTTCTAGGCAGTGTGGCCATCTGTTGATAACTAGTAGCTTAATGGGTCGATCTTCTGACATATGACTGCTCTGCAATAATTGTCTGTGTGGAAAGACTACTGGCAGTAGCAGGTTTAAGTTATGGTTTTGTTAGGATTTAATCTTGGCTCTTCAGCTTTGATGAAAAGGATTTGCTTGCTTTCCCATTGGCATGATGGGTGGACTTGCTGACGTACGATTGCTCTGTAATAATTGCCTGTGCTTGGAACGTACTAGCAGCATTTTAAGTTCTTGTTTTTGTTACAATTAATCTTGGCTTTTCCACTTCGATGAAAACGATTTGCTTGCTTTCGCGCCATCTGCTCTGCATCATGCGTATATTAGATTTTATTTTGTTTCAACTTTAATGTTCTGTTGAACTGTTGATTTGTCAATTAGTGTGAAGCATGCCCTGATGCAAATGAACCGTGCCTTTAGATTTTGAACCACCACAAGTCTAAAACCAACACTGATGCTCGTATTAATTGCTTGATGTTGGGTATGCCAAACTGGTCTTAAGCTATTTCCTGTTTATTTTTCTTCAGTGTTCCTTCCTTCACAGCTGCAGTGCATTCTCTGTTGTGTTTCATGACTGCAAATCTAATAGTTCGCCCCATTGCAGAAAACTGTTGCTTCCCCTGGCCGTGGGATCCTTGCGATCGACGAGTCGAGTGCAACATGTGGAAAGAGATTGGCATCCATTGGGTTGGACAACACCGAAGTTAACCGCCAGGCTTACAGGCAGCTGTTGCTGACCACTGCTGGTCTTGGTGAATATATCTCTGGTGCTATTCTCTTTGAGGAAACTCTCTACCAGTCCACTACAGATGGCAAGACCTTTGTTGATGTCTTGAAGGACCAGAATATCATGCCTGGTATCAAGGTTGACAAGGTATGGATGCCATGATCTGACAACCATTGATTCATCGTGCTGTTGCAGTCAGCAAGTCAGCGCTAGCATTCTAATCTTTATTACTTGATTTATTTTGTTCTGCCCAAAAGGGTTTGGTTCCATTGCCCGGATCCAACAATGAATCCTGGTGCCAAGGTCTTGATGGTTTGGCCTCAAGGTGTGCTGAGTACTACAAGCAGGGTGCACGCTTCGCAAAGTGGTCAGTTCATACCCTCCTTGCGCATTTTATGTATTGTTTTGTTCTCTTGCCCCTTCTAAACTTGCTATGCTATATTGATCTTCTGCAGGCGGACTGTTGTTAGCATCCCTTGTGGTCCTACTGCATTAGCTGTCAAGGAAGCGGCATGGGGACTTGCTCGCTATGCTGCTATTGCTCAGGTCACTATACATGCTGTCAATCAACTACTGAACGTTTTGCAATGAAGAAGTTATCCTTTATTTAGTTTGATATTTTATTCATGGAGAGTTGATGAACAGCATAGTCATTTTTATGCTCGTATTTAATATCTTGTACCTGGAAAACTGTGTGGCGATATAACCTATCATACAGTATATTCATCCATTGAACACTATCTGATAGTAAGTAGGGAGAACTAATTAACAAGTGAAAAAAGTATATGATGCTCATGTAGGTACGTTGTTATAATCGCTACTATTTTGGGCTCCTCTTTATCTGTACTCAGCTTGTTTCAACCTGAACAATCTGCAGGACAATGGTTTAGTGCCAATTGTGGAGCCAGAGATCCTCCTCGACGGTGACCATGGCATCGAGAGAACTCTTGAGGTCGCCGAGAAGGTGTGGTCCGAGGTGTTCTTCTACCTGGCCGAAAACAATGTTCTTTTTGAGGGCATCCTGCTGAAGCCCAGCATGGTTACCCCTGGTGCTGAGCACAAGGAGAAGGCTTCTCCAGAAGCCATTGCGAAGAACACCCTCACAATGCTGAGGAGGAGAGTACCGCCCGCTGTCCCTGGAATCATGGTATTGCCTTCAACCTCGAACCATTTAGCTTCGGCTGATTATTTTTCTCCAGTCCATTTCCGGTGGACATATACATAATAAACAGAGTAAGAAACAGTAGCCATAACCCCGATCGCCCCTTTCCCTTCAGTTCCTTTCTGGCGGGCAGTCCGAACTGGAGGCGACGATGAACCTGAACGCGATGAACCAGTCCGCCAACCCGTGGCACGTGTCCTTCTCGTACGCCCGGGCCCTCCAGAACTCGGTGCTGAAGACATGGCAGGGGCAGCCCGAGAACATCGAGGCGGCGCAGAAGGCCCTGCTGGTCCGCGCCAAGGCCAACTCGTTGGCGCAGCTCGGCAGCTACACGGGCGAGGGCGAGAGCGACGAGGCCAAGAAGGGCATGTTCCAGAAGGGCTACACCTACTGATGAGGCGGCGGCAGGATGATTTACATATGAGACGCACGTCATTTTGCGGGGTTTTGTTTTTTTAACTGTGGGTGTGAATAATCAATCACCAGCGTGTTGGTGCCAACGACTGTTAGCTGTATTGGCAGCGCGGAAATTCTAGGGTTGCCTGAAGTTATATTTATATGAGAACAAACAATTTACTGTATGCATGCCTACCCAGAGAATGTTGATGGGCTGTGGGCCGATGCCGGGAACAGCTTGGGTTGTGGACCTGAGAAGCCCTGGCGCACACACGGAGAATTTACCCGGGGCATCTGGTTTCTACAGACTAGTACTCCTACTAAGAGTGTTTCTAAAGTAGTGATCTAAATGCTCTTATATTTCTTTACAGAGGGAGTACAACTTTCTTAAAAGATCAATTCAAAATTCGGCCTATACCCCTAGAACAAGAATAGTGATGATAATGGGCCAAATCAACAGTCCCAACTTTACTAGTATGCATTTTTTTTCCTGTGTTGCGTTTTTGCGAAGTAGTACATGTATATTGCATATGTTGTCACAAATTATCAGACATTTTAAGGTGC

>TaFBA3

GAAATACAGAATGCACACTGAACATTGAACATTTTTCAAGACCTAGTGAACATTTCTAAACACATGTTGCACATTGTTTTTAAATTTCATGAACGTTTTCTGCAAAACATGTGAACGGTTGTTAAATGTTTAGGACCAAATTATTAAATGACACAATCATTTTTATATGTTATGAACATTTTTTGAACTTATGTGAACATTTTTGTTATTAATTTACATTACAAGTTAAGTGGACTTCAATTATTACTCGATTGTGTCCGTGCTATCTATTCTCTACATGGGAAATTTAACATGCATTATGTATATATAACTTCAGCAGCATATTTCAAAAGCCCGTGACAACGCACGTGCATTCTATTAGAAACAACAGTATCCGGGCATGCCTAAGGGAGGTACTAACCACATCAGACATCACCAGTCACCAATCAATATATCAGGGACAAGCCAAAGGAAGTTATTAAATTATAACACAATACCATGTAATTTTAAAAGTGTCCAGATATTTAAATAAATATTAACATAACTTCTATAAAATGGTTATGTGTATTAAATAAATTTTCCATGTATTTGATAAAGTAGATGTATTATATAAGAGTGTTCATGCTTTAGTAAAATGGTTATGTTGTTTTAAAAAGCGTTCATCTGTTTTACACAAAAAATAAATAAAAAAATAGGAGGTATGGAAAAATACATACAAGCCTAGTGTGTGCCTTGAGATTTTACATCCTCAAATGAATACATGAATATTTATAAACAATATTTTTTAGAAATATATTTAATTATAACATTTAAACTACACTAATATTTTTATTAAAATGTGAACACTTTTTAAAATTCTAAAAGGTAACCAACATGTTTCTTTTCTTTAATCTAAAAAAAGAACTATGATGTGAAAATGGGCCACAGTGGAGTGGCTAGCTCGGCTATGCCACCTTGTTAGGAGACATTAGGAGGAGGTATTTTTTCTTACGGGGTGACATCAGAAGGAGGGTGCGTGTTCGGGTTTGTTTAGTTTTTTGATTCCGTTTTTCATTTGTGCTGTGTGCTGACAGATGTGTCCTGACGGTAAGCTTTGTCTTGCAAATGCAAAAACAATAAAGTATGAGTTTCCTTTATATTTTTGCGAAAAGCGTTGCGACCGCGCGGGTATGGCCGCCGATAGTGGCCCCATGTCGCGCTTCACGCCTCATCAAAGTCTTGGGCATACACACAAACGAGACACTAAAGTGAACATCGACGACAACTTCAAGCACCACTGGTGCAATTACCTCCATATTTGAAGGAATTCCATCAGCCTAATATATACAATCTGGTGAATGGAGGTAGTAGGAAGAGAAGGGCGTTGTCGTGAGTCCGGGAGAAAGGAGGGCGTTCTCGTGATTCCAACGTGGGCATCTCATCTGGCCTCGGCGGATCCGCCCAGCGGCGGAGAGATCCACGGCCAACATAAAAAAACGAAACTATCTCACTCTGGCCCCTCACCTATTGCCGTATCACGCCAGCCACCTCGGTCCGTCGAGAAGAGGGTTGGTCAGGGGCTCCGTTGAACATACTTAGCAAATGGAGGGCCAGGATGCATAAAACCGCGGTCGTACTTCCTCCGCCAGCCACCAACCCACTCCCTCTCCCTATTTCTCGCCGTCGCGTCCAAACGCAGAACGCATCGACCGATCGAACCAATGGCGATGGTGACGGCGAAGCTCAGCTCCCCGGCCGCCGCCCGGCTCGCCCCCGTCCGGTCCGCGCAGCCCGCCCGCCGCGCCTCGCTCGTCCGCGTCCGCGCCTCCGGCGGCTCCTACGCCGACGAGCTCGTCTCCACCGCGGTGAGCGCCCCCGCCGACCAACCCTACGCCTCCCCCCCTCTCCGACCCCCGTAGCCTCGCCTAGGTCTAGGAGCCGGTGGCCCGCGGGAGGATCTCGCCTTGGGGCGTTGCATGCGGCGATCTGTGGTGGTGGCTCTGTTTGTTTGTTTAGTTTGGGTTGGCGTCTAGCGATCCGTGATGGGGACTGCTGTGTGGGGTTGGGGAAAACGGGGCCGTCGCGTGTGATTTTCCCAGCGAATTAAATTTCGATCGGGAACGAACCTTTTCTGCATGCATCAGGAGCGAGGAGGTCACAATCTCTTCCGTGCAATCGAATTTGGGCCGCTAGATGTGTGTATATATGCCGTGTCCATGGATTTCTTCTGTTATTTTAGATAGGGATAGCAGGTTTCACGATCTCAGTTACTCTGTTGCGTCTGGTACTTGCATCGGTCCCAGATCAGGCCACGGTAAATCTGGCGGTACATTTGGCTGTCTTTGTTAGGGTTGACTTGTAAATTGAGTCGCAAGACGATTGTCCCAGTAGCTATGGAAGTTGGCCATGTGATGCTGGTGTTGCTCTGAAGATAAACTCTCGCTTCCAAATACCATCGAATTGTTTCTAGGCAGTGTGGCCTTCTGTTACTAGTAGCTTGATGGGTTGATCTTCTGACGTACGATTTCTCTGCAATAATTGCCTGCGCGGAAAGCCTACTAGCAGTAGCAGTTTTAAGTTATGGTTTTGTTAGGATTTAATCTTGGCTCTTCAGCTTCGGTGAAAAGGATTTGCTTGTTTTCACGTTGGCATGATGGGTGGACTTGCTGACGTACGATTGTTCTGTAATAATTGCCTGTGCTTAAAACCAACTAGCAGCATTTTAAGTTCTTGTTTTTGTTAGAATTAATCTTGGCTTTTCCGCTTCGATGAAAACGATTTGCTTGCTTTCACGCTATCTGCTCTGCGTCATGTGTATATTAGATTTTATTTTGTTTCAACTGTAATGTTCTGTTGAACTGTTGATTTGCCAATTAGTGTGAAGCATGCCCTCATGCAAATGAACCATGCCTTTAGGTTTTGAACCACCGCAAGTCTAAAACTAACACTGATGATCGTATTAATTGTTTGATGTTGGGTATGCCAAACTGGTCTTGTAAGCTATTTCCTGTTTATTTTTCTTCAGTGTTCCTTCCTTCACAGCTGCAATGCATTCTCTGTTATGTTTCATGACTGCAAATCTAATAGTTCGTCCCATTGCAGAAAACTGTTGCTTCCCCTGGCCGTGGGATCCTTGCGATCGACGAGTCGAGTGCAACATGTGGAAAGAGATTGGCATCCATTGGTTTGGACAACACGGAAGTTAACCGCCAGGCTTACAGGCAGCTGTTGCTGACCACTGCTGGTCTTGGTGAATATATCTCTGGTGCTATTCTTTTTGAGGAAACTCTCTACCAGTCCACTACAGATGGCAAGACCTTTGTGGACGTCTTGAAGGACCAGAATATCATGCCTGGTATCAAGGTTGACAAGGTATGCATGCCATGATCTGACAACCGTTGATTTATCGTGATGTTGTAGTCAGCAAGACAGCTCTAGCATTCTAATCTTATTACTTGATTTATTTTTTTCTGCCCAAAAGGGTTTGGTTCCATTGCCCGGATCCAACAATGAATCCTGGTGCCAAGGTCTTGATGGTTTGGCCTCAAGGTGTGCTGAGTACTACAAGCAGGGTGCACGCTTCGCGAAGTGGTCAGTTCATACCCTTGTGCATTTTCTGACTTGTTTTATTCTCCTGTCCTTTCTAAACTTGCTATGCTATATTGATCTTCTATAGGCGGACTGTTGTTAGCATCCCTTGTGGTCCTACTGCATTAGCTGTCAAGGAAGCGGCATGGGGACTTGCTCGCTATGCTGCTATTGCTCAGGTAATACATGCTCTCAACTGCTGAACATTTTGGAATGAAGAAGTGATCCTTTATTTAGTTTGATATTTTACTCATGGAGAGTTGATGAACAGCATAATCATTTTTATGCTCATATTTGATATCTTATTCCTAGAAACCTGTGCGATGACATAACCTATCCTACAGTATAATCATCCATTGAACACTATCTCATAGCAAGTAGGGAGAACTAATTAATACGTAGAAAAAGTAAATATCTGTATGCTTGTGTAGGTATGCTGTTATTGAAATCATATATACCAATGAACACTAGCATGGTGGTTCTGTTCTGACACCACCAATCGGAACATTTTTCCCTTTGAAATTTCCAAATCAGGTTCTTCTTCGTAAGCTGATACTAGAATTTGATGAAATGAATGCAAAAATAGTACTCCCTCTGTTCGCAAACATAAGATGTTCTAACTTTTTTCTGAATCGGATGTATATAGACACGTTTTAGTGTATGGTGAAAGATTTAGTCCATATCTCAGTCCGTATGTAGTCCAATTGAAATACCCAAAACATCTTATATTTGTGAATGGAGGTAGTGTACATTATATGATTCAATAAAATTTATGCAACCTACACTATAATCTCTACTATTTTGGGCCCCTCTTTATCTTTACTTATCTTGTTTCGAACTTGAATAATCTGCAGGACAATGGTTTAGTGCCAATTGTGGAGCCAGAGATCCTTCTGGACGGTGACCATGGCATCGAGAGAACTCTTGAGGTGGCAGAGAAGGTGTGGTCAGAGGTGTTCTTCTACCTGGCCGAAAACAATGTTCTGTTTGAGGGCATCCTGCTGAAACCCAGCATGGTTACCCCTGGTGCTGAGCACAAGGAGAAGGCTTCTCCAGAAGCCATTGCGAAGAACACCCTCACAATGCTGAGGAGGAGAGTACCGCCCGCGGTCCCTGGAATCATGGTATGGTCTTCAACCTCTGAACCATTTTAGCTTCTGCTGATTATTTTTCTTCAGTCCATTTCCGGTGGACATATACTCCCTCCGTTCCTAAATATAAGTCTTTGTAGACATTTCAAATGGAATATAATATACGGATGCATGTAGACATATTTTAGTGTGTAGATTCACTCATTTTGCTCCGTATGTAGTCACCTGTTGAAATCTCTAGAAAGACTTATATTTGGGAACGGAGGGAGTACATAATAAACAGAGTAAGAAACAGTAGCAATAACCCCGATCGTCCCTTTCCCTTCAGTTCCTTTCCGGCGGACAGTCCGAGCTGGAGGCAACCATGAACCTGAACGCGATGAACCAGTCCGCCAACCCGTGGCACGTGTCCTTCTCGTACGCCCGGGCCCTCCAGAACTCGGTGCTGAAGACGTGGCAGGGGCAGCCCGAGAACATCGAGGCGGCGCAGAAGGCCCTGCTGGTCCGCGCCAAGGCCAACTCGTTGGCGCAGCTCGGCAGCTACACCGGCGAGGGCGAGAGCGACGAGGCCAAGAAGGGCATGTTCCAGAAGGGCTACACCTACTGATGAGGCGGCGGCAGGATGATTTATATATGAGATGCACGTCATTTTGCGGGGTTTTGTTTTCTAATTATGGGTGTGAATAATCAATCACCAGCGTGTTGGTGCCAACGACTGTTAGCTGTATTGGCAGTGCGGAAACTCTAGAGTTGCCTGAAGTTATATTTATGAGAACAAACAATTACTGTATGCATACCTACCCTGAGAAAGTTGATGATAGCTCACTGTTCAAAATGG

>TaFBA4

TCCAATAAAAAGACCACTCTGCCATTCAAACTAAATTAGATCTCAGTATCAACTCCGTTCAATTAATACTGAGAATGCCATAATATATGGTCATCATGACCAGGATGCTTGATTTTAAATTTCTGCCGGTTGAGAATTAAGACAAGTCAAGAGTTTGGTCAACCCATGGCATCTCTGATTAATATGCAGAACATATGTTCATCTGAAGCACATAATGAATTTGGCTGAATGATAGGCTGCTGCTCGTGATTCATTACAAGTTAGCTTCAAATGTGATGAATTGAAATGCTGGCAGCAAAGGAACTACTCTCTGCACAAACTCATCATCTGCTTCAACTGATGAAAGAAACCTCATGCCCCTTTTGACAATTCTGAGATAAATACACAATATAAGGATTGAATGCTGTGATCAGAGTTCAGACCTGGTGGCATGTCCGCATGTGCCGCACGACATCATGCACATCAAGCTTGTGATAGCGAGTGTTTTTGTTCAGGCATGCTAATACACTTTATGGGAGAAGTCACATACACCATAGGCAGTCTCTATGGATGTATTTCTCCCGAGTTATAGGACATCATCTGATCTAAGTAGGCCAACCTTGTGTACTTGTAAACATTTTGTAATTACCGGCTTTTGTTATTCAGTAGTGAAATCCCTTTCATCGTATTTTGTGTCTTGTCCTTTTATTGAGGGCACTAGGTGCCACATACCAACTCGCTCTCTGATGTTAATGTTTATGGTGAGTAGACAAAGGTGCCAAATTCCTTTTCTGCTTTACACTTTAAACTACACCTCCAGTCTGGAAAATATGCATATTTGTATCCATATTTCTCTGTTTTTCTCCTAGCCAGTGTGACACTTCCCAGTTCCTACTAATGCATTTTGATAGGAAATACTATGACATCAAAATTCAGACATTTTGGTAAGGCTAGGGAAATCATTTTTTCCTAAATGAAAGCATCATATCAAGATTCATGGGGTCAAGGGATGATACAAGATTGTTACAAGGCACTGGGGCACAAGGAGAAGCAGGACTCTGTCGCAGAAACAGTACAAAGAATGGTGTGCCACACAAACCACAGCTCGAGTTGTTATCACCAAAAGACTGACTGAGAAAACTGATTTATTCCACGGAATCAACGGTTACTGGACAAATACATGGATGCAATATATCAGGGCATCTCGACCGTCAGAAGACGCTTATGGGCGATAACCCTTGGTGAAATGCACCCACAGAATCGCAGAACGGTTCATCAGCCAAGGCCAGGGCGATAACCCTTGGTGAAATGCACCCACAGAATCGCAGAACGGTTCATCAGCCAAGGCCAATCCTTAAGGCCAAGAACATTCAGACAATATTTATGGACCGCATAGCGCAAACAAGAAAAGAAAATCAGCATTATAGAGGTTTTACATGGTACACAGAAAAGGGAACGAAATCATAGAGACAGCCATGTGGCAGAGCCAGGACAAAAAAGAGGTGGCAAGAGGATTGGAGCAACCAAATCACAGCCATCCATATCCAAAAGGCCAACCTCCGCCTCACAACTCATATCCCTTGTGCGCCCAGGTGCTCACCCTTATATCTCAGCGGTTGCTGCACTTCTCACCCCCCCTAAGGAAAGGCTGCAATTGTAAGGCGTGTCAAAGAAGAGGGTAGCACCTGATCTGCTTGCTTCCAGAAACAATGGCGTCTGCTACTCTCCTCAAATCGTCTTTCCTTCCCAAGAAGGCCGAATGGGGCACCACGCGCCAGGCCGCCGCCCCCAAGCCGGTGACCGTCTCCATGGTTGTCCGTGCCAGCGCATACGCCGATGAGCTTGTCAAAACCGCGGTATGCTTTCTTAACACCAACAGTATAATTCTAAAATTTTGCCATGCATATGCGTGTATTGGTTTGATCCTTGATGGGCAGTAACTTCTGGTGAGAATCCTCTTTACATCAGTGGGAAGGGGAGATCTGGATGTATTGTCAATACAGAAACTACCAGTGATAAATCAAATGTATTCTTATTAGGACGAACAGTTAACCTAGTACCGCTTATCATAATCCCTTCGACATAAGACGGATGTTTTGTCACGCAGAGGAAATTGTTTGGCTGCTTTTGATCACACCAAAATCACTAGTCTAAATGAACATGCATAACATATGTTTTCGATATTGCATTGGTACAGTAACTTTTCTATCTTCACCGCAGCGAGCTAAATAGATATGATTGATGTTGTGTTGTACCAAAAAAGAGCTTGTGAAAATCCAAACATAAACGTGATGTGTTTCTCGATTAGACATCTGCAGTAAGACATGACACGGGTTAATCACCACTAGCTTAACGATATGGCAGGATGCATCATGGCGCACAGAAATTAAACAACTCGCATTCCAGTGATATCATTGTTTGAATCCATGTGTCCGGCTTAAGCTGAGCAATCCATCTGAAGGTTCATAAGTCATAACAAAACTACATAGTTTCTTCTAAACTTTAAATCTTTGATAGCATAAATCATCAAGTTTACAACAGTAAAATTTCTACCAAACGAGCAAAGGTTTCATTCGGTTATGGACTTATGGTTCAGAAGATCTCAAGCTTTATTCTTCCTGCTGCAGAAAACCATCGCATCGCCAGGCAGGGGTATCCTTGCCATGGATGAGTCGAATGCCACCTGTGGCAAGAGACTTGCCTCGATTGGCCTTGAGAACACCGAGGCTAACCGCCAGGCTTACCGGACCCTTCTTGTCACTCCACCAGGCTTGGGAAACTACATCTCTGGTGCTATCCTCTTTGAGGAGACGCTCTACCAGTCGACTGTTGATGGAAAGAAGATTGTTGACATCCTTGTCGAGCAGGGAATCGTTCCCGGTATCAAGGTTGACAAGGTAGGCTGTCCACTGATTTCACTCCAAATTGGCACATGCATTCCCTTCTTAGAGCCTTTTTTAATTAAAACAATTCTAAACTGAAGATCCAAACTGCTACTATTACTAATGCATCAGAACAATCATATGTGCTCATGTGACTAGCTATACATTACAAGCAAAAACTGATGATCCCGGGTTTATTTCAAGCTTAGAAACAAAACATTCTTCTAAACTGGTGCTCATTCTCAGGGTCTTGTGCCACTTGTTGGTTCCAATGATGAGTCGTGGTGCCAAGGTCTCGATGGCCTTGCCTCCCGTGAAGCAGCATACTACCAGCAAGGCGCTCGCTTCGCCAAGTGGTTAGATATTTGCTCCTACAAGGACAGTTTACCTTCCTTTAGGTCACCCTAGCTGACATGAAGTTCTCTTGCAGGCGCACTGTTGTCAGCATTCCTAACGGCCCATCTGAGCTTGCTGTCAAGGAAGCTGCCTGGGGTCTTGCCCGTTACGCGGCCATCTCACAGGTATTGTTCCCATTTCCTAACAGTCCAAACTTCTACAGCACAATGGTCAGGCACAACATTCTAATGGATATGTAAACCTCTTGTTTGTTGATGTTCTAGGACAATGGGCTGGTGCCGATTGTGGAGCCTGAGATCATGCTCGACGGTGAGCACGGCATCGAGAGGACCTTCGAGGTCGCACAGAAGGTGTGGGCGGAGACCTTCTACTACATGGCTCAGAACAACGTCATGTTTGAGGGCATCCTCCTGAAGCCAAGCATGGTGACCCCTGGTGCTGAGTGCAAGGACAGGGCCACCCCTGAGGAAGTAGCCAGCTACACCCTCAAGCTCCTCCAGAGAAGGATCCCCCCTTCCGTCCCCGGCATCATGGTGAGCAGAGCACCCTCCCAAACCTAAGTGCACAATCATGAATATAAGATTATGTTGACAAGACACTATCCTCGACAACCAATTTGCATGGTGACATAGTAACTAACTCAAAAGTGCCAAAATAGTATGCCTTCAGAAATCCACATACCATTATATTATTTCTGCAGAAGCTTACATCCTATGTTGTTTCTTTTCTGTGGTGGTGCAGTTCTTGTCTGGCGGTCAGTCGGAGGTTGAGGCAACGCTGAACCTCAACGCGATGAACCAGGCGCCGAACCCGTGGCACGTGTCCTTCTCCTACGCGCGGGCGCTGCAGAACACCTGCCTCAAGACGTGGGGCGGGCGGCCGGAGAACGTGGCCGCGGCGCAGGAGGCGCTGCTGCTGCGCGCCAAGGCCAACTCCCTGGCGCAGCTCGGCAAGTACACCAGCGACGGCGAGGCCGCGGAAGCCAGCGAGAACATGTTCGTCAAGAACTACAGCTACTGATCCGTCGGCGAGCTGTCTATCTAATAATCTACCGCATCACTGTCAGCCAGTAGCGCCTTGAGCAGCTTGAGGGCGCCTTAGCCAGCCAGTGGGTCGTCGTTGTTACAGGTCGAGACCAGCTCTGATGGAAGAGATGGACAGAGAGTATATACTGTGTTTTGCCACCCTAAGTAAACTACTTTGGAATGGTCCTACTGTTGTATTCGCATGGTTTTTTCTAGAGCATTGTTGCCTTTGTTTGCTACCGTAAGTATGAACAATCTGAAGCGCCCCCCCTTTGGAACGCGGTAATTTTTCTGAGATATTATTTCTATCGGGTTTGCTATTTCACATCTAGATGTAAAATAACTATCTCGCATTTAAATCTAGAATCGTTGGATTATTTGTCTTTAATATTTGTGCAAAATACAATGGTCTAGTTGATGCGCTGTCTCGCGCTGCC

>TaFBA5

GTCGCGGAGACCAGCCACCTGCACCTTGCCGCCGCCGACCATGGCCGCTGCTGCGCCGGCCCGCAGCCTCCGCCACCCGGATCCGCCGGGGCACAGCCAAGCAGCCGCAGCCGCGCCCCCAAAGGGTCGCGCCGCGCCGCCCGCGTGCTCGCCACGCCAGTCCAGAGGCCCGACCCTCTGGATCTGGGGCCGCCGTCGCGAAACCCTAGGTTCGGAGAAGCGCAGCTCCGTCAACCTCGTCGAGCCCCACGCTCGAGGGGGGAGGCCGCCTGGGAGCGAAGTCCCGCCGCCGCCGGATCAACCAGGGCTTTGCCCGGCGGGGAGCACCGGCGGCGGCATGGGGAGGGGGAGGAGGAGGGGAGGGAGCGCTCGAGGGCTGCGTCGCCTCCCGAGTCGCCCGTGGGGAGGGCGATGCGGGGGAGGGGAGGGCCATGGGTCCGAGTTATAGGACATCGTCTGATCTAAGTAGGCCAACCTCGTGTACTTGTAAACAGTTTGTAATTACCGGCTTTTGTTATTCAATAGTAAAATCCCTTTCATGGTATTTTGTGTCTTGTCCTTTTATTGAGGGCACTAGGTGCCACATACCAACTCGCTCTCTGATGTTAATGTTTATGGTGAGTAGACAAAGGTGCCAAATTCCTTTTCTGCTTTACGCTTTGAACTTTTATATCAAACACCCCCAGTCTGGAAAATATGCATTTTTGTATCCTTGTTTCTCTGTTTTTCTCCTAGCCATTGTGACACTTCCTAGTTCCTACTAATTCATTTTGATATGAAATATATTATGACATATAAATTCAGATATTTTAGTAAGGCTAGAAAAGATCATTTTTTTCCTAGGGAAAGATTCATGGGGTCCAGGTATTATATACTCCCTCCGTCCAGAAATACTTGTCGGAGAAATGGATGTACATAGATGTATTTTAGTTCTAGATACATCCATTTTTATCCATTTTGACGACAAGTAATTCCGAACGGAGGGAGTACAAAATTATTATATGGTTGGTATCCTCCTGATGGCATCCTTGGCAGGACTGTCATGTTGATGGTGGATGTGTTTTCTGCAGCCAGGCACTGGGGCACTGGGGCACTGAGCCAGAACAAAGAATGGTGTGCCACACAAGCCACAGCTCGAGTTGTTATCACCAAAAGACTGACTGAGAAAACCGATTTATTCCACGGAATCAACGGTTACTGGACAAATTCATGGATGCAATATATCAGGGCTTCTCGACCGTCAAAAGATGCTTATGGGCGATAACCCTTGGTGAAATGCACCCACAGAATCGCAGAACGGTTCATCAGCCAAGGTCATCCTCAAGGCCAAGAACATTCAGACAATATCTATGGACCGCATAGCGCAAACAAGAAAAAAAATTCAGCATTACAGGTTTTACATGGTACACAGAAAAGGGAACGAAATCATAGAGACAGCCATGTGGCAGAGCCAGGACAAAAAGGAGGTGGCAAGAGGATTGGAGCAACCAAATCACAGCCATCCATATCCAAAAGGCCAACCTCCACCTCACAACTCATATCCCTTGTGCGCCCAGGTGCTCACCCTTATATCTCAGCGGTTGCTGCACTTCTCACCCCCTAAGGAAAGGCTGCAATTGCAAGGTGTGTCGAAGAAGAGGGTAGCACCTGATCTGCTTGCCTCCAGAAACAATGGCGTCTGCTGCTCTCCTCAAATCGTCTTTCCTTCCCAAGAAGGCCGAATGGGGTGCCACGCGCCAGGCCGCCGCCGCCAAGCCGGTGACCGTCTCCATGGTTGTCCGTGCCAGCGCGTACGCCGATGAGCTTGTCAAAACCGCGGTATGCTTTCTTAACACCAACAGTATAATTCTTCAATTTTGCCATGCATATGCGTGTATTAGTTTGATGGGCAGTAACTTCTGGGTAGGATCCTCTTTACATCAATGGGAAGGGGAGATCTGGATGTGTTGTCAATACAGAAACTACCACTGATAAGGAGATAACTCAAGTGTATAATGTATTCTTATTAGGATGAACAGTTACCCTAGTGACGTTTATCATAATCCAGCAGACGTAAGACCAATGTTTTGTCAGGCAGAGGAAAATTATTTGGCTGCTTTTGATCATACCAAAACCACTAGTCTGAATCAAAATTCATAACATATGTTTTCTATATTGCATTGGTACAGTAATTCATGATGCATAACTTTTTTATCTTCGTCGCTGCAAGCTCATGTTGTGTTGTACCAAAAAAGAGCTTTGAAACAACAAACATAAACGTGATATCTTTCTGGATTAGACATCTGCAGTAAGACATGACGCAGGTTAATCACACTATCTGTAATGATAAGGCAGGATGCATCATGGTGCACAGAAATTAAACAACTCGCATTCCAGTGATATCATTGTTTGAATCCATGTGTCCTGCTTAAGCTCCGCAATCCATCTGAAAGTTTATAGTTGTTTTTAAACTTTAAATCTTCGATAGCATAAATCATCAACTTTACAACAGTAAAATTCCTACCAAACGAGTAAAGCTTTCATTTCAAGTTATGGTTCAGAAGATCTCAAGCTTTATTGTTCCTGCTGCAGAAAACTATCGCATCGCCAGGAAGGGGTATCCTTGCCATGGATGAGTCGAATGCCACTTGTGGCAAGAGACTTGCATCGATTGGCCTTGAGAACACTGAGGCTAACCGCCAGGCTTACCGGACCCTCCTTGTCACTCCACCAGGCTTGGGAAACTACATCTCTGGTGCTATCCTCTTTGAGGAGACCCTCTACCAGTCGACTGTTGATGGCAAGAAGATTGTTGACATCCTTGTCGAGCAGGGAATCGTTCCCGGTATCAAGGTTGACAAGGTAGGCTGTCCACTGATTCCACTCCAAATTGGCACATGCATTCCCTTCTTAGAGTTTTTTTTAATTAAAACAATTCTAAACTAAAGATCCAAACTGCTACTATTACTAATGCATCAGAACAATCATATGTGCTCTTGTTGATGAACCACATGGCATGATTCTTCATGTGAGTAGCTATACATTAAAAGCAAAAGCAGATGATCCCGGGTTTATTTCAAGCTCAGAAAACAAAACATTCTTCTAAACTGGTGCTCATTCTCAGGGTCTTGTGCCACTTGTTGGTTCCAACGATGAGTCATGGTGCCAAGGTCTAGATGGCCTTGCCTCCCGTGAAGCAGCATACTACCAGCAAGGCGCCCGCTTCGCCAAGTGGTTAGATATTTGCTCCTACAACTACAAGGACAGTTTACCTTCCATTAAGTCACCCTAGCTGACATGAAGTTCTCTTGCAGGCGCACTGTTGTCAGCATTCCTAACGGCCCATCTGAGCTTGCTGTCAAGGAAGCTGCCTGGGGTCTTGCCCGTTACGCGGCCATCTCACAGGTATTGTTCCCATTTCCTAACAGTCCAGACTTCTAGAGCACAATGGTGAGGCACAGCATTCTAGTAGATATGTAAACCTCTTGTTTGTCGATGTTCTAGGACAATGGGCTGGTGCCGATTGTGGAGCCTGAGATCATGCTCGACGGTGAGCACGGCATCGAGAGGACCTTCGAGGTCGCACAGAAGGTGTGGGCGGAGACCTTCTACTATATGGCCCAGAACAACGTCATGTTTGAGGGCATCCTCTTGAAGCCAAGCATGGTGACCCCTGGTGCCGAGTGCAAGGACAGGGCCACCCCTGAGGAAGTAGCCAGCTACACCCTCAAGCTTCTCCAGAGAAGGATCCCCCCTTCCGTCCCCGGCATCATGGTGAGCAGAGCACCCTCCAAAACCTAAATGCACAATCATGAAGCATCTTGGTTTCTCATTGTTCAAGAATATAAGATGAACCAAAATGCAAAAATGGCAGTATGGCTTCAAACTCCAGAAATCACTATCTCTGCAGAAGCTCACATCCTATGTTGTTTCTTTTCTGTGGTGGTGCAGTTCTTGTCTGGTGGTCAGTCGGAGGTGGAGGCGACGCTGAACCTGAACGCGATGAACCAGGCGCCGAACCCATGGCACGTGTCCTTCTCCTACGCGCGGGCGCTGCAGAACACCTGCCTCAAGACGTGGGGCGGCCGGCCGGAGAACGTGGCCGCGGCACAGGAGGCGCTGCTGCTGCGCGCCAAGGCCAACTCCCTGGCGCAGCTCGGCAAGTACACCAGCGACGGCGAGGCCGCGGCCGCCAAAGAGAACATGTTCGTCAAGAACTACAGCTACTGATCCGTCGGCGAGCTATTCTATCTATGGAGGGAGAGTGCTAGTAGCGCCTTGAGCAGCTTGAGGGCACCTTAGCCAGCCAGCGTGTCGTCGTTGTTACAGGTCGAGACCAGCTCTGATGGAAGAGATGGACGGAGAGTATATACTGTGTTTTGCCACCCTAAGTAAACTACTTTGGAATGGTTTTACTGCTGTATCCGCATGATTTTTCTAGTGCATTGTTGCCTTTCGTTTGCCACCCTAAGTATGAACAATCTCAAGCGCCCCGTTGGGAACGAGGTATTTTTTCTGGGATTATATATTGTTCTGTTTCCTGGAACCACTGCAGATCCCACGTGAAATTCCTGTGTTC

>TaFBA6

GAAAACTATGACAAGTGAAGAGTTTCTCAGCCGATGCTATATCTGATTAATCTGAAGCATGAAATGAATTTAATTGAAGGGTAGACCGCTTCTTGTGATTCATTATCATTTGAAGCTAATTGAAGTGCTGGCAGCAAAGCAACAACTCTCTGCACACACTCATCATCTGCTTGAACTGATGAAAGAAGCCTCATGGCCCTTTTGACAACACCAAGATAAATACACAGTATGTGGATTGAATGATGTGATCAGACTTCAAGACCTGGTGGCATGTGCAGCACAGGCATCATGCACATTAAGCTTGTGATAGCGAGTGTTTTTGTTCAGGCATGCTAATACACCTCATGGGAGAAGTCACATACACCATAGGGAGTCTCTATTGATGTATTTCTGCGGAGTTATAGGACATCGTCTGATCTAAGTAGGCCAACCTTATGTACTTGTAAACTACTCCCTCCGTCCGGAAATACTTGTCATCAAAATGAATAAAAGGGGATGTATCTAGATGTATTTTAGTTCTAGATACACCCCTTTTTGTCTATTTGAATGACAAGTTTTTTTGGACGGAGGGAGTATTTGTAATTACTGGCTTTTGTTATTCAGTAGTAAAATCCCTTTCATCGTATTTTGTGTCTTGTCCTTTCGTTGAGGGCACTAGGTGCCACATACCAACTCGCTCTCGGATGTTAATGTTTATGGTGAGTAGACAAAGGTGCCAAATTCCTTTTCTGCTTTACGCTTTAAACTTTTATCAAACACCTCCAGTCTGGAAAATATGCATTTTTGTATCCTTGTTTCTCTGTTTTTCTCCTAGCCAGTGTGACACTTCCTAGTTCCTACTAATGCATTTTGGTATGAAATATATTATGACATCTAAATTCAGATATTTTAGTAAGGCTAGGGAAAGATCATTTTTTCCTAATTGAAAGCATTATATCAAGAATCATGGGGTCCAGGGATTACATACAAGATTATTGCATGGTTGGTTTCCTCCTGATGGCATCCTTGACAGGGAGTGTCATGTTGATGGTGGATGTGTTTTCTGCAGCCAGGCACTGGAGCACAAGGAGAAGCAGCACTCTGTCTTAGAGCCAGAACAAAGAACGGTGTACCACACAAGCCACAGCTCGAGTTGTTATCACCAAAAGACTGACTGAGAAAACCGATTTATTCCTTGAAATCAACGGTTACTGGACAAATACATGGATGCAATATATCAGGGCATCTCGACCGTCAGAAGACACTCATGGGTGATAACCCTTGGTGAAATGCACCCACAGAAACGCAGAGCGGTTCATCGCCAAGACCATCATCCTTAAGGACAAGAACATTCAGACAATATTTATGGACCGCATAGCGCAAACAAGAAAAAAATTCAGTATTATACAGGCTTTACATGGTACACAGAAAAGGGAACGAAATCATAGAGACAGCCATGTGGCAGAGCCAGGACAAAAAGGAGGTGGCAAGAGGATTGGAGCAACCAAATCACAGCCATCCATATCCAAAAGGCCAACCTCCACCTCACAACTCATATCCCTTGTGCACCCAGGTGCTCACCCTTATATCTCAGCGGTTGCTGCACTTCTCACCCCCCCTAAGGAAAGGCTGCAATTGTAAGGTGTGTCAAAGAAGAGGGTAGCACCTGATCTGCTTGCCTCCAGAAACAATGGCGTCTGCTACTCTCCTCAAGTCGTCTTTCCTTCCCAAGAAGGCCGAATGGGGCGCCACGCGCCAGGCTGCCGCCCCCAAGCCGATGACCGTCTCCATGGTTGTCCGTGCCAGCGCGTACGCCGATGAGCTTGTCAAAACCGCGGTATGCTTTCTTAACACCAACAGTATAATTCTACAATTTTGCCATGCATATGCGTGTATTAGTTTGATGGGCAGTAACTTCTCGGGAGAATCCTCTTTATTACATCAGTGGGAAGGGGAGATCTGGATGTATTGTCAGTACAGAAACTACCAGAGATAACTCAAATGTATTATTAGGATGAACAGTTACCCGGTACCGTTTATCATAATCCATTGGACGTAAGACCAATGTTTTGTCAGGCAGAGGAGTATTATTTGGCTGTTTTTGATCATACCAAAATCTCTAGTCCGAATTAACATTCATAACGTATTTTTCTATATTGCATTGGTACAGTAACTTTTCTATCTTGATCGCTGCAAATTAAATAGATATGATTGATGTTGTGTTGTACCAAAAAGGAGCTTGTGAAACTCCAAACATAAACGTGATATGTTTCTGGATTAGACATCTGCAGTAAGACATGACACAGGTTAATCAGCACTAGCTGTAATGATAAGGCACGATGCATCAAGGCACACAGAAATTAAACAACTTGCATTCCATTGATATCATTGTTTGAATCCATGTGTCCTGCTTCAGCTCAGCAATACATCTGAAGGTTCGTAAGTCATAACAAAACCACATAATTGCTTTTGACTTGAAATTTTGATAGCATAAATCATCAACTTCACAACAGTAAAATCTCTACTAAATGAGTAAAGCTTCCGTTCAGTATGGTTCAGAAGATCTCAAGCTTTATTATTCCTGCTGCAGAAAACCATCGCATCGCCAGGCAGGGGTATCCTTGCCATGGATGAGTCGAATGCCACCTGTGGCAAGAGACTCGCCTCGATTGGCCTTGAGAACACTGAGGCTAACCGCCAGGCTTACCGGACCCTCCTTGTCACTCCACCAGGATTGGGAAACTACATCTCTGGTGCTATCCTCTTTGAGGAGACCCTCTACCAGTCGACTGTTGATGGCAAGAAGATTGTTGACATCCTTGTCGAGCAGGGAATCGTTCCCGGTATCAAGGTTGACAAGGTAGGCTGCCCACTGATTTGTGATTTCACTCCAAACTGACACATGATTTTCTTCGTGCATTCCCTTATCAGAGTTTTTTAATTAAAACAATTCTAAACTAAAGATTCAAACTGCTACTATTACTAATGCATCAGAAGAATCATATGTGCTCATGTGACTAGCTATACATTACAAGCAAAAACTGATGATCCCAGGTTTATTTCAAGCTTAGAAACAAAACATTCTTCTAAACTGGTGCTCATTCTCAGGGTCTTGTGCCACTTGTTGGTTCCAACGATGAGTCATGGTGCCAAGGTCTCGATGGCCTTGCCTCCCGTGAAGCAGCATACTACCAGCAAGGCGCCCGCTTCGCCAAGTGGTTAGATATTTGCTCCTACAACTACAAGGACAGTTTACCTTCCATTAAGTCACCCTAGCTGACATGAAGTTCTCTTGCAGGCGCACTGTTGTCAGCATTCCTAATGGCCCATCTGAGCTTGCTGTCAAGGAAGCTGCCTGGGGTCTTGCCCGTTACGCGGCCATCTCACAGGTATTGTTCCCATTTCCTGACAGACCAAACTTCTACAGCACAATGGCGAGGCACAACATTCTAGTGGATATGTAAACCTCTTGTTTGTTGATGTTCTAGGACAATGGGCTGGTGCCGATTGTGGAGCCTGAGATCATGCTCGACGGTGAGCACGGCATCGAGAGGACCTTCGAGGTCGCGCAGAAGGTGTGGGCGGAGACCTTCTACTACATGGCCCAGAACAACGTCATGTTTGAGGGCATCCTCCTGAAGCCAAGCATGGTGACCCCTGGTGCCGAGTGCAAGGACAGGGCCACCCCCGAGGAAGTGGCCAGCTACACCCTCAAGCTCCTCCAGAGAAGGATCCCCCCTTCCGTCCCCGGCATCATGGTGAGCAGAGCACCCTCCCAAACCTAAATGCACAATCATGAATATAAGATTATGTTGACAAGACACTATCCTCAACAACCAGTTTGCATGGTAACTAAAAAGTGCCAAAATAGTATGCCTTCAGAAATCAACAAACCATTATATTATTTCTGCAGAAGCTTACATCCTATGTTGTTTCTTTTCTGTGGTGGTGCAGTTCTTGTCTGGCGGTCAGTCGGAGGTGGAGGCGACGCTGAACCTGAACGCGATGAACCAGGCGCCGAACCCGTGGCACGTGTCCTTCTCCTACGCGCGGGCGCTCCAGAACACCTGCCTCAAGACGTGGGGTGGGCGGCCGGAGAACGTGGCCGCGGCGCAGGAGGCGCTGCTGCTGCGCGCCAAGGCCAACTCCCTGGCGCAGCTCGGCAAGTACACCAGCGACGGCGAGGCCGCGGAAGCCAGCGAGAACATGTTCGTCAAGAACTACAGCTACTGATCCGTCGGCGAGCTATCTATCTAATTATCTACCTATGGAGGGAGAGTGCTAGTAGCGCCTTGAGCAGCTTGAGGGCACCTTAGCCAGCCAGTGTGTCGTCGTTGTTACAGGTCGAGACCAGCTCTGATGGAAGAGATGGACGGAGAGTATATACTGTGTTTTGCCACCCTAAGTAAAAAACTTTTGGAATGGTTTTACTGCTGTATTCGAATGGTTTTTCTAGAGCATTGTTGCCTTTGTTTGCCACCCTAAGTATGAACAATCTCAAGCGCCAGGTGTGGAATGCGGTATTTTTTCTGGGATTATATGTTGATCTGTTTTCTGGAACCACGAGCTCGCGTGAAATTTCTGCGT

>TaFBA7

TATGACGTATACGCTCCACCCGCGGGGAAGCCTTGCGGCAGCCCAGTATCGGTCAAGAATTTGTGTGAAACTAGTTTCACAGAAAACTTGTAGTTCAAGGCATAGTCCACTATTCAAGTTGTGATCTATTGTAGCATAAATTTCTAAGTGGAAGTTCAACTTCACAGTCTCCACTAAGCACCGATATATAAAACAATGTTTCGGATCTAAATGATGAGATGTGCCAATGAGACTTTGTGGGGGATTGTTGGAATTTTGCTAGTAGGCCTTTGGCCCAAAACCCAACGAAAATTCTGAAATTCTCTTGGCCCATTCATGCACACATGTGAGTGGAGTGAGTGAGGCTAAAGTTTAGTCCCACCCCGGAAGTTGAGAGAGAGTTGCACCTCTTTATAAGGTGAGCTCTTCTACCACTTATATGAGCATGAGAAGAGGAGACCTACACGCGCGCTCCTCCTCCTCGCTCTCGCCATGCCACGCCACGCCACGCCACGCCACGCCACACCTTGTCACGACGCGCCGCGGATTGCGGGATTGAGCCGAGCCGGGGACAGAGCTATGCACGTTGTCTATATTTTTGCTGCATGGGAAAATTAATGAGTCATTAATTAATAATTAACGGATGCGTTAATTACTAAACCGTTTCCAATTCTTTTGGATCGTGACGACTCGGACGTGGGATTTACTCCCACGACCTATCCGGCCCGCACTATATAGTCAGGCATATGTCTACCCTAGCCGCCGCCGCTTCGTATGGTTTCTCACCACCGTTCCAGATCATTGCGCCGCCAAGCAAGTCTTCTCCATTCCTCCTTCCGGCGTGCACCGCGAGAAGGGACAGCAGTCCTCTGGAACCCCGCCTCTCGTGATCCTGTACGGGAGAGGGGCGATCAGGTTTTTGGGGAGCGCACTCGCGCGACTGCTGGCAGCGACGACTTCGCGAACGACGACTTCTTCCCCGACCTCGGCAACCTCGTCCTCGACGACATGGGCGACAACGTCAACGCCGGCGGTGCTGCACCGTATGTGATTCTATCCTTCCTGTTCGAGATCGTGGTAGAATTCATGCTTCTAGTATGTGCCCTAGATGTGATATGTTCATCTGCTATGCTAATTCGCATGATTAATTTAATCTCTGCTGTTGTGGACATGATTTATTTTTTGTTTATTCGAATTAAATTTCGTAGTAATTTGCTCATATTTCCAACAGCAACTTGCTGAGATCCATCCGGAAAAGAAAAAGAGCCAAGCGGAAGCATGGCAACCACCCCGGGCGGGGTGAATCTATCTATCTATCTATCCATGGATGCGAGAAATCTGTGGCATAAGCTACCGGTGAAACGCGGCCGCAGGTGATGATGTCAGCCATCTTGAGGCCAGCAGCGTTCCCACGGATGGATGAGGTGCAGGGATTTTCTTGCCTTTTTGTTTTACACGGTGCACATAAACATGGGGAATTGCAATTGTGCATTAAAAAAGGGGTGATGACTGGGGCGATGGACGGACAGCCAGACATTGAGCCATCCAAACCTCCACCCCACACCTCATATCCCCTCGTGCCACCCATTCAAATACGAGTCCATTGGCCTCAGCGAGGCAGAGAGGATAGCTAGCACCTTGAGCTTGAGGTCAGTGGCAGGCAGGCAAGCAAGAAAGTAAGAAGAAGAAGAAGAAAGGACATGGCATCTGCTACTCTCCTCAAGTCCTCCTTCCTCCCCAAGAAGGCCGAATGGGGCGTCGCGCGCCAGGTCGCCGCCCCGAGGGTGTCCATGGTCGTCCGCGCCGGCGCCTACGACGATGAGCTCGTCAAGACCGCGGTACGTACGCTGCAGGCAGCTTCTCGCCGGCCGAATTACACGTACTACTAGTATAATTCACTCATGGACTGGACTGGACTGACATGCTGCTGCTGCTTTGCACGCACGTGCAGAAAACCATCGCGTCGCCGGGGCGCGGCATCCTGGCCATGGACGAGTCCAACGCCACCTGCGGGAAGCGCCTCGACTCGATCGGCCTGGAGAACACAGAGGCGAACCGGCAGGCGTTCCGCACGCTGCTGGTCTCCGTCCCTGGCCTCGGCAACCACATCTCCGGCGCCATCCTCTTCGAGGAGACGCTCTACCAGTCCACCGTCGACGGCAAGAAGATCGTCGACATCCTGGTGGAGCAGGGCATCGTGCCCGGGATCAAGGTGGACAAGGGCCTGGTGCCGCTCACCGGCTCCAACGACGAGTCTTGGTGCCAGGGTCTCGACGGCCTCGCCTCCCGGGAGGCCGCCTACTACCAGCAGGGCGCCCGCTTCGCCAAGTGGCGCACCGTGGTCAGCATCCCCAACGGCCCCTCCGAGCTGGCTGTCAAGGAGGCCGCATGGGGCCTCGCCCGCTACGCCGCCATCTCGCAGGACAACGGTCTGGTGCCCATCGTGGAGCCGGAGATCCTGCTGGACGGGGAGCACGGCATCGAGCGCACCTTCGAGGTGGCGCAGAAGGTGTGGGCCGAGACCTTCTACCAGATGGCCCAGAACAACGTCATGTTCGAGGGCATCTTGCTCAAACCCAGCATGGTCACCCCCGGCGCCGAGTGCAAGGACAGGGCCACGCCGGAGCAGGTCGCCGACTACACCCTCAAGCTCCTCAGCCGCCGCGTGCCGCCCGCCGTCCCCGGCATTATGTTCCTGTCGGGAGGGCAGTCCGAGGTGGAGGCCACGCTCAACCTCAACGCCATGAACCAGGGGCCCAACCCGTGGCACGTCTCCTTCTCCTACGCCAGGGCGCTGCAGAACACGTGCCTCAAGACGTGGGGCGGCCGGCCGGAGAACGTCAAGGCGGCGCAGGAGGCGCTGCTGCTGCGTGCCAAGGCCAACTCACTCGCGCAGCTCGGAAAGTACACCAGCGACGGCGAAGCCGCCGAGGCCAAGGAGGGCATGTTCGTCAAGAACTACAGCTACTAATCCAACCATTCCAATCAACACTACTACGTACGTTCGTTACGAAAGCAGAAATTAAAGATGCATGCATGCATGCATGATGATACAGCTGCAAGTGCAAGATCGATCGAGCTCGGAGCCTGAGCCTGAGATGATGATTACTTGTATTTTCTAGATCTTTTTTTACTCGTTAATTTGCCTCTCGATATGCACAATTCATGCTACAGTCCAGTAGATTATTGTAAAGCTACTTTTACGTGTGCGATCCATGGATTTGCATGGTTACTGCAAAATTTTGCATGTACATACGTACACCATGAATTTACGTGTGCGTTTGTTGGTTCGTCTCGTCGTTTTTTTCGATAAAAGGGGCTTTTATTATCT

>TaFBA8

GTCCCTTCAAAATAGGAGTTCTAGCAGATGCAAAATATTGCTCCAAGTTCTGTATCCAAGCATTAACATTTATACCATCAAATTAAGAGATATTTTACTTGGCAGGTTTGACAATTAACACTGCTCTTCTACGCTCAACCGGGATAATAGGTCTCTGCCTTCTAAAATTTCCTTCCACAACTTCCTTTTCCACATATTCTTCAGACATTATTTGCTGTTGTGCTATGTGGCCTTGACTCTGTGAACCTTCAAGTCTTGTAGTGTCATAATTAGGATGTCTATAGGGTGTTTTTGGGGTGACATCCAAGTTAAGCTCTTTTCCTGTATGATCCCTTAACACTCCAGAGCCTCCAGTTGGAGGTGTTTGCTTTTCAGGTATATAAATAGGTGGTGGTATTTGGTTGGGCCGGCCAGCTGCTACACTAATAGGGATTGCTTTGTTTGGAGCACTATATTGGTCAGATTGTTCTTTAATTACTTCCTCTTCGTGTGACCGAGGATCCAAAAATAGGCGTCTAAAATTGGCCAGGATCTTGTCGAAGTTCTTTTGCATTGTGAGAAATTTTCTGTTCACCGTTGCTAGGAACTCCTTACGATCTTCTTCTTGATTTCTCTGTAAGCTTTTGACATTGAGTTAGAGACTCTAATTCCAAATTTGTGTTGTCCCTTGAATTAGTCACTATTGGCGATCTTGCTTTCTTGTAGACGCACGTTTGTAGAAAAGGACAAGGGTTGGGAAGGGAGGGATTCAGTCGGTGTCCTCCTCTCACCGGTCTACCACCGACGTTCACCGCCTACTGCGCGGCTCGTCTTCAAAAGATGCATAGTCAGGTAGCAGGGGTTGCTAATTTAACGCTGAAGAAGCACTCTTCAGCAACTAGACCGCGCCGTTGATTGCACCCGAACTAGTGCATGAAAGTACTGTAGCTTGCCACAGGCAATTAGGTTTACAACCCGATTACATCAAGATGGTCTCGGAATGAATGGAGAACGTGTCCTTATAGCCATTACAACAGGGAGCAGGTGAAAAGTAAGGCTGAAAGAAGAAAAGGTGCATGACGGGGCAACAACGATGCTCGAGAGCTTGGGGACCGTCTGATGCTTGATCAACTTTTGACAAAGATGCTCACGAGGTGAACCGGTATCTAGCTAGGAGAGCCGTTAGATGCTTGATGGACGGCTCAAGATCCATTCAAAACGCGGGAACAGGGGAGCCGGTGATACATTCAGTTAAGTTCAGTTACAGAACCTGACAGTGAGTCAGAAATCACATACTACTACTGTGCTAGCAAGCAGCTCACAACTTGCAACTTGCTGAGATCCATCCGGGAAAGAAAAACAAGCGGAAGCATGGCAACCACCCCGGGCGGGGTGAATCTATCTATCTATCTATCCATGGATGCGAGAAAGATCTGTGGCATAGGCTACCGGTGAAACGCGGCCGCAGGTGATGATGTCAGCCATCTTGAGGCCAGCAGCGTTCCCACGGATGGATGAGGTGCAGGCCTTTTGTTTTACACGGTGCACATAAACATGGGGAATTGCAATTGTGCATTAAAAAGGGATGGACGGACAGCCAGACATTGAGCCATCCAAACCTCCACCCCACACCTCATATCCCCTCGTGCCACCCATTCAAATATGAGTCCATTGGCCTCAGCGAGGCAGAGAGGATAGCTAGCACCTTGAGCTTGAGGTCAGTGGCAGGCAGGCAAGCAAGAAAGGAAGAAGAAGAAAAAAGGACATGGCATCTGCTACTCTCCTCAAGTCCTCCTTCCTCCCCAAGAAGGCCGAATGGGGCGTCGCGCGCCAGGTCGCCGCCCCGAGGGTGTCCATGGTCGTCCGCGCCGGCGCCTACGACGATGAGCTCGTCAAGACCGCGGTACGTACGCTGCAGCTTCTCGCCGGCCGAATTACACGTACTAGTATAATTCACTCATGGACTGGACTGGACTGACATGCTGCTGCTGCTTTGCACGCACGTGCAGAAAACCATCGCGTCGCCGGGGCGCGGCATCCTGGCCATGGACGAGTCCAACGCCACCTGCGGGAAGCGCCTCGACTCGATCGGTCTGGAGAACACAGAGGCGAACCGGCAGGCGTTCCGCACGCTGCTGGTCTCCGTCCCTGGCCTCGGCAACCACATCTCCGGCGCCATCCTCTTCGAGGAGACGCTCTACCAGTCCACCGTCGACGGCAAGAAGATCGTCGACATCCTGGTGGAGCAGGGCGTCGTGCCCGGGATCAAGGTGGACAAGGGCCTGGTGCCGCTCACCGGCTCCAATGACGAGTCTTGGTGCCAGGGCCTCGACGGCCTCGCCTCCCGGGAGGCCGCCTACTACCAGCAGGGCGCCCGCTTCGCCAAGTGGCGCACCGTGGTCAGCATCCCCAACGGCCCCTCCGAGCTGGCCGTCAAGGAGGCCGCCTGGGGCCTCGCCCGCTACGCCGCCATCTCGCAGGACAACGGTCTGGTGCCCATCGTGGAGCCGGAGATCCTGCTGGACGGGGAGCACGGCATCGAGCGCACCTTCGAGGTGGCGCAGAAGGTGTGGGCCGAGACCTTCTACCAGATGGCCCAGAACAACGTCATGTTCGAGGGCATCCTGCTCAAACCCAGCATGGTCACCCCCGGCGCCGAGTGCAAGGACAGGGCCACGCCGGAGCAGGTCGCCGACTACACCCTCAAGCTCCTCAGCCGCCGCGTGCCGCCCGCCGTCCCCGGCATCATGTTCCTCTCGGGAGGGCAGTCCGAGGTGGAGGCCACGCTCAACCTCAATGCCATGAACCAGGGGCCCAACCCGTGGCACGTCTCCTTCTCCTACGCCCGGGCGCTGCAGAACACGTGCCTCAAGACGTGGGGCGGCCGGCCGGAGAACGTCAAGGCGGCGCAGGAGGCGCTGCTGCTGCGTGCCAAGGCCAACTCGCTCGCGCAGCTCGGAAAGTACACCAGCGACGGCGAAGCCGCCGAGGCCAAGGAGGGCATGTTCGTCAAGAACTACAGCTACTAATCCAACCATTCCAATCAACACTACTACGTACGTACGTTCGTTACCAAAGCAGAAATTAAAGATGCATGCATGATGATTCAGCTGCAAGTGCAAGATCGATCGAGCTGGACCCTCAGTCTGAGATGATGATTACTTGTATTTTCTAGATCTTTTTTTACTCGTTAATTTGCCTCTCGATATGCACAATTCATGCTACAGTCCAGTAGATTATTGTAAAGCTACTTTTACGTGTGCAATCCATGGATTTGCATGGTTACTGCAAAATTTTGCATGTACGTACGTACACCATGAATTGGCGATGAGGTTGGCGCTTGGCAGGGTTGGTTGCTTGTTGGGTTCGTCGTAGCCATACCTATGTGATCGTCTTGTCCCTGACCTGGAGCTCCTATGGAAGATCGAAAGAGAGGGAAAGAAACTATTCTGTGCAGCGCGCAACCTCCTGCTTTTCCTTCTTTCTCAATGGAAAACAAAGG

>TaFBA9

AGAGATATTTTACTTGGCAGGTTTGACAATTAATACTACTCTTCTATGGTCAACATGGGTAGTAGGCCTCTGTCTTCTGAAATTTCCTTCCACAACTTTCTTTTGCACATATTCTTCATACATTATTTGTTGTCGTGCTATGTGGCCTTGACTCTGTGCACCTTCAAGGCTTGTATTGCCATAATTAGGATGTCTATAGGGTGTTTTCCGGGTGACATCCAAGTTAAGCTCTTTTCCTGTATGTTCCCTTAACACTTCAGAGCCTCCAGTTGGAGGTGTTTGCTTTTCAGCTATATAAATGGGCGGTGGTAGTTGGTTGGGCCGGCCAACTGCTACACTAACAGGGGTTGCTTTGTTTGAAGCACTATATTGGTCAGATTGTTCTTTAGTTACTTCCTCTTCTTGTGGCCAAGGATCCAAAAATAGGCGTCTAAAATTGGCCTGGATCTTATCGAAGTTCTTTTGCATTGTGAGAAATTTTCTATTCACCGTTGCTATGAACTCCTCGAACTCCTTACGATCTTCTTCTCGATTTCTCTGTAAGCTTTTGACATCAAGTTCGAGGGACTCCAATTTCAGATTTGCGTTGTCCCTTGAATTAGTCACTTTTGGCCATCTTGCTTTCTTGTAGACGCACACTTGTAGAAAGGACAAGGGTTGGGAAGGGAGGGATTGAGTCGGTGTCCTCCTGTCACCGGTCTACCACCGCCTTTCACCGTCTACGGCGCGGCTCGTCTTCAAAAGATGCACGGTCAGGTAGCAGGGTTGCTAATTTAACGCTGAAAAAACACTCTTTAGCAGCCAGACTGCACCGCTGACTGCACCCAAACTGGTGCACGAAAGTACTGTAGCTTGCCACAGGCAATTAGGTTTACAACCAGATTACATCAAGATGGTCTCGGAATGAATGAAGAACGTGGCCTTATGGCCATTACAACAAGGAGCGGGTGAAAAGTAAGAATGAAAGAAGAAAATGTGCATGACGGGGCAACAACGATGCTCGAGAGCTTGGGGACCGTCCGATGGTTGATCGACGTTTGACAAAGATGCTCATGAGGTGAACCGGTGTCTACCTAGGAGAGCCGTTAGATGCTTGATGGGCGGCTCAGGATCCATTCGAAACGCGGGAGCAGGGGAGCTGGTGCCGCTGATACATTCAGTTAAGTTTAGTTACAGAACCTGACAGTGAGTCAGAAATCACATACTACTGTGCTAGCAAGCAGCTCGCAGCTTGCAACTTGCTGAGATCCATCCGGGAAAGAAAAACAAGCGGAAGCATGGCAACCACCCCGGGTGGGGTGAATCTATCTATCTATCCATGGATGCGAGAAATCTGAGGGCATAGGCTACCGGTGAAACGCGGCCGCAGGTGATGATGAAAGCCATCTTGAGGCCAGCAGCGTTCCCACGGATGGATGAGGTGCAGGAATTTTCTTGGCTTTTGTTTTACACGGTGCACATAAACATGGGGAATTGCAATTGTGCATTAAAAAGGGGGTGATGAGTGGGGCGATGGACGGACAGCCAGACATTGAGCCATCCAAACCTCCACCCCACACCTCATATCCCCTCGTGCCACCCATTCAAATACGAGTCCATTGGCCTCAGCGAGGCAGAGAGGATAGCTAGCACCTTGAGCTTGAGGTCAGTGGCAGGCAGGCAAGCAAGAAAGGAAGAAGAAGAAAAAAGGACATGGCATCTGCTACTCTCCTCAAGTCCTCCTTCCTCCCCAAGAAGGCCGAATGGGGCGTCGCGCGCCAGGTCGCCGCCCCGAGGGTGTCCATGGTCGTCCGCGCCGGCGCCTACGACGATGAGCTCGTCAAGACCGCGGTACGTACGCTGCAGGCAGCTTCTCGCCGGCCGAATTACACGTACTAGTATAATTCACTCATGGACTGGAGTGGACTGGACTGACATGCTGCTGCTGCTTTGCACGCACGTGCAGAAAACCATCGCGTCGCCGGGGCGCGGCATCCTGGCCATGGACGAGTCCAACGCCACCTGCGGGAAGCGCCTCGACTCGATCGGCCTGGAGAACACAGAGGCGAACCGGCAGGCGTTCCGCACGCTGCTGGTCTCCGTCCCTGGCCTCGGCAACCACATCTCCGGCGCCATCCTCTTCGAGGAGACGCTCTACCAGTCCACCGTCGACGGCAAGAAGATCGTCGACATCCTGGTGGAGCAGGGCATCGTGCCCGGGATCAAGGTGGACAAGGGCCTGGTGCCGCTCACCGGCTCCAACGACGAGTCTTGGTGCCAGGGCCTCGACGGCCTCGCCTCCCGGGAGGCCGCCTACTACCAGCAGGGCGCCCGCTTCGCCAAGTGGCGCACCGTGGTCAGCATCCCCAACGGCCCCTCCGAGCTGGCCGTCAAGGAGGCCGCCTGGGGCCTCGCCCGCTACGCCGCCATCTCGCAGGACAACGGTCTGGTGCCCATCGTGGAGCCGGAGATCCTGCTGGACGGGGAGCACGGCATCGAGCGCACCTTCGAGGTGGCGCAGAAGGTGTGGGCCGAGACCTTCTACCAGATGGCCCAGAACAACGTCATGTTCGAGGGCATCCTGCTCAAACCCAGCATGGTCACCCCCGGCGCCGAGTGCAAGGACAGGGCCACACCGGAGCAGGTCGCCGACTACACCCTCAAGCTCCTCAGCCGCCGCGTGCCGCCCGCCGTCCCCGGCATCATGTTCCTGTCGGGAGGGCAGTCCGAGGTGGAGGCCACGCTCAACCTCAACGCCATGAACCAGGGGCCCAACCCGTGGCACGTCTCCTTCTCCTACGCCAGGGCGCTGCAGAACACGTGCCTCAAGACGTGGGGCGGCCGGCCGGAGAACGTCAAGGCGGCGCAGGAGGCGCTGCTGCTGCGTGCCAAGGCCAACTCGCTCGCGCAGCTCGGAAAGTACACAAGCGACGGCGAAGCCGCTGAGGCCAAGGAGGGCATGTTCGTTAAGAACTACAGCTACTAATCCAACCATTCCAATCAACACTACTACGTACGTTCGTTACCAAAGCAGAAATTCAAGATGCATGCATGATGATACTACAGCTGCAAGTGCAAAGATCGATCGAGCTGGGAGCCTCAGCCTGAGATGATGATTACTTGTATTTTCTAGATCTTTTTTTACTCGTTAATTTGCCTCTCGATATGCACAATTCATGCTACAGTCCAGTAGATTATTGTAAAGCTACTTTTACGTGTGCGATCCATGGATTTGCATGGTTACTGCAAAATTTTGCATGTACGTACGTACACCATGAATTGGCGATGAGGTTGGCGCTTGGCAGCAGGGCGTTTGTTGGGTTCGTCG

>TaFBA10

GCATGTACTCAAAGACAGGCACTGCCCACTAGTGTTTTTTGAATCGGCCCCCATGTTTTTGTCCTCAAAATCTTAAAAGGTAAGAAAACGCTAGTTGGTTACAAAAGGCCACTTTGGACTCTAATAATAGTAGATGCCTGGTTTGATCAGTAGATGGTTCCCTTTATATTTCCTAAAGTACAATGGCCTAATTTCCCTATTTGTAGCACCAACCATAAGTCCATAATAACACCATCATAAGACAACATTTCTTTTGTTTGTACAAAAAAAAGAGTTTATATCAATCGATGCGGAGCTGTTTTTTTTTGCACATGGAAAATGGTACAATGTGGATCTTTTTTATATAGAATATACGCCATATGTTTCCGAGGAGTGGGAACCTAATTTTTCTTTTCTTTCTTTCAACCTAGGCTGACCCTTCGAACAAGCAAAACTGTGAGCTTAGTTTTGGGTTTTGCGTGCTCAAGAGTTTTGATCTTGTTTATATATAGCACTTAAAAAGGCATCCATCGTGTTGTTGCAAGTTTAAATTGCGGAGGTACGTGCTTTGCCTAATGGAGTATTTTTTTTGCAGGGCTTTACCTAATGGAGTAGGCCAGAGTAAATTTGAACGGTTGAGAGTCCATGTACATATACGCAAAGCACAATCACTACGCATTGGTATTGTTTTCTTGAATCGGCCCACGTATTGTTCCCCAATCATCTCATCTTGAAAAAGGTAAAGCCATGGTTTGCTTTAATAGTAGTAGACATATGCCTAATTTGATTAGCGTACGACTCACCTTCTTCCGTATATACAATGGCCCTTTTTCCCTGTAACACTTACACAAACCATACGTCCATAGCATCTTAAGTGAAAAAAAATGTGAATTCAAAAAAAGAAATCGAGTACAACACGGCGAAATATACCGCAACGCTCACATGTGTGATGCAGTAAATATTGTGCTGACATCATGCATGATCACGATCGTGCATGCACCCGATAGAATTCCCATTGTAAAAAAAAGATCATTTTGTTAAAATGTTAGCTGAAAGTCATAAAAAAATGAACAGTCATTTTTTTTGCAGGGTAACAAGTCATAACTAAACCTCCACTTTGTTCATGATTGCTCGCAGACGAGATGCATAAACAGGTGTGTCAACCATTCATAGCAAAATGACCAGCCGAGTATATGATATATCACATCAATGGACACATGGCTATTTTGTTTACACCCACCAGTACATTTTTGATGTGGAATACTTTTGATTCAAACCTTTTCTATTTGAAAAGATCTTTTTGGTTCATACACTATATGCACATACGAGTATATATTAAACACAAAATGTATCAAAGAAACTATGTAAACGATTAGGGAGTATCATGTCAGCACATATTCACGGTTTTACCAGCTGGTGCACAGGCCAGTCCTAATTAAGCTTCCAATTTTTTTCTAATCTAAATAAAGAAATTCACGTTCCCCTCTATTTTCAGAAAACGCGCACCTCCCAAATAAAGCCACCGCCGAGGGCCTGGATAAAAATAACAGAGGAAACCAGGGGCGGTGCGAGAGGACAGGCCAACGTTCCTACGCACCCAGATCGGATCCCCCCTTCCCTTTCTTTCCCCTCGTCCAAGCCGCGGCCCCTTTATAACCCCCACCCTCTAATCATCCCCGTCCACCAAAGCCGCCTCCCTCCCTCCCTCCCCTGCCTGCCAACCCCCAATCTCCCGTCGCAGCCCTCGAATCTCCCCCGAGCATCGACCATGTCGGCCTACTGCGGCAAGTACAAGGGTATGATCCGTCCTCTTCCCCCTCCTCCCGGCCCCCTCGCCCGTCGTTCTGTCGACATGCCTGTCTGCTTCGTGTGTAGATAGATCTGTTAACCGTTTTGCTGTTCTGCATGATTTAGATCTGCCGATGCTCGCGATTCCATGCCATGGTTTGTTAGATCCGCACACGCGGGTTAGATCCGTGCGCGCCGTGTTTGTGTCCGTTTGGTTCTAGCGGTTGGTTGTTTTTTCGGATCGTTCATAGGCGCTGGCAGTTGGATCGGGTGCTTAGGGGGTCATCGGTTGCGTCGTCCGATGATGGGTTGCATGCTGATAATTGTGCAAGTTGCTGCTGCTGCTAGGTGCATGCTAGTGATTATTTTGTGCGAATGATGATACTGGCGTTCCTTCCTTTTTTACTCTGTGGTTAAGTTTGTATACGCATCTACACATGTTTCTGTTGGGTTCGTCTTTAGTTATTGTAGATTGCATCTGTGAATCTAGATCTGTATATGTCATAGGATCATCTTTTGCCAAGGAATATGCCGTCCTTACATAGAGTTGTATGTTTTTCTCTCCTGTGATCTTAATTATTGGTATCCCCTGCCCTTATGTTTACCACCTTCCTGTTGTGCACGTGCAATTTTTTAGAAAAAATATTGACGAAATTTCGTAGTCAATTGTCAGAATTATGTTAGTAAATTTGATCTGCATTTGCTGTTATGCTTGCCAAACGTTGTGAGTGGTTGCACTCAACCTGCAGTTCTGTAGATTTGATCACAATTTATAATATATGTACAGTTATGGCAATTGATCACAATTTGTAATATCTGTACAGTTATGTCAATTGATTGTAATACTGTTGGTTGATTTATCTTGCAGATGAGCTCATCAAGAACGCTGCCTACATTGGCACCCCTGGCAAGGGTATCCTCGCTGCTGATGAGTCCACCGGCACCATCGGCAAGCGCTTCGCCAGCATCAATGTTGAGAACGTTGAGGACAACCGCCGTGCCCTCCGTGAGCTCCTCTTCTGCACCCCTGGTGCCCTCCAGTACCTCAGCGGTGTGATCCTCTTCGAGGAGACCCTGTACCAGAGCACCAAGGGTGGCAAGCCCTTCGTCGACATCCTCAAGGCGGGCAATGTCCTCCCCGGAATCAAGGTGGACAAGGGTACCATCGAGCTTGCTGGAACCAACGGTGAGACCACCACCCAGGGCTTTGATGACCTTGGCAAGCGCTGCGCCAAGTACTATGAGGCTGGTGCCCGCTTCGCCAAGTGGCGTGCAGTCCTTAAGATCGGCGCCACCGAGCCATCACAGCTCTCCATCGACCAGAACGCTCAGGGTCTGGCTCGCTATGCCATCATCTGCCAGGAGAATGGGCTGGTGCCCATTGTTGAGCCTGAGATCCTTGTTGATGGACCTCATGACATTGACCGCTGTGCTTACGTGACTGAGATCGTCCTTGCTGCCTGCTACAAGGCCCTCAACGACCAGCATGTCCTCCTTGAGGGCACCCTCCTGAAGCCCAACATGGTCACCCCTGGATCCGACGCCAAGAAGGTGGCCCCTGAGGTGATTGCTGAGTACACCGTCCGCACCCTCCAGAGGACCGTCCCTGCTGCCGTCCCCGCCATTGTCTTCCTCTCCGGTGGACAGAGTGAGGAGGAGGCGACCCTGAACCTGAACGCCATGAACAAGCTCCAGACCAAGAAGCCCTGGAACCTGTCCTTCTCCTTCGGGCGTGCCCTCCAGCAGAGCACCCTCAAGGCCTGGTCCGGCAAGACGGAGAACGAGGAGAAGGCCAGGACGGCGTTCCTGGTGAGGTGCAAGGCCAACTCCGAGGCCACCCTTGGCACCTACAAGGGCGACGCCACCCTTGGCGAGGGCGCCTCTGAGAGCCTCCACGTCAAGGACTACAAGTACTGATCTATCTGCCTTCGGAGCTGTGGCCCGTTTGGGCGTCCCGCTTACCAGTATCATTGTTTGGTTTTCTGAGTCCTGATATATCCTTGGATATCGAACATCGTTTTTAATAATATGGAACTTGTGTTTTCATAATACCGTAGTAGTTTCTTTTTGGAGTTGAAGTACCCTGGAAATCGGGTGCTGCGTATCGTATATTGGAGCAGGTTTTTGTTCCATGGTCCTGTAATTTTCCTGCTCATACAATGTTCAAACCTCATATGCGAGTACACATGTGTTCCGTCACCTTTTCTTGCTTTGTTCTAAGCTGTCAAATGGCTATCGATAAGTTTCATTCATGAAACGCGTTTGAGTTTTT

>TaFBA11

AAGGTGGAGTTTGTTAGAGTTGTGTCGAATATTGTGTACAAATAGGTTACAGTTGGACTCTGAGTAGTATTGTGTTTACAAGATATGGAGTCGTGTCCTAGTAGGACATTTGTATTCTAGGTCTCTTATATATATCGAGGGTAGACACACGATGTAACATATGCCAACATAGTAGCACAGGAATGCAGGGGAAGCCAGCGGCATGTGCCGACGTCCAGGGCGACTGCCGACGTCCAGGGCGACTGAGTGCGGTATTGTAGCGGTATCACGGGGAGGAGCGCCTGTAGTCAGGTCCGAGAGATGTAACCATATCGGTGAACCTCGTTAACAAATCTCGGTGTCGTGCATTGTGTAATTGCTTGGTTCTTGGATGATCGACGGAGTGTCTCGGATTTATTCTAATAGGGAGTGTATCTCTGAATGATAATCTGTTTGTGATCCTCAGATCTTTGATATTGCACTTTTTTTCTTTATAAGTTTTTTTCTAAATGGTTTTTTATAGAAGGTTTTTAATGAGACATTATTAATGCAGTCATTGTAATATGTCAGTTTCTCTTATTTTTCTATTGAGTTTTTGGATGGAGTTTTTAATGGCATATTGTACATATTTCCTCGTATTTTTCCTACAGGTTTTTGAAGACTAATCGTGGTGATAATGTTTACAAAAAAAATTGAAGCAGATATTGGGGGTGGTGTTTTTTAATACAGTACAGACACAAGCGCTTATATACACGCGCATATATTCATCCTTATGAACGCGCATATGCACACCCTACCCATATGAGCCCCTCCAAAAGACTGAGCCGACGTATCATCTTGAAATTTACGAAATAACAGTAGTCACCTCGTCATCGACGGGAACATCTCCTTCCACTGAATGCGCATCGCCGAAAATCATGAAATAAATCCAGAAATAATTGCGAGCACCAGGACTTGAATCCTGGTGAACTCGAAATACCACAGTCCCTCTAACCATCCAACTACAGACTGGTTCGCTTAAGAGGAGTGCTAGGATTAATTAATTAGAATCTCTGCTTGTGGAACCGACAGCCGGTTTCTCTCTCAAACGATTGCATCTTAATGTAACGGACGCGTAGTTCAGATCATGCCTATTGTGAACGCTGCGCGTGTGCGATGCGTCCCTCCAGGGATATAAATGTGTACATCATTGACATCAATCAATAGTGAATCGTCACAAATCTGTACATCATCGAGATCAATCAATAGTGAATCGCTACTGTTCATTCACACAGTTTCTTCACAGTTTTCTCACGTTGCGACATACGTTTTTTTTTTTGGAACTATTGCGACACACGGACTTCTTTTTGCTAGCAAAGGATAGAGAGGATTCAGAAAACCAAGGACGAGAGGCTTCGACGAGTCCGTGGGCCGGTGTGAATACGACGTGCGCGGGGGGGGGGGGGGGGGGGGGGGGGGGGGGGGGGGGGGGGGGGGGCGGAGCGGTGGGTTAGCCGGTCGAATTGCCTCTTTCTTTCCCCTCGTCCAAGCCTGGGCCTCTTTATAACCCCCGCCACCTAATCCTGCCCGTCCACCAAAGCCACCACCCCATCACCTGCCTGCCAACTAGCCCCAAATCTCCCCTCCCTCCCCCGCTCTCGAATCCCCCCGAGCATCGACCATGTCGGCCTACTGCGGAAAGTACAAGGGTACGCGATCCATCCCCTCCCTCGTCGTTCTCTCGATACGTCGGCGCTTTGATCTGCCTCGCGTGTAGATCCGTTGCCTGTTCGCCGTGCTTAGATCTACCTTTGCTCGCGAATCCGATGTAGTGTGTGCCGTCCGTACGCGCCGTGCTTTGATCTGCTTGGTTCTAGCGGTTACGCTGTTTTCGAGCGGATTTAGGCACTGCCTGTAAGATCCGAGTCCTTTGGGTGGATTTAGGTCGCGTCAGGGTGATGGGCTGCATGCTGATGATTACTGCTAGGTCCATGCTAGTGATTATTTTATATGAATGATGATACTGGCGTTCCTTCTTATTTTGCAAGTACTTTTTTTGGGTAAGTTGATGGACTCCGTTGCTCCATCTGTACATAATTCTGTTTGTGTTCGTTGTAGTGTTTACTATGGCTTTATGGTTGTTTGAAGATTGCCCCTGTAAATCTATTTCTGTTTAGATCCTAGTGGCTTCTTTTGCTAAGCTATATCCTGTGCTTATATAGTTGCAAGCTGTACTGATTGCTCTGCTGCCATGTTAATTCTTTGTTGCCCCTGGCCGTGTTAGCCACCTTGTGCTGTTGTGCAGATACATCTTTGAGAAATTGTAAAAAATTCATAATCAGTTGTCAGAATCATGCTAGTCAGTTTGTTGTGCATCTGTTGTTAGCTAAAGCTGTTAATGGCTACTCTCAACTTGTGCAGTTTTCTAGATTTTAGCACAATCTGAATAGTTATGGCAATTCATCTTAATACTGATGGTGGTTATCCTGCAGATGAGCTCATCAAGAACGCTGCCTACATTGGCACCCCCGGCAAGGGTATCCTTGCTGCCGACGAGTCCACCGGCACCATCGGCAAGCGCTTTGCCAGCATCAATGTTGAGAATGTTGAGGACAACCGCCGTGCCCTCCGTGAGCTCCTCTTCTGCACCCCTGGAGCCCTCCAGTACCTCAGTGGTGTGATCCTCTTTGAGGAGACACTGTACCAGAGCACCAAGGGTGGCAAGCCCTTCGTTGACATCCTCAAGGAGGGCAACGTCCTCCCTGGCATCAAGGTGGACAAGGGTACCGTCGAGCTCGCTGGAACCAACGGTGAGACCACCACCCAGGGCTTTGATGACCTTGGCAAGCGCTGTGCCAAGTACTACGAGGCTGGTGCCCGCTTTGCCAAGTGGCGTGCCGTCCTCAAGATTGGCCCCACCGAGCCATCACAGCTTTCCATCGACCAGAATGCTCAGGGTCTGGCTCGCTATGCCATCATCTGCCAGGAGAATGGGCTGGTGCCCATTGTTGAGCCTGAGATCCTTGTTGATGGACCTCATGACATTGACCGCTGTGCTTACGTGACTGAGATGGTCCTTGCTGCGTGCTACAAGGCCCTCAACGACCAGCATGTCCTCCTTGAGGGCACCCTCCTGAAGCCCAACATGGTCACCCCTGGATCCGACGCCAAGAAGGTGGCCCCTGAGGTGATTGCTGAGTACACCGTCCGCACCCTCCAGAGGACTGTCCCTGCTGCCGTCCCAGCCATTGTCTTCCTCTCTGGTGGACAGAGCGAGGAGGAGGCGACCCTGAACCTGAACGCCATGAACAAGCTCGAGACCAAGAAGCCATGGAACCTGTCCTTCTCCTTCGGGCGTGCCCTCCAGCAGAGCACCCTCAAGGCCTGGGCTGGCAAGGTGGAGAACGAGGAGAAGGCCAGGAAGGCGTTCCTGGTGAGGTGCAAGGCCAACTCTGAGGCCACCCTCGGCACCTACAAGGGCGATGCCACCCTCGGCGAGGGGGCCTCTGAGAGCCTCCACGTCAAGGACTACAAGTACTGATCTGCCTTCGGTGCGGTGGCCTGTTTGGGTGTCCTGCTTACCAGTATTATCGTTTGGTTTTCAGAGTCTTGATATATCCTTCGGATATCGAACCTAGTATGGAACTTGTGCTCTCAAAATAGCTTAGGTAGTTTCGTTTTTGGAGTCGAATACCCTGTTACTCGGGTGCTGCGTATCGTAAATCGGAGCGGTGGTTTTTGTTCTACTATGGTCGTGTAATTTTGCTGCTTATACAATGGGTAAAACCTGTTATATGCTGAGTACATGTCTTGAGTTGCTCTATTTCGATTGTGTTGCATCGCTTAATTTTTCTTTTGTTGCTTCAGCTGAAGCAACATCGCTTAATTTCATTCGTTGCTTCACTTGGCTG

>TaFBA12

CGAACAACATTCGGTAACCACATACAAACTTCCTTTATAACCCTAGCGTCATCGAACCTTAAGTGTGTAGACCCTACGGGTTCGGGAGACATGCAGACATGACCGAGACGTTCTCCGGTCAATAACCAACAGCGGGATCTGGATACCCATGTTGGCTCCCACATGTTCCACGATGATCTCATCGGATGAACCACGATGTCAAGGACTCAATCAATCCCGTATACAATTCCCTTTGTCTATCGGTATGTTACTTGCCCGAGATTCGATCGTCGGTATCCCGATACCTTGTTCAATCTCGTTACCGGCAAGTCTCTTTACTCGTTCCGTAACACATCATCCCGTGATCAACTCCTTGGTCACATTGTGCACATTATGATGATGTCCTACCAAGTGGGCCCAGAGATACCTCTCCGTTTACACGGAGTGACAAATCCCAATCTCGATTCGTGCCAACCCAACAGACACTTTCAGAGATACCCGTAGTGTACCTTTATAGCCACCCAGTTACGTTGTGACGTTTGGCACACCCAAAGCACTCCTACGGTATCCGGGAGTTGCACAATCTCATGGTCTAAGGAAATGATACTTGACATTAGAAAGCTTTAGCATACGAACTACATGATCTTGTGCTAGGCTTAGGATTGGGTCTTGTCCATCACATCAATCTCCTAATGATGTGATCCCATTATCAACGACATCCAATGTCCATGGTCAGGAAACCGTAACCATCTATTGATTAACGAGCTAGTCAACTAGAGGCTTACCAGGGACATGGTGTTGTCTATGTATCCACACATGTATCTGAGTTTCCTATCAATACAATTCTAGCATGGATAATAAACGATTATCATGAACAAGGAAATATAATAATAATCAATTTATTATTGCCTCTAGGGCATATTTCCAACAGTTTCTCCATAGATTGATCTTGGTGATGCGTAGAGAATTTTAATTTCTACAACGATCCTCAACACTCTTTGGCAAAGTTTGCTCGTTTAAAAGGTAGGACTATGACCTGGTTGGGCTTTGGGCCGCCTGAAGACATTGAGTTGAACAAGATTTATTGTAATCCTGTGTGATGTGAATCATACCAGTCTTACCCGCAAAAAAATAGAAATAAAAAATAGACCATACACCTATATGTTATATCAAATATATCAAACAAAATCATGAAAATGGCGATGAAATAACTGGGAATACCATGTGCAACACATATTTATGTCTGTCTTAGGTTTTTTAGGTGAATGCTTTTCGTAGTTGGTGCATAGCCTAATACTTTATTTAGGAGAACTTATTATGAAGCTTCAATTTTTTTCTTCCAAATCTAAATAAAGAAATTCCTGGTTTCCTCTATTTTCAGAAAACCCGCACCTCCCAAATAAAGCCACCGCCGAGGGCCTGGATAAAAATAACACAGGAAACCAGGGGCGGTGGGAGAGACAGCCCAACGTTCCTACGGAGGCGCACCCAGATCGGATCCCCCTTCCTTTCCCCTCGTCCAAGCCTTGGCCTCTTTATAACCCCCACCCTCTAATCATCCCCGTCCACCAAAGCCTTCAGCCTCCCTCCCTCCCTCCCCTGCCAACCCCAAATCTCCCCTCGCCCGTCGCAGCTCTCGAATCCCCCCCGAGCATCGACCATGTCGGCCTACTGCGGAAAGTACAAGGGTATGATCCGTCCTCTTCCTCCTGCCCCCTCCCCCCTTGCTCGTCGTTCTCTCGATATGCCTGCCTTCTTCGGGTGTCGATAGATCTGTTACCCGTTTTGCTCTTCTGCGTGCTTAGATCTGCCGATGCTCGCGATTCCGTGCCATGGTTTGTTAGATCCGCACTCGCGGGTTAGATCCGTACGCGCTGTGTTTGGTTCTAGCTCGTTCGAGGCGCTGGCAGTTGGATCGGGTGCCCAGGGGATCGTCGGTTGCGTCCGATGATGCGTTGCATGCTGATAATTCTGTAGTTTGCTGCTGCTGTTAGGCGCATGCTGGTGATTATTTTGTGCGAATGATTATACTGGCGTTCCTTCCTTTTCTAGTAGTTTGTGGTTAGGTTGATTTACACATCTGCACATGTTTCTGTTGGGTTCGTGGTAGTGGTAATTAATTCTGGTTTTAGTTATTGAAGGTTGCATCTGTAAATCTAGATCTGTATAGGTCCTAGGGGCATATTTTTCTAAGGAATATACCGTTCTTATAAATATAGTCAAGCTGTATGTTTTGCTCTCCTGTAATGTTGATTATTAGTTGCACTTGCCCTTATGTTTACCACCTTCCTGTTGCGCATGTGCAATGTTTTAGAAAATTGTTGAAATTTTGTACTCAATTGTCAGAATTATGTTAGTAAATTTGATTTGCATTTGCTGTTATGCTTGCTAAACATTGTGAATGGTTGCACCAACTTGCAGCTCTGTAGATTTGAGCACAATTTATAATATATTTACAGTTATGGCAATTGATTGTAATACTCTTGGTTGATTATCTTGCAGATGAGCTCATCAAGAACGCTGCCTACATCGGCACCCCTGGCAAGGGTATCCTCGCTGCTGATGAGTCCACCGGCACCATCGGCAAGCGCTTTGCCAGCATCAATGTTGAGAACGTTGAGGACAACCGTCGTGCCCTCCGTGAGCTCCTCTTCTGCACCCCTGGTGCCCTCCAGTACCTCAGCGGCGTGATCCTGTTTGAGGAGACCCTGTACCAGAGCACCAAGGGTGGCAAGCCCTTCGTCGACATCCTCAAGGCGGGCAATGTCCTCCCTGGCATCAAGGTGGACAAGGGTACCATTGAGCTTGCTGGAACCAACGGTGAGACCACCACCCAGGGCTTTGATGACCTTGGCAAGCGCTGCGCCAAGTACTATGAGGCTGGTGCCCGCTTCGCCAAGTGGCGTGCAGTCCTCAAGATCGGCGCCACTGAGCCATCGCAGCTCTCCATCGACCAGAACGCTCAGGGTCTGGCTCGCTATGCCATCATCTGCCAGGAGAATGGTCTGGTGCCCATTGTGGAGCCTGAGATCCTTGTTGATGGACCTCATGACATTGACCGCTGTGCTTACGTCACCGAGATCGTCCTTGCTGCCTGCTACAAGGCCCTCAACGACCAGCATGTCCTCCTCGAGGGCACTCTCCTGAAGCCCAACATGGTCACCCCTGGTTCTGACTCCAAGAAGGTGGCCCCTGAGGTGATTGCTGAGTACACCGTCCGCACCCTCCAGAGGACCGTCCCTGCTGCCGTCCCCGCCATTGTCTTCCTCTCTGGTGGACAGAGCGAGGAGGAGGCGACCCTGAACCTGAACGCCATGAACAAGCTCCAGACCAAGAAGCCCTGGAACCTGTCCTTCTCCTTCGGGCGTGCCCTCCAGCAGAGCACCCTCAAGGCCTGGTCCGGCAAGACGGAGAACGAGGAGAAGGCCAGGGCGGCGTTCCTGGTGAGGTGCAAGGCCAACTCCGAGGCCACCCTCGGCACCTACAAGGGCGACGCCACCCTCGCTGAGGGCGCCTCCGAGAGCCTCCACGTCAAGGACTACAAGTACTGATTGATCTGCCTTCGGTGCTGTGGCCCGTTTGGGCGTCCCGCTTACCAGTATCATTGTCATTGTTTGGTTTCCTGAGTCCTGATATATCCTCGGATATCGAACATCGTTTTTAATAATATGGAACTTGTGTT

>TaFBA13

TCAGCCTCCACGTCCCCGACACCCGCATGTCCGGGGGTGCCGGGTACTCAGCCTCGTAGAGCAGGCGCGCCTCCGCTTCTTGGAGGTGGCGGCGGCCGAAGCCGTTCGCCACGACGTCGTCGCCTGGGTATCTCTCGGCCATCTTTCTCGTCGTCGGGAAGAGGGGAGTGTTGCTATCTACGCCGGGGAGGGGGGAAAATGATAGTTGTGGCATCCACCGGCGGGGAGGTCGCCTTTTATAGCCATAGTTGGGCGGCCGCGGGCTGCATGCCGGGCGACGCGTGGCTGGACGCGCGTCGCGACGCGTTCACTGCGCCGCCCGTGAGGCATCAATGGAGGCTGACCGGCGCGGCAGCACGGCAGCCTTCGCATTGTTTCCCGCGGGAACCGAGCGATGAGGGCGACGAAGCAGCGTCTCGCTGACTCGGCGGGGCCATCCGCTTTCGCGCCAGTTTCGGCCCAAAGCGGCAAAAAAATGGGCTCCTGGGTGCGCGACTGGGCCGATTTTTGGGTGCCGGCATGGAAAAATCGCCTGGGGAGGGCCTGTTGGGGGCACGGCTGGAGATGCTCTTAGAGATCGAGGCCGCCAATGCCAAGACCAAGGAGAAAGAAGTGGCTCTCGCGAGCATGATGACCGGGGTGGAGACCATGAAGGTGGATCTCAACACAGTGTCGTCAAGGAAGATGTCGTGGTTCGAGAAGATGCAGACCGACATGCTCAAGTTCATCAACGAGCAGTGGCGGAGGCACGGGGGGGGGGGGGGGCAGCCAGGGCCCGTGCCCTCCCCAACATCCTTATTTCCTTGGTAATATGCAAATGAACAGTGACATCTTTTTACTGTTACCTGCAGCCGGCCCTCCCCAATGTCCAATTATATGCATTCCTGCCTCCGCCACTGCCGACGAGTGATCTCACGCCTTTCTTTTTCGTATGTTGGCTTGTGTGCTGGCATGACCACGGGCCGCGATGGCTGGTCAAACTCAAGTCCCACCCCTTTTGTGTGCTGGCATGTGTGTCGGCCGCTGGCGAGTGCGCCAGCGTGAACTGTGTGGTGATATTTTTTGAAGCCGACAATGTATGTCGGCGCTGGCATGGTGCCGGCATGAGACATGGCTGCTCGTATGATCTATGCGCCGCGGGCCTTTTTTTAAATTGAGTGCAGACATAAAAATGGATCGGCGCGTTGGGTGGGCGGCCGGCCAAAATGCAAAATAGGACGGACACCGGGCGGGCAACCAACCCAAACGGACAAAAAGCGGACAAATGCGCCGTATGTTTGGGTCAGCCGGTTGGAGTTGCTCTTACTGTTCATTCACACAGTTTCTTCACAGTTTTCTGACGTTGCACGTACGGGTAAATTTTTTTTTGAACTATTGGGACACACGGACGTTTTTTGCTAGCAAAGGATAGAGAGGATTCGCGAAGAAAACGAAGGACGAGAGGCTTCGACGAGTCCGTGGGCCCGTGTGAATACAACGTGCGCGGGGGGCAGAGCAGCGGGTCCGGCCAAAAGCAAGCGTTTCCCAAATCGAATTGCCCCTTTCTTTCCCCTCGTCCAAGCCTCGGCCTCTTTATAACCCCCACCACCTAATCCTGCCCGTCCACCAAAGCCGCCAGCCTCCCTCCCCTGCCTGCCAGCCCCCAAATCTCCCCTCCCTCTGTCGCTCTCGAATCTTCCCCGAGCATCGACCATGTCGGCCTACTGCGGAAAGTACAAGGGTACGCGATCCATCTCCCTCCTTCCCCCTCCTCCCCTCCCTCGTCGTTCTCTCGATACGTCGGCGGTTTGATCTGTCTCGCGTGTAGATCCGTTACCCGTTCGCCGTTCGTCATGCTTAGATCTACCTTTGCTCGCGAATCCGATGTACTGTATGCCCTCCGTACGCGCCGTGCTTTGATCTGCTTGGTTCTAGCGGTTACGCTGTTTTCGAGCGGATTTAGGCGCTGCGTTATTAGATCAGTGCTTTGGGTTCCCCGGTCGCGTCAGGTGATGGGTTGCATGCTGATGATTACTGCTAGGTCCATGCTAGTGATTATTTTGTGTGGATGATGATACTGGCGTTCCTTCTTTTTTTGCAAGTACTTTTTGGGCAAGTTGATAGACTCCGTTGCTCCATCTGTACATAATTCTGTTTGTGTTCGTTGTAGTGGTAATTATGGCTTTAATTATTGAAGATTGTACCTGTAAATCTAGATCTATTTAGATCCTGGTGGCTTCTTTTGCTAAGCAATATACTGTGCTTATATAGTTGCAAGCTGTATTGGTTTGCTCTGCTGCCATCTTAAGGGCTCCTTTGATTCAAAGGAATTTTATAGGATTTCTGGAGGATTGAAATCCTTTGGATTTTTTTGTATGTTGGTCCTTTGATTTATAGGATGGAATCCCATAGGAATTTTTTCCTATGGAATCTTTTGTACGACATTTTGGCTCCGTTTGGTTTGGAGGCAGAAAAAATGAAGGAATAAGTAACTGCACAGGAATAGAGTCGAGTGACTAGAGCGAGCCTATGGAGCCTTGTGACCTGTTCGGTGTGCAGGAAATGGAGCCCTTGGAGTACGATGAAGAGATGGGCGTTCTCACAGCTGATTAGAGTTTTTTATTGTCTCTAGACGTGATGACCTCCCATGCAGCCATTGTTTATTCGGCTGGACCACACTATAGCAAGAGCTTAGCCTCGCGTCTCGGTCAGAACATGAGGTTCCGGTGGATTTTTCGTTTCCTGTGAAAAGTACAGGTTCACGTGACTTTCCACTGGAACAGAATACCAGATTCCTGTGAAACGAACGCACTGCAGGTAGAAGTTACTAAGGAAACTGATCCTTCAAAAGTTCCTGTGAGATTCCTGCGTACCGAACGGAGCCTTTATAGGAAATCTAACATTCACTCCAACCTCTTTTTACAATTCCTTTGTTTTTCCTGTGCCATTAAACACCCATTGCTACTCCTATAGGATTCAAGTGGGCATGCCACTCTAACCCTATACTTTTCCTATTCCTACATTTTCAAAATCCTACGAATCAAAGAGGCCCTAATTCTTTGTTTCCCCTTTGTGTTAACCACCTTGTGCTGTTGTACAGATACTTCTACTGAGAAATTGTCAAAATTTCATAATCACTTGTCCGAATTATGGAATATCCTGTGCTTATATAGTCGCGAGCTGTATGAGTTTGATCTGTTGCCATGTTAATTCTTTGTTGCCCCTGCCCGTGTTAGCCACCTTGTGCTGTTGCGCAGATACATCTTTGAGAAATTGTCAAAATTTCGTAATCAGGTGTCAGAATCATGGTAGTCGGTTTGTTATACATGTGTTGTTAGTTACACTCAACTTCTGCAGTTTTGTAGATTTTAGCACAATCTGAAATACTTATGGCAATTGATCTTAAATACTGATGGTGGTTATCCTGCAGATGAGCTCATCAAGAACGCTGCCTACATTGGCACCCCTGGCAAGGGTATCCTTGCTGCCGACGAGTCCACCGGCACCATCGGCAAGCGCTTCGCCAGCATCAACGTTGAGAATGTTGAGGACAACCGTCGCGCCCTCCGTGAGCTCCTCTTCTGCACCCCTGGAGCCCTCCAGTACCTCAGCGGCGTGATCCTCTTCGAGGAGACCCTGTACCAGAGCACCAAGGGTGGCAAGCCCTTCGTCGACATCCTCAAGGAGGGCAACGTCCTCCCTGGCATCAAGGTGGACAAGGGTACCGTCGAGCTCGCTGGAACCAACGGTGAGACCACTACCCAGGGCTTTGATGACCTTGGCAAGCGCTGTGCCAAGTACTATGAGGCTGGTGCCCGCTTTGCCAAGTGGCGTGCTGTCCTCAAGATTGGCCTCACCGAGCCATCACAGCTTTCCATCGACCAGAATGCTCAGGGTCTGGCTCGTTATGCCATCATCTGCCAGGAGAATGGGCTGGTGCCCATTGTTGAGCCTGAGATCCTTGTTGATGGACCTCATGACATTGACCGCTGTGCTTATGTGACCGAGATGGTCCTTGCTGCCTGCTACAAGGCTCTCAACGACCAGCATGTCCTCCTTGAGGGTACCCTCCTGAAGCCCAACATGGTTACCCCTGGTTCCGACGCCAAGAAGGTAGCCCCTGAGGTGATTGCTGAGTACACCGTCCGCACCCTCCAGAGGACCGTCCCTGCTGCCGTCCCTGCCATTGTCTTCCTCTCCGGTGGACAGAGTGAGGAGGAGGCGACCCTGAACCTGAACGCCATGAACAAGCTCCAGACCAAGAAGCCCTGGAACCTGTCCTTCTCCTTCGGGCGTGCTCTCCAGCAGAGCACCCTCAAGGCCTGGGCTGGCAAGGCGGAGAACGAGGAGAAGGCCAGGAAGGCGTTCCTGGTGAGGTGCAAGGCCAACTCCGAGGCCACCCTCGGCACCTACAAGGGCGATGCCACCCTTGGCGAGGGCGCCTCCGAGAGCCTCCACGTCAAGGACTACAAGTACTGATCTACCTTCGGTGCTGTGGCACGTTTGGGTGTCCTGCTTACCAGTATTATCGTTTGGTTTTCAGGGTCCTGATATATCCTTCGGATATCGAACCTAGCATGGAACTTTACCTTAGGTAGTTTTGTTTTTGGAGTTGAATGAATATCCTGTTTTTGGAGTCGAATACCGTGTAACCTGAGTGCTGCGTATAGTAAGTTGGTGGTAAAACCTGTTATATGCTGAGTACACATGCTTTGAGTTTTCTATTTCGATGATTGTGCGTCGCTTAATTTTTCTTTCGTTGTTGCACTTGGCTGAAGCAACCTTCCTGTTTTCTCTTACATACTTCCTTGGGCTGGAGCAATCTTTCAATTTTCTCTCGAGCAGAAATTAAAACTTGAAATTTCAAATCAAGGTTCTGTATCCGGTTTTTAATTCTAT

>TaFBA14

AGCAGGGATGGGCCAGGCAAAAGCAGGCCCAATGCAGCGAGCGTGGATGTGCTACCTAGGGAGGCAAAAACGAATCGTTCGTTTAGTACCACATCGAACCTAACGTATAGGACGAACCGTACGGACAGACGTGAGCCTAAACCGATGGAAAACTTGACGGTTGGATAAAAAACGGACGAAATTACGGTGGTAAGAAACAATAGCCCTTTATTAATAGGTACTTCCTTCATTCCACAATGTAGTGCGCACGCGCTTCCCGAGATCCAAGTTTGACCGTAATTTAACCAACAAGACCGATTACGGCGGGAGCAAAAATTATGTCATTGAATTCATATCGAGAATATGAATTCAGTGGTATAATTTTTGCTTCCGCCGCAGTCGGTCTTGTTTGGTAAATTTATGATCAAAGTTGAATCTCGGAAAACGTGGACCCACTATATTATGAAATGGAGAGAGTATAAATTAGTATTAATGTAGTATATTAGTATTAGTATTAGTATTAGTAGACAAATATTTTAAAGTTTACATCATGTGCTTAAAAAGTACCGCGACAACACGCGGGGTATCATCTAGTAAATTATAACCGGACAGATAAAGTATCTAGTAAATGATGCCTTAAAGAAAGAAAAAGCTTGGCTTCAACCGGCGGACGCTTGCCGTGCGATCCTCCGGCTCGTTCATGTGATGCGTGTCTTGCCTGGACGGTTTCATCTTTTTCTTTCTTTCGGTCAACGTGACCATTGAGCGCTGTTCACTGTCCTCTACAATATTTTTTACAACAAGCTATATGTTGCAATAAAATGCAACAAAACTTGTATTGCAGAAATATATTGCAAAAAAAATTAAATTGCAACAAGAGCTCTATTACAAAAAAAACTATAAAAAGACATGTGCTGTAAAAAAATCTGCAACTTAACATACGTTGCAAAAAATTCTGTAACATGACTCGTGATGCAAGAAATTTCCTGCAACACGACCTGTGTTGCAACGATAAAAAGTGACGCTCGATCGCTCAATCCACTTAATCCGATGGCTCATGAGTCGACGAACCTTTTTAAAAGACTCACCGGCCTACGCATAATAGCCGTTGGATCGCCTTGGTCCATGTGTCGTCACGCTCGCACACATACTCCATTATTTCTCTTGTCAACCACGTGTCCTCTATATCTTATCCTGCGACAATGGTGGTGTTCCGAACCATAACAGCGAACGACGACTGCAACTTAGACAATCTTTTGTAACATCAACTCTGTTGTAAAAGAAAACTGCAACAAAACCTCTATTGCAAAAAAACAAATCTACAACAAAAACTCAGTTGCAAAAAAAATCCTGCAACACATTCTATATCTAAAAAATTATATTCTGCGACATAACATTTATTGTAAATATTTTTGCAACAAGATCTTTGTTGCAAAGTAAAGAGAGATATTGAGCCACATGGTTATGCCACATCCAACGATCCGCGAGGCGAAAAAATTCACCGGCCGACGCGTAGCGGGCCAGGTGGCTAAAAATCGAGTTACCCTTTTCTTTCCCCAAGCCTCCTCGGCTTCTTTATAACTCCCACCCCCTAATCTTGCCCGTCCACCAAACCGCCTCCGTCCCCTGCCTGCCAACCCCAAATTCCGCTCTCGAATCTTTCCCGAGCATCGACCATGTCGGCCTACTGCGGAAAGTACAAGGGTACGCGATCCATCTCCCTCCTTCCTCCTCCTCCTTCCCCTCCCTCGTCGTTCTCTCGATACGTCGGCGATTTGATCTGTCTCGCATGTAGATCCGTTACCTGTTCGCCGTTCGCCATGCTTAGATCTACCTTTGCTCGCGAACCCGATGTAGTGTATGCCGTCCGTATGCGCCGTGCTTTGTACATATATGCTTGGTTCTAGCGGTTACGCTGTCTTCGAGCGGATTTAGGCACTGCCTGTTATTATTAGATCGAGTGTTTTCGATTCCCCGGTCGCGTCAGGTGATGGGTTGCATGCCGATGATTCTGCAATTCCTGCTACTGCTGTAGGTCCATGCTAATGATTATCTTGTACTATGAATGATGATACTAGCGTTCACTTTTTTGGGTAAGTTGATGGACTACGTTGCTCCATCTTTACATATTTCTGTTTGTGTTCGTTGTAGTGGTAATTATGGCTATAGTTATTGAAGATTGCACCTGTAAACCTAGATCTATCTAGATCCTAGTGGGTTCTTTTGCTAAGCAATATGCTGTGCTTATATAGACTCAAGCTGTATGAGTTTGATCTGTTGCCATGTTAATTCTTTGTTGCCCCTGCCCGTGTTGACCACCTTGTGCTGTTGTACAGATACTTTATTGAGAAATTGTCAAAATTTTGTAATCACTTGTCCGAATTATGGTAGTCAGTTTGTTGTGTATTTGTTGTTTGCTAAACGTTGTGAATCGTTACACTCAACTTGTGCAGTTTTGTAGATTTTGGCACAATCTGAATTTGTTATGGCAATTCATTGTAATACTGATGCTGATGCTGTTATCCTGCAGATGAGCTCATCCAGAACGCTGCCTACATTGGCACCCCTGGCAAGGGTATCCTCGCTGCTGATGAGTCCACAGGCACCATCGGCAAGCGCTTCGCCAGCATCAATGTTGAGAACGTTGAGGACAACCGTCGTGCCCTCCGTGAGCTCCTCTTCTGCACCCCTGGTGCCCTCCAGTACCTCAGCGGTGTGATCCTCTTTGAGGAGACTCTGTACCAGAGCACCAAGGGTGGCAAGCCCTTTGTCGACATCCTCAAGGCGGGCAATGTCCTCCCCGGCATCAAGGTGGACAAGGGTACCATTGAGCTCGCTGGAACCAACGGTGAGACCACCACCCAGGGCTTTGATGACCTTGGCAAGCGCTGCGCCAAGTACTACGAGGCTGGTGCCCGCTTCGCCAAGTGGCGTGCAGTCCTCAAGATCGGCGCCACCGAGCCATCGCAGCTCTCCATCGACCAGAACGCTCAGGGTCTGGCTCGCTATGCCATCATCTGCCAGGAGAATGGTCTGGTGCCCATTGTCGAGCCCGAGATCCTTGTTGATGGACCTCATGACATTGACCGCTGTGCTTATGTCACCGAGGTCGTCCTTGCTGCCTGCTACAAGGCCCTCAACGACCAGCATGTCCTCCTCGAGGGCACCCTCCTGAAGCCCAACATGGTCACCCCTGGTTCCGACTCCAAGAAGGTGGCCCCTGAGGTGATTGCTGAGTACACTGTCCGCACCCTCCAGAGGACCGTCCCTGCTGCCGTCCCCGCCATTGTCTTCCTCTCTGGTGGACAGAGTGAGGAGGAGGCGACCCTGAACCTGAACGCCATGAACAAGCTCCAGACCAAGAAGCCATGGAACCTGTCCTTCTCCTTTGGGCGTGCCCTCCAGCAGAGCACCCTCAAGGCCTGGTCCGGCAAGACGGAGAACGAGGAGAAGGCCAGGGCGGCGTTCCTGGTGAGGTGCAAGGCCAATTCCGAGGCCACCCTCGGCACCTACAAGGGTGACGCCACCCTCGCTGAGGGCGCCTCTGAGAGCCTCCACGTCAAGGACTACAAGTACTGATCTATCTGCCTTCGGAGCTGTGGCCCGTTTGGGCGTCCCGCTTACCAGTATCATTGTTTGGTTTTCTGAGTCCTGATATATCCTCGGATATCGAACATCGTTTTTAATAATATGGAACTTGTGTTTTCATGATATCGTAGTAGTTTCTTTTTGGAGTTGAAGTACCCTGGAAATCGGGTGCTGCGTATCGTATATTGGAGCAGGTTTTTGTTCCATGGTCCTGTAATTTACCTGCTCATACAATGTTAAAACCTGTCATATGCTGAGTACACATGTGTTCCGGCACTTTTTTCTTGCTTTGTTCCTCGTGGTTTAAGCTGTCAAATGGCTATCGATAAGTTTCTACTGCCACTTCCTTCCTTGATGTCAACTCCCTCGGTTCCTAAATATAAGTCTAGACATTCC

>TaFBA15

TGGTCGCATGATGTACACGTCGTAGGGCGTAATGTGCTTTCGTGCGTGAAACTTTTCATGCACTTGCTCCCAGAAGGGCGGCCCTCTGGTTCCGCCTATGAAATCCGTGGATAAGGCCAACCACGCATCGCACGACAACTCATCCTCCATGGTCGAGTAGCTCAGCATCCTTCATACTCTAAAAAATGACATAGCATTTGAAAAATAAGCTCAATGACATTTGACCGAACTCTAGCCGGGGCGTGATGTCCGCCATGGAGGTCGTCTACAGGGGGCGGAGTGTACCTGGGTACAAATGGCTCCAGGGCTGAAAGCTACATCAAAACGACGAGCGGGCGCGTCGGTGCTAGTTGCGGACGTCCGGCAATGCCTCTATTGCGGCCAGAGACGAATAGCGGCGGCGGCGGACGTGGATGGTGCGAATGCAAAGCAAAGGGGAGAAGGGGGGAGTGGAAGGAAATGGGACCGGGAAGAGGGATTGGGTGGGCCGGGGTGTCGGAGTCCTACATTTCGGGTGCCCGGACTCCCGCAAACCTCCCACACTTTGTATCTCCAATTTGCGGGAGAAATCGTGTCTGGACCGCCCTATGAATCGATACAGGGCGGCTATGGATGACTTCCACGATCCGAACAACACGGTCCAGACGGTTGCGGAAGGTTTACGACCCACCCTGAAGATGCCCTAACAGTACAAGATAGATGCCTGATTTGATTAGGGCACGCCCCCCTTCCGTAAATGGCCCCACTTTCCTATATCACTAACACAAACCGTAAGTCGCATTGTAAGAGAAAAAAGTAGTGAATTCAGAAAAAGAAACCGAATACGACATGACGAAACATACCGCAACGCTTGCGCGTGTGATGCAACAAATATTGTGCTGGCAACATGCATGATCATGATCGTGCATGCACCCAGCAAAATCCTCACTTTAAAATAAGATCGTTTTGTTAAAGTGTTAGGTGAAATCATAAAAAGAAGAACAAAGTCATTTTTTTGCGGGTAACAGAGTCATAACTAAACCTTCACTTCGTCCATGGTTGCTTGCAGCCGAGATGCATAAACAGGCGTGCCAATCATTTGTAGCAAAATGACCAACAGAGTATATGATATATCGCATCAATGGAGACATGGCTATTTTTTTACACCCACCAGTACATTTTTTGATGTGGAATACTTTTGAATCGTAGACTTCTTTTTGTTGAAAAGATCTTTTTGGTTCATATACCATATGCACACATATGTTAATGTCAAAATGTATCAAAGAAACTATGTAAATGATCAGGGAATATCATGTCAGCACATATTTGCGGTTTTATTAGCTGGTGCACAGGCCAGTCCTAATTAAGCTTCCATTTTTCTAAATAAAGAAACTCCTGTTGTCCTCTATTTTCAGAAAACCCGCACCTCCCAAATAAAGCCACCGCCAAGGGCCTCGGTGTAAATTAGACAAGAAACCAGGGGCGGTGCGGGAGACAGCCCAACGTTCCTACGGAGGCGCACCCAGATCGGATCCGCCCTTTCTTTCCCCTCGTCCAAGCCTCGGCCTCTTTATAACCCCCACCCTCTAATCATCCCCGTCCACCAAAGCCTCCTTCCCTCCCCTGCCTGCCAACCCCCAATCTCCCGTCGCAGCCCTCGAATCTCCCCCCGAGCATCGACCATGTCGGCCTACTGCGGCAAGTACAAGGGTATGATCCACCCCCTTCCTCCTCCTCCCGCCCCCGTCGCTCGTCATTCTCTCGATATGCCTGCCTGCTTCGGGTGTAGATAGATCTGTTACCCGTTTTGATGTCCTGCTCCTGCATGCTTTAGATCTACCGATGCTCGCGATTCTATGCCATGGTTTGTTAGATCCGCACTCGCGGGTTAGATCCGTAGGCGATGTGTTTGTGTCCGCTTGGTTCTAGCGGTTCGGTTGTTTTTTCGAATCGTTCTAGGCGCTGGCAGTTGGATCGGGTGCCTAGGGGGTCATCGGTTGCGTCGTCCGATGATGCGTTGCATGCTGATGATTCTGTACTTTGCTGCTGCTGTTAAGGTGCATGCTAGCGATTTATTTTGTGCGAATGATGATGCTGGCGTTCCTTCCTTTTGAAGTAGTTTGTGGTGAAGTTGGTATACACATCTGCACATGTTTCTGTTGGGTTCGTGGTAGAGGTAATTAATTCTGGTTTTAGTTATTGAAGGTTGCATCTGTTAATCTAGATCTGTATAGGTCCTAGGGGCATCTTTTGTTAAGGAATATACCGTGCTTATATAGTGACAAGCTGTATGTTTTTCTCTCCTGTGATGTTAATTATTAGTTGCACCTGCCCTTATGGCTTATGTTTATCACCTTCCTCTTGCGCACGTGCAATTTTTTAGAAAATTGTCGAAATTTCCTAGTCAATTGTCAGAATTATGTTAGTAAATTTGATCTGCATTTGCTGTTATGGTTGCTTAACGTTGTGAATGGTTGCACTCAACTTGCAATTCTGTAGTTTTAGTTTTGAGCACAATTTATAATATCTGTACAGTTATGTCAATTGATCACAATTTATAATATATGTACAGTTATGGCAATTGATTGTAATACTGTTGGTTGATTTATCTTGCAGATGAGCTCATCAAGAACGCTGCCTACATTGGCACCCCTGGCAAGGGTATCCTTGCTGCTGATGAGTCCACCGGCACCATCGGCAAGCGCTTCGCCAGCATCAATGTTGAGAACGTTGAGGACAACCGTCGTGCCCTCCGTGAGCTCCTCTTCTGCACCCCTGGTGCCCTCCAGTACCTCAGCGGTGTGATCCTGTTTGAGGAGACCCTGTACCAGAGCACCAAGGGTGGCAAGCCCTTCGTCGACATCCTCAAGGCGGGCAATGTCCTCCCCGGCATCAAGGTGGACAAGGGCACCATCGAGCTTGCTGGAACCAACGGTGAGACCACCACCCAGGGCTTTGATGACCTTGGCAAGCGCTGTGCCAAGTACTACGAGGCTGGTGCCCGCTTCGCCAAGTGGCGTGCAGTCCTCAAGATCGGCGCCACCGAGCCATCACAGCTCTCCATCGACCAGAACGCTCAGGGTCTGGCTCGCTATGCCATCATCTGCCAGGAGAATGGTCTGGTTCCCATTGTTGAGCCAGAGATCCTTGTTGATGGACCTCATGACATTGACCGCTGTGCTTACGTCACCGAGATCGTCCTTGCTGCCTGCTACAAGGCCCTCAACGACCAGCATGTCCTCCTCGAGGGCACCCTCCTGAAGCCCAACATGGTCACCCCTGGTTCCGACTCCAAGAAGGTGGCCCCTGAGGTGATTGCTGAGTACACCGCCCGCACCCTCCAGAGGACCGTCCCTGCTGCCGTCCCCGCCATTGTCTTCCTCTCTGGTGGACAGAGCGAGGAGGAGGCGACCCTGAACCTGAACGCCATGAACAAGCTCCAGACCAAGAAGCCATGGAACCTGTCCTTCTCCTTCGGGCGTGCCCTCCAGCAGAGCACCCTCAAGGCCTGGTCCGGCAAGACGGAGAATGAGGAGAAGGCCAGGGCGGCGTTCCTGGTGAGGTGCAAGGCCAACTCTGAGGCCACCCTCGGGACCTACAAGGGCGACGCCACCCTTGCCGAGGGCGCCTCGGAGAGCCTCCATGTCAAGGACTACAAGTACTGATCTGCCCCCCGCTGGATGGTTTGGGCATTCCTGCTTACTACCTCCGTCTGAAAATAAGTGTCTGACACTTATTTTTGGACGGAGTAGTATCTGTTTGGTTTCATAGTCTTGATATTTTTAAATATCAATCCTTGTTTTTAATAATATAAGCTTCTACTTATAGTTAATTTTTGACGTTGAATCCCCTGTAATACATTTGCTGAGCAGAATGTTTTTGCTCTAAGCGGTATTGTATTTTTTCTGCTTACAATGAAATGAAATGGTGAACCCCCCCTTTACGCTGAGTAGTGTCAGTTAGTTTTTCATGAACCCTTGCAGATGCTAAATTAGCAGCAAGTTTTTGTTTATGTACTGCAAAATGTGCAATGTTTGTTTATATTGAATTGTAAAACCACTGTATGTGTTGAATCCCCTTTACTGTAATTTTGTTGATTACAGTGGTAAAAAACCCTGGTTGTGGCTTTGCGCTGTCAATGTTTGCAAGA

>TaFBA16

GCCATCATCAACTGGAACTAGAAGGCGACCTAATCTAACCTAATGGGAACAACAACCGGCCGGATTCGGAGGGTCCGAGCACCAAAAATCAACGGCGGAACATGTCGGCGGAGCAACAGGAAGACAGAGGGGGGGATGCACTCCTCGCCGCCGATTCAGAAACTAGAAGCCGACCTAAACTAACCTAATGGGAACAACAACCGACCGGATTCGGAGGCTCCGAGCACCAAAAATCAACGGCGGAAACATACCGGCGGAGCAAAATAGGAAGACAAAGGGGTGGGGAATGCCTCACCGCAGACCCAGACGCCGTCCGCCGTGAACCGAACCGCGCCACCACCAGCAACCGTCGCGGACGAGGAAGAGGGGAGCGAGAGGAGATTCGAATGCACACCGCAGCGCGAGAGGGGGGAGAAGTAATGCGGGCGAAGGGGAGAGGGGGAGTGGGGATCGTGGGTATTGGGCGAGAGGGGCGGTTTCGGCGGGGCCCACTTGTCATCAAGAGCAAAACGCTGTAACGGCTATCGCCACGCACGCTGCGCGCAGAACGGACGAGCGCGACGTTGGATTCGAATCGGACGGCAGCGCGATCGTGCGGATCCGTTGCAAACCACCAGCCGGCAGGATTTTAGCTTTGTCGTAAGAAAAACCAATGAAAACCAACACAGAAATACACATAGGAAAATAAAAGGAGAAAAATGCACGAAAGCTTGTAAAACTAGTCCAAACCGGTCATTAAAGTTCAAGGGGAGTGTATCCCTGAATGATAATTTGTTTGTGATCCTCGGATCTTTAATATTGCACTCTTTTCTTTTATAAGTTTTTTTCTAAATGGTTTCTTATGGAAGGTTTTTCAATGAGGCAATAGTAATACAAGCATTGTGATATGTCTGTTTCTCCTTATTTTTCCACTGAGTTTTTGGAAGGAATTTTTAATGGCATACTGTAATATTTCCTTATATTTTTTCTATAAGGTTTTTGGAGAAGACTAATCGTGACGAAGATGTTTACAGGAAAAATTGAAGCAGAGATAAGGAGGAGGGTTAGGATGAATTAATTAGTTTAGTTAATTAGAATCTCTGCTTGTGGAACCGACAGCCGGTTTCTCTCCCAAACGATTGCATCCTAATGTAACAGACGCGTCGTTCAGATCATGCCTCCCGTGAACGTTGCGCTTGTGCGACGCGTCCCTTCCAGGGATATAAACCTGTACATCATCGACATCAATCGTTAGTGAATCGTTACTGTTCATTCACATAGTTTCTTCACAATTTTCTCACGTTGCAACATATGGATATTTTTTTTAAACTATTGCGACCCACAGACTTTTTTTGCTAGCAAAGGATAGAGAGGATTCGCGAAGAAAACCAAGGACGAGAGGCTTCGACGAGTCCGTGGGCCCGTGTGAATACGACGTGCGCGGGGGGCAGAGCGGTGGGTCCGGCCAAAAACTTAGCCGGTCGAATTGCCCCTTTCTTTCCCGTCCTTCTTTCCCCTCGTCCAAGCCTCGGCCTCTTTATAACCCCCGCCACCTAATCCTGCCCATCCACCAAAGCAGCCAGCCTCCGTCCCCTGCCTGCCAACCCCAAATCTCCCCTCCCTCTGCCGCTCTCGAAATTCCCCGAGCATCGACCATGTCGGCCTACTGCGGAAAGTACAAGGGTACGCGATCCATCCCCCTCCTTCTTCCGCCTCTCCTCCCTCGCTCGTCGTTCTCTCAATATCTTGGCGATTTGATCTGTCTCGCGTGTAGATCCGTTGCCTGTTCGCCGTTCGCCATGCTTAGATCTACCTTTGCTCGCGAATCCGATGTAGTGTATGCCGTCCGTACTCGCCGTGCTTTGATCTGCTTGGTTCTAGCGGATACGCTGTTTTCGAGCGGATTTAGGCGCTGCCTGTTAGATCGAGTTCTTTGGATTACCCAGTCGCGTCAGGGTGATGGGCTGCATGCTGATGATTACTGCTAGGTCCATGCTAGTGATTATTTTGTATGGATGATGATACTAGCGTTCTTTCTTATTTTGCGAGTACTTTTTCGGGTAAGTTGATGGACTCCGTTGCTCCATCTGTACATAATTCTGCTTGTGTTCGTTGTAGTGGTAATTATGGCTTTAATTACTGAAGATTGCACCTGTAAATCTAGATCTATGTTGATCTTGGTGGCTTCTTTTGCTAAGCAATATCCTGTGCTTATATAGTTGCAAGCTGTATTGGTTTGCTCTGCTGCCTTGTTAATTCTTTGTTGCCCCTTTGTGTTAACCACCTTGTGCTGTTGTACAGAAACTTTATTGAGAAATTGTCAGAATTTCATAATCACTTGTCCGAATTATGGTAGTTAGTTTGTTAAGCAATATCATGTGCTTATATAGTCAAGCTGTATGAGTTTGATCTGTTGCCATGTTAATTCTTTGTTGCCCCTGCCCGTGTTAGCCACCCTGTGCTGTTGCGCAGATACATCTTTGACAAATTGTCAAAATTTCATAATCAGTTGTCAGAATCATGGTAGTCAGTTTGTTGTGCATGTGTTGTTAGTTACACTCAACTTCTGCAGTTTTGTAGATTTTAGCACAATCTGAAATAGTTATGGCAATTGATCTTAAATACTGATTTATCCTGCAGATGAGCTCATCAAGAACGCTGCCTACATTGGCACCCCTGGCAAGGGTATCCTTGCTGCCGACGAGTCCACCGGCACCATTGGTAAGCGCTTTGCCAGCATCAATGTTGAGAATGTTGAGGACAACCGTCGCGCCCTCCGTGAGCTCCTCTTCTGCACCCCTGGAGCCCTCCAGTACCTCAGCGGTGTGATCCTCTTCGAGGAGACCTTGTACCAGAGCACCAAGGGTGGCAAGCCCTTCGTCGACATCCTCAAGGAGGGCAACGTCCTCCCTGGCATCAAGGTGGACAAGGGTACCGTCGAGCTCGCTGGAACCAACGGTGAGACCACCACCCAGGGCTTTGATGACCTTGGCAAGCGCTGCGCCAAGTACTATGAGGCTGGTGCCCGCTTTGCCAAGTGGCGTGCCGTCCTGAAGATTGGCCCTACCGAGCCATCACAGCTTTCCATCAACCAGAATGCTCAGGGTCTGGCTCGTTATGCCATCATCTGCCAGGAGAATGGTCTGGTGCCCATTGTAGAGCCTGAGATCCTTGTTGATGGACCTCATGACATTGACCGCTGTGCTTATGTGACCGAGATGGTCCTTGCTGCCTGCTACAAGGCTCTCAACGACCAGCATGTCCTCCTTGAGGGTACCCTCCTGAAGCCCAACATGGTTACCCCTGGTTCCGATGCCAAGAAGGTAGCCCCTGAGGTCATTGCTGAGTACACCGTCCGCACCCTCCAGAGGACCGTCCCTGCTGCTGTCCCTGCCATTGTCTTCCTCTCCGGTGGACAGAGCGAGGAGGAGGCGACCCTGAACCTGAACGCCATGAACAAGCTCCAGACCAAGAAGCCCTGGAACCTGTCCTTCTCCTTCGGGCGTGCCCTCCAGCAGAGCACCCTCAAGGCCTGGGCTGGTAAGGCGGAGAACGAGGAGAAGGCCAGGAAGGCGTTCCTGGTGAGGTGCAAGGCCAACTCTGAGGCCACCCTCGGCACCTACAAGGGCGATGCCACCCTCGGCGAGGGGGCCTCTGAGAGCCTCCACGTCAAGGACTACAAGTACTGATCTGCCTTCGGTGTGGTGGCCCGTTTGGGTGTCCTGCTTACCAGTATTATTGTTTGGTTTTCAGAGTCTTGATATATCCTTCGGATATCGAACCTAGTATGGAACTTGTGCTCTCAAAATAGCGTAGGTAGTTTCGTTTTTAGAGTCGAATACCCTGTTACTCGGGTGCTGCGTATCGTAAATTGGAGGGGTGGCTTTTGTTCTATGGTCGTGTAATTTTGCTGCTTACAATGGTAAAACCTGTTATATGCTGAGTACATGTCTATTTTGATTGTGTTGCATCGCTTAATTTTTCTTT

>TaFBA17

ACTGCTGACGCCCCTGCGCTTGGGGACCAACACATCTGCTTCGCCATGAAGACGTGATTCGTGTCATACAGTCTACGGACAAGGAGGAGCTCCTGCAGCGGTTCAGGTATAGATGTGCATCTCATTATTTTTTTACCTCATATGAGGGCAGGTACGGTTCCTTTTTATGCTATTTGTTGTGAGTGCGTTGCAGGTTCACGTAAACTGAATGGGCTAATCTCAGAGTTCCTCCATCAAATAAACTGGATTGGCTAATCCAGAGTGCTTGCCCGTAAAGTGTATGCCTCCTTTCTAATTTTTTTATATTACTTCAACAATAAAAGGTACAGAAAAACCATATAAAGTACTTCATCTGTTCCTAAATATAAGTTCTTTTAGAGATTTCAATATGGACTACATACAGATGTAACATTCAATCATTTGCTCCGTATGTAGTTCATACTGGAATCTCTAAAAAGACTTATATTTACGAAGGGAGGGAGTAACACTTTGTAGATGCGTGAAACATGTACAATCGATTTTCTCTGAGGAGAAAAAGACAATTCATCAGCTAGATATATCAAAGGACAGTCTATGTCAATTGAACTAGCACTTGAATTTTAGCAAATCCAAGTTTTTTTTCTACAACTGCAATTCGGTATTTGGGTGGGCTATGATATGACTCAGTTTATTAGCTTCAACGGATTTTAAGTGACAACACGCTGATGAATAATAGAAACACCACGCTGTCCATATGGGTGGACTATGGATGGATGGCGCCTACAAACACCACGCTGCAGGCCTGCAGCTCGACCTACATTTTTTTTTCTTCTCTCCCTCAGGCTTGAATGTCCGCGGTCAATCTCTGCCGCTGGTGGTGCAAGTATGATAAATGGGTGCATTGATCTCTTTTTTGTTGTAGTGCAACACCTTGGAGATGCACATAATTTGACATGACAGCCTCTTTTTCTACTATCAATGATGTTTGCTGCAGAGATTTTACAGTTGGGAGAAGATAGCAAGGAAAAAAAAAGAGAGCGATTTAATTGTAAAAAGAAAGTCCCCAATGCAGCTCGTTGCAACGCACGGGCATTCTACTAGTTTCTGTTGGGTTCGTGGTAGTGGTAATTTTCTGGCTTTAGTTACTCAAGATTGCATCTGTAAATCTAGTTCTGTATAGATCCTAGGGGCATCTGTTGCTTAGCAATATCCCATGCTCATCTATTCGCGAGCTGTTGCCATGTTAATTCTTCGTTGCCTCTGCCCTTATGTTTGCCACCTTGTGCTGTTGTGGAGATGCAATTTCGGATCTTTCTAAAGAATTTCCTAAGGTCAATTATCTAAGTCTGGTTGTGTATGTGCTGTTAGCTAAAATATTGTGATAAGTTACACTCATCTTGTGCAGTTTACCTCGTGGAGATGGGATTTCTGTAAAAATTGACAGAATTATGATAGCCAATTTGTCATGTATGTTAGTTATACCATTAACGTTGTGAACGGTTCCACTCAACTTGTACAGTTGATCTTAATTTACAGGATCTGAATAGTCATGGCGGGCAATGTCCTTCGTCGACATCCTCAAGGCGGGCAATGTCCTCCCTGGCATCAAGGTGGACAAGGGTACCATCGAGCTCGCTCGAACCAACGGTGAGACCACCACCCAGGGCTTTGATGACCTTGCATTGGCAAGCACTGCGCCAAGTACTACGAGGCTGGTGCCCGCTTCGCCAAGTGGCGTGCAGTCCTCAAGATCGGCGCCACTGAGCCATCGCAGCTCTCCATCGACCAGAACGCTCAGGGTCTGGCCCATTGTTGAGCCTGAGATCCTTGTTGATGGTCCTCATGACATTGACCGCTGTGCTTAGGTGACCGAGATCGTCCTTGCTGCCTGCTACAAGGCCCTCAATGACCAGCATGTCCTCCTCGAGGGCACCCTCCTGAAGCCCAACATGGTCACCCCTGGATCCGACGCCAAGAAAGTGGCCCCTGAGGTCATTGCTGAGTACACCGTCCGCACCCTCCAGAGGACTGTCCCTGCTGCCGTCCCTGCCATTGTCTTCCTCTCTGGTGGACAGAGTGTGGAGGAGGCGACCCTGAACCTGAATGCCATGAACAAGCTCCAGACCAAGAAGCCCTGGAACCTGTCCTTCTCCTTCGGGCGTGCCCTCCAGCAGAGCACCCTGAAGACCTGGTCCGGTAAGGCAGAGAACGAGGAGAAGGCCAGGGCGGCGTTCCTGGTGAGGTGCAAGGCCAACTCCGAGGCCACCCTCGGCACCTACAAGGGCGTCGCCACCCTCGGCGAGGGCGCCTCGGAGAGCCTCCATGTCACGGACTATAGAAGCATGGTGGGACCAGTCTGAAAATTAGTGGGAGCAAACCGACATAAGAGATAAGACTATAGCTATATTCTATTTTCTTACGAAAGAAGTGTCTTCTCAGCTCTTTTTCCTCATACAAATTCCATCTTTTTGTTGAAACTGACATATTTTGGCACGGAAATGAATTCTTTATTTTCTT

>TaFBA18

AAGCCTAAGCCCGGATTGGCCCAGCCCAGCCATCAGCCACAAAAATCTAGGCCCAGGCCCGGCCCATGGGCAAGGTCAGGCTGTGTCACATCGGGCTTTTTGGGGCTAGGTATACTCTTAGAGCATCTCTAGCAGACCCTGTATAATATCGACCCGTAAAATGCGTTTATAGTTCGCTGAAAAACGGATTTACGGGCCGACGCGGGGTCTGCTCGCCCGCAGCCCGCGTTGACCCGCAAACCGAAAAGACTCGATAAGCGAATTTGACAGCGGTTTCGACAATTGAGTTCAAATTCGAACAATAAAACATAGTTCGCGCGCAAATAAAAGCATAATTTTGTACGACAAATGCAAAAGGACTTCATGCAAAAGCGATGACCACGTCCATCATCATCTTAGTTGCTTTTCACCCGTCGTCATGATCTTCACTCCGTACCACCTCCATCGATGCCATTGCCGCCCCGGAATCCATCGCCGACACTTTTCCAACTCCGACACCAAAAGCATCGCCGGGCGCACTGAATGCATCAGCGGCACTAGTTCCAAACCCGAAGAGAAAACAATGCCGGCACTGAAACACGCACCGGCCGCCGCTAGTATCCTCCTCTTCAAGATGTCTCTTCTTGCCATATCATGTCATTCTTTGGTGATGTCGTTCATGTCATCGTGGTTCGGACCACCGCTACAATGAGTGACATCAAGTCACGAGCTACCACAATGGCGAAAAGCAAAGCAAGTAGAAGGGATCGAAGATGCATACCTTCCGGCCATTTCATCGAACACCTCGCCTGCGTTGGAAGCATCGTCATTGAACGCCATTGTCGGGGAACCTCTTGCTGGGGGCGGAGGGAGTGGATGAAGGCGCTGACCTTTTCGTAGGAACCTTCTTGCGCTTCATGCCTGAAACCTTGCCTACCTTCTCCGGTGGCGGTCGGGCAGTTCGCGCAGCGGCGGCGGCGCCCGACGCGCGTGCCTTCCTGGCAGGGCCAACCAGATTTCCGCCCTGCACGACAACGTTGCCCTGCCGCTTGCGGGCAGACACGGTGGTGCCTTGAGCGACGGTGGGGCAACATGACCGACGATGAGATCTGCCGGAGGGGATTGTGCATGCGCGACCTCCACGGTGACGGGGGACGGCTGGGAGGCTTCCATGGGGGCGAAGAATGGCCGAAAAAGATGGATCGGAGAGGGCCATGGAGGGGCAATGGTGGCGGGGGGGGGGGGGGGGGAAGAGGAAGCGGGAATGCCGGCACGGAAATTTCCCTAGCGCCAAATCTCGCTGCGGATAGGGGGGTGAGCTCATGTCGGCCTCCCACCCCGTAAATCTAAAGGTTGATGGCAAATTTTTGCCGTGCCCGCCAAATTTTTTTACGAGTCGGACACGTTTAGGAGTGTGATCGGGCAGTATTTTTCGCACGGACCCGTATTTTAGCGGTTATTTTGCGGGTCGCGGCATTATAGGGGCGGTAGGATGCTCTTATGCCCACTCCCTCCATTTCCCGCCCTTTTTTTTTCCTTCCCCGCTCACTCGAGAACCTTCCTTCCTTCATCGGCTGGCTCCGCCACACACCGCCACCGCCGGCGAAGAGAGAGAGAGAGAGAGCGCAAGGATGTGGGAGTGGCCGCTGTCTCGCTGCTACTAGCTCCTCGTCGGCAAAGGTGACCGCCACGACCTCCTACTGAACCTTCCGCCTGCAGCCGCACCAACCCCTCTCCCCCGCGCCGTCCGTCTCAGCGTTGGATTCGCTTGTTGTTGTGCAGACCACGACCGGCGGCCGCCGGCGGCCAGGCTCATCGAAGCCGCTCACGACGGCGACGTGCGCAAGATCAGTAAGGGCCGCACTGAACCCTCCCCCCTCCCCCATCTCTCCCTCCCTCCGGCGTCCATCTGCTCGCTAGATCTGGCCGTGTTTTGCTACCGTGTGCGCCTCGCCTCCAATCGGTAGACACTGGGGCTTAATTAATTCCCCAGGTTCCTTGGGGGAATAGGCTTTCTCTTGTGATTTAGGTTGGAATGCGATGCTGGGGGCTGAATTAATCCCTCGGGTTCCTTGGGGATGTCAATTTTTGCTTGTGATTTATGTTGGAATGAGATGCTTGGGAATCTCTCGCTGCAGTTCTATTGGGGATTTCTGAACCCCCTTCTTAAGGATCAGTGGCTATTTCTTCCTGATTTGGGCCGGGCTAGTACATTATTGCCATGATTGATGATGATTCATGTCCTAACTAACAGTGCCTCCCCTTTTTGTTGTGTGGAATCAGGAGATTGCAAAGGAGCTGAATGTGCACGGACATGGGATCCCGGTGACAGTGGCCAACACGACCTTCCATGGCATGAATGCGCTGCATGCCGCCGGTGGCCTAGGCTGGCTGCCAGTCTGCCGGCATCTCGCCGAGGAGGTCAAGATGGTGGAGATCGACAAGCCTGACATCTGTCGGGGTAATTCGTTCGTGCCTCTCCGGTCCTAAACTGTATATACAGTAGTTTCTATGGCTTGATTATCTACTTCACCTGCATCATAATACTAGGACATACACCTGTGGAGCATGCCCTTACCGACGGCCACCTTCCTGCCGTCAGGTACCTTCTTGTCGTGGAGCTGATCTGCCCCAGCGATGTAAAGATGGGACCATGTCTACTCTTCTTCATTCAGCTGCAGTTCATGCTGTCAAAAGGATCTTAATTTGGTTTTTAATTCCCTGTGCTGTAAGAATATGCCATAGTTTCATGCTCACAGTCTTGACTACTCTCCTGCTCCCTGCTGGACCTTTTTCTTAATGAGAATTGTTTTGTGCTCGATTTTATTTTACCAAGTGCTCAGCTAACCTAATATAAGTAAAACAAACCTGATCTTTTTCGCTTTTAATGTCCATCAGCGTCTGAACAGTGGTCATTCGTATGATAAGTTTAAACATAGTCGTCACATTAATTTGCATTTCCTGCTTCACGATATGTAAAATAGCCATCTGTCATGTATTGCTACAATTCTTGTTAGGCATTATATTTATCCTTAGTCTGGATGTTTCAGATGTTATTTTTGTGTTAGCCATCTAATCTAGTTTCCCAAGAAATGTCTGCTTAATTAATATGTGATATGCAAGTTTCTCCGGGTATCTATTCATACATTATTTTTTGTATGATTACCATCTTCTTATGGTAGCTGTTTATGGTAATTTGTTCTGTATGTCTGTTCAGGGTAATGTGAACGAACAATTGCTTTCCATCAATAGTTATTGAAACGATTTTGCCCAAAACTGTTCTGCTCATATGCTGTTATGATCTTAAATTTGTTTTCTCCTTTTCTTCAGGGCACTCTGTAATAGTAAAGTTTCTTCTTTCTAGAGGAGCTGATATTGATGCGCTATCTGTTTTTGGGAGACCACTCTCCCTTGCTGTTTTCAGAGGACATGCTAGCACGGTCAAGATCCTTTTGCAGCACAATGCAGATGTAATATTCAAAATCTTCGTTGATCTGTTGTCATTTAATTCCAATTCTTTCTCTTGCTGGCATTTTGTTACACTCTCCAATTGTTCCAACATGCATGTAGTTGATGAGATTATTGGTTGGTGACTAATCATGCAAGTGTGACTTAGTTACCGTTTGGTTTGCAGGTTGCAGGCACGGTTCTGGCAAGATTCCTTTTCTACGACTTAATTGACATACAAAGTTGTGGCAAATATAATCAACCTTCGAAAACCAACCAAACAGGCCTTGCTATAGCTATTAATGTGTGAGCAAACCGTATTAGAGTGTTTTTCAAGTGATTATTTTGTGCGAATGATGATACTAGCATTCCTTCCTTTTACACATCTGCACATATTTCTGTTGGGTTCGTGGTAGTGGTAATTCTGGCTTTAGTTACTCAAGATTGCATCTGTAAATCTAGTTCTGTATAGATCCTAGGGGCATCTGTTGCTTAGCAATATCCCATGCTCATCTATTCGCAAGCTGTTGCTGCCCTTATGTTGCCACCTTGTGCTGTTGTGAAGATGCAATTTCGGATCTTTCTAAAGAATTTCCTAAGGTCAATTATCTAAGTCTGGTTGTGTATGTGTTGTTAGGTAAAATATTGTGATAAGTTACACTCATCTTGTGCAGTTTACCTCGTGGAGATGGAATTTCTGTAAAAATTGTCAGAATTATGATAGCCAATTTGTCATGTATGTTAGTTATACCATAAAAGTTGTGAACGGTTCCACTCAACTTGTACAGTTGATCTTAATTTACAGGATCTGAATAGTCATGGCGATTGATTGCAATCTGATGTTCATTGTCTTGCAGATGAGTTCATCAAGAACGCTGCCTACATTGGCACCCCTGGCAAGGGTATCCTCGCTGCTGATGAGTCCACCGGCACCATCGACAAGCGCTTCGCCAGCATCAATGTTGAGAACGTTGAGGACAACCGTCGCGCCGTCCGTGAGCTCCTCTTCTGCACACCTGGTGCCCTCCAGTAGCTCAGCGCTGTGATTCTCTTTGAGGAAACCCTGTACCAGAGCACCAAGGGTGGCAAGCCCTTCGTCGACATCCTCAAGGCGGGCAATGTCCTCCCTGGCATCAAGGTGGACAAGGGTACCATAGAGCTCGCTGGAACCAACGGTGAGACCACCACCCAGGGCTTTGATGACCTTGGCAAGCGCTGCGCCAAGTACTATGAAACTGGTGCCCGCTTCGCCAAGTGGCGTGCAGTCCTCAAGATCGGCGCCACTAAGCCATCGCAGCTCTCCATCGACCAGAACGCTCAGGGTCTGGCTCGCTGTGCCATCATCTGCCAGGAGAATGGTCTGGTGCCCATTGTTGAGCCTGAGATCCTTGTTGATGGTCCTCATGACATTGACCGCTGTGCTTATGTGACCGAGATCGTCCTTGCTGCCTGCTACAAGGCCCTCAACGACCAGCATGTCCTCCTCGAGGGCACCCTCCTGAAGCCCACCCCTGGATCCGACGCCAAAAAGGTGGCCCCTGAGGTCATTGCTGAGTACACCGTCCGCGCCCTCCAGAGGACTGTCCCTGCTGCCGTCCCCGCCATTGTCTTCCTCTCTGGTGGACAGAGTGAGGAGGAGGCGACCCTGAACCTGAATGCCATGAACAAGCTCCAGACCAAGAAGCCCTGGAACCTGTCCTCCTCCTTCGGGCGTGCCCTCCAGCAGAGCACCCTGAAGGCCTGGTCCGGCAAGACAGAGAACGATGAGAGGGCCAGGGCGGCGTTCCTGGTGAGGTGCAAGGCCAACTCCGAGGCCACCCTTGGCACCTACAAGGGTGGCGCCACCCTCGGAGAGGGTGCCTCGGAGAGCCTTCACGTCAAGGACTATAGAAGCATGGTGGGACCAGTCAGAAAATTAGTGGGAGCAAACCGACATAAGACTATAGCTATATTCTATTTTCTTATGAAAGAAGTATCTTCTCAGCTCTTTTTCCTTATACAAATTCCATCTTTTTGTTGAAACTGACATACTTTGGCATGAAAATGAAGTTCTTTATTTTCTTTTCCAGCCCAACAAGCTAGTTGTCCGTTTGGACCTTTAAGTACGGCATTACAAAAATCTTCTGTATCATGTGTGAAGCTATTGATTCAGGTATAGTATCCTCTGCTTATATTGATTCATCTGTTTGGATTTCTATAGACTGATGTTTGATTTAGCTATCTTTGTATTACCTACTTGAAAACCATATGATTGGCTGGTCTTCTATACTTGCATCTGATAGAGTGCAGCTACTGTCGGTGGTGATAATCTTTTGGAAAAGGTTGCAGAGAAGGGCTTAACTGAAGCTATCAAGTGCTTGTTGGAAGCTGTTGCAAACCCAAACGTTCCTAACACAGTAAGAATGTTCTTTTTGTCACATTCTTGTGTCTTGCTTGGCTTTGTAATTTCAGTAGACTTGTCTTATACAGATATGCTACAGTCGTATGAGTTATGATGTCAGTACAACTACACGATGCTATCTGTAAAATTAAAATAAAAGGCTGCTAAGTTACCTTTTTTTTTTGCATACACTTGCTACTGTTTTGCCATGTCCCCTTCCTGCCACGTCTTTAATTTGGTCACTACATCGACCTATATATGCCATGCGCTAGCACTACATCGACCTATCATGATGGTAGCGAATCAATATGGTTTGCATATCATGTCTAGAACATTGCTTATTTCTTGATGTTGACTATTGCTTTTTTAAAGCCCTCATTGTTCCTTAACAGTTTGGTAGATTGCCAATAGAGTTGGCCGCTCAACTGTGAAAAATTGAAGCGTTGATGGAATCATTAGTCATGTGGAGATGGAAATCAAACAGCTGAGGTATGACACTGTAGCATATTTTGATGCACACCTACGGGAACTTCCCCGTCACGGCAATCACCTAGCAGGGCAGGGCCACGTCGCCGGCATAACATGTTTAGTCGTATTTCGTCGTAGATCATAACTAGTCGATCTATGTGTGTTTCGTGAACTTGCTATGTCCGGTTGGAGGTACTCTAACCTGTTTGTGTATGGAGGTGTTGACAATGGAGTATATATATACTAGTTGATTGTAACATAATGAAGTACTCCCTCCGTCTGGAAAAGCATGTCCCACTGGCATTTTTGCAAAAAAAAAACATGGTCTTTTTGGGAAACAACCCACGGTCCAGCCACACTGTCTGTCTGATACTGCTGTCGCTCACTGGCTCTCGCCCCCGCCGTTTTGCTCCGGCTCGCCGCCGTCGCGCTCTGCTTCAGGCCGCTGCTGCCGTGCACTGCTTTAGGTCGCCGCCGCCGCGCTATGCTCCGGCTCTCCGCTGCCGCGCTCTGCTTCGGGTATTCGCCGCCGCGCGCTGTGCTCCGTCTCGCCACCAAAAACGCTCTGCTCTCGATCCAAAGAAGCCGACGAGTCACCGGATACGGCGTCGGCGAGGCTGTATATGTTGTCGGCGAGGCCGTACGCGCCGGATCCCGACCACCTTGCTACTAAAAGCTTCCCTTGCGAGCTCAAATGAGTACGGCGGCAGCGATGTCATGGAGCAGCACGGGTTGCCTTTTTTCGATCTGGCCCTGCCCGTCCGACGGCGGCGTCCTCCCTGCCGTCTTCCTCGCCCGCACAAGCCGCCAGTATCCTCCGTAGGTATGCTGTCCAGTAGATGGACGGATTGATGGGCCGCTCGTATCCTGCAGGGGAGGAGGTTGCTCTTGGTCACTAGAGGAGCACAGCAGGGGGTTTTTTCACAGTGGAGGCTGTCAGTTAAACTCGAGCTCGATCTTAGGCGGCACATGAGCCGCCTGTTTCCCATCCAAACTTTCTGTCCATGGATATTGATGGAGCGAGCATCTTCATACCCGGATCATGCTCATTGATTCTCAAAAGAGCAGTATGTAGATGCAATGGAGAGAATAGCTGTTGGTACTGTGATGGATATGACTATTGCTGTTTTCAGTAAGGAAATGCATTGTAAGCAATTCTAGTCTGAAACTAGTAGAGCTCTTGGTGATTTACAAATTGCAAGCAAGATGTCTGAAACTAGTAGAGCAAGTGTACTGCTTATTGTAAGATGTCTGAAACTAGTAGAGCAAGTGTAGATGTCTGTAAAAATTCAGTATTGTATCCTGCTGTGAATATAAAGTGTACTGCTTATTGCAAGCAAGTTTAAAAATTCAGTCTAAAAGTCAGTTAACTGAATTTTGACACAGTAGTTAACTGAATTTACTGCCATCTTTACCAAATGCAGTTTACCCATTTGTTGCTTCTCACTTTCTGCAATGTTTGCTAAGATGATTTTTAGTGAAGATGATCTTAGTGGTGATACCTTTTCATTTACACAGATCATTTACACAAACCATGACGTGATCTCAAGCAGTTCTCAGTTCAGCAAGACCAAGTCAGTGGCTCCAGCCCCAGCAAGATCAACATCCCTGGTAAACCTGCTATCTTGAGTAAGTGACGTTCACTTTGTACTCCATGTAACAGGAAAAATGATTACTACTGAGTGAGCAACATCAACAGTGTGTAAGCATTCACAAAAGAAAAAGAGAGTGTGTATGCATATTATTTTCGTATAATGTGTAACTATTTGGTCAATATTTACTTTGATACACATGAAAATATTTAGGAAGATGTTGATAGAGTTCGCATGGAATGATATTTAGTTAGGTTATAGTCAGGTCAATGCAAACATGCCCAAGTGATATTCAGTATACTATAATAGATTAATACAGTATACAATTATGTGCAAGAACTCTAGGATCAATATTCAGTTTAGGAGAAGAATCTTCAAGGACAAACGTGGCATAAATGGTTATGTGCTCCTACTCCACTTTATGAGTATTTTTCTAAACTGCCAAAGACAATTTACTGAAAATGCTAGTAGTATCTCTCACAAGTTAACTTAACTTTGTTCTAATTACCTGAATTTAAACTACAGCTGTACAACAATGAAGCAAATAATCTATTTCATTTAAACTACAGTTGTACAACAATGAAGCAAATAATCTACTGCAATTTGATCTACTGCAAATTACCGAATCAATTGATAGCTACCAATTTGATCTTCATTTGCATAAACCTCGTTGGTGCTCGCACTATTTACTGAAAATAAACCATTATTTACTGAAGATAAACCTGGTTGATCTTCATTTGCATAAACCTGGTTGAGTATTACAGAGCAATTGATAGCAAATTGACCTCGTTAGAATTGAGACAGCAACAGTAACAGTAACAGTTAACTGAACTCTTGTTTTGTAACATTTCTTCAGAGGACATGATCAATTATGTTTCAAACTACAGAGCAAGCAAGGTCCGGCACTGGAGATGAAAAAGCAAGGAGCCCCGTAACTTCTTCTCACGTACAGCAGCACGAGCAGGGGACCTCCGGTGACAGCACACGAGTAGGGGACCTCCGGGCGGCAACAGGAGCAGGGGAGCAGCAGTAACTTGCAGAGGAGCAGCAGAAGGAGCTCGAGCTTGATTTCCAGGACGAGCTTGAGGGAGCTGAGGTCCAGGCGCGCGCAGGTCCTGGGCGTCGGCGGCGGCGCAGATCCTGGGTGGCAGGAGGCGTGCGCGACCCAGGTCCTGGGTGGCGGAGGAGCACGTCCTGGGCGATGGCGGAGCAAGCAATTGGGTGGCGCAGGTCCTGGGCGGCGTTGGCGGCGGGGGAGCAAGCAATTGGGCGTCGGAGGAGCACGTCCTGGGTGGCGCCAGCGTAGGTCTTGGGTGCCGACTGGCCCTGGCGACGGGCGGAGGAAGAACGGCAACCTGTTTGTAACAGATTTAAAGCTACGAGAGCGTTTTCGCAAAATTCGCAGTACAATAAGCTGGAGACAAGCTTTTCGGGATGGAGTGTAGTACTCAGACGTTACGATATGTAGGCAAACATGTTTGTCAGCAACATACATACAGTTTGTATTCTTTGACTGCATCGGCAGTCATGTACTTACAAGTAAAGAATAGAATCAAGGGGAATAAAGGGCATCCATCGCTCAGGAATAAGCAATTCATACTCCT

>TaFBA19

GGCCCGCGATCCAGGGACACGGAGCGGCTGGGTAAAAAGCCTAGAGCAGTGGAGTCCTCGTGTGTCACTCCTCCTGACTTGCTCACTCCCCCGTCCGCTTCCGCTCTGCTCGCCTCTGCTTCGAGCTGGTCGTCGCCGTCGGCGAGCGGGATCACACGCCACTCGCGGGATGTCGCCTTCGTCCTCGGCGGCGAAGACCGTCGCCTTCGTCGGCGCCGACGGGCTGAGCGCCGCGCTCGCCGCTTCTTTCGCCCGCTCCGGCGCGATCGTCCGCTTCTACATCGACCGCAAGGTCGCCTTTGCTCGATCGCCCTTCCCTCGTTTCCCCGTTCCTTCCACCCCCGTTTTGGTTTTGCTTCCTCTCGGAGGTGTTTTCGCGGGACGCTCTGTCCTGACGGTGCGTGTCCGTGCAGGCGGATGGATCGGCGGCGACGGCGCTCGCGGAGCAGGGCGGCGGCGTCCGATGCGTGAGCCCCGCTGAAGCCACGCGAGGTGAGCGTATATAACGGTCTCCTGCACTGGACCGGTTCCAGATATGGCCTTCTCGCGGCGCGCGTGAATGGCATGCGTGCGTACGAGCCGTTTGTTGGAATGCCGAAGAGACCATGCTCCTACGTGCTGAAGAATGCTTCTTGCCGTGTGTGTGTGTGTGTTGTTTCTATCTGACCTATCAAAAACTCCTGGCGCCTGCAGATTCTGCGCTAGTCGTTGTGCTCAGTGACGCCGATGGTGTAGATGAGTTGTTCTTTGGAGCTCAGGGTATAGTGCAAGGTATGTACCATCTGTGTGCTAGTGTTAGTGTGTTGCCTGCCAGTCTGTCAATCTCATGCACCTGTTTCTGTACAAGTTATATATACACTTGACATTTGATATGATGTTTGCTCGAGCATTTCATAGGATTGTGCAAAGAGGCCGTTGTATTAATTCGGTCGACGTTGGTGCCTAGCCATCTTGAGAAGTTGGGGCAGAAGCTTGCAGGTATAATGCTAATTTGTGCATATATTGTCTTAGCTGGGAACAGTAGCTGCTCTGCTTACAATTTTATGTGCACTTTTAAGAATTAGAAAGTGCACATTTAACAATTGTAAGGCCCTCCTTGGATGACTGGAATTAAAGAACATAGGAATAGGAATAAACATATGATTGGAATTTCATGTACTACTATAGTTTCCTACAGGATTTTTTGGTTCACAGGATGTTGGTTTTTGCAGATGAGAAGAAGGGTATCTTCCTTCTTGATGCTTACATTTTTAGTGGTTTATCTGATGAACTGAAGCAAAATATTGTCGTAAGTGATACACAGCTGAGAATCTGAAGTGTGTGCTTGTGTGCTTACGTTATTCTGTTATCACAATTTAGCTTTTGAATGTCAGGTTGTTGCATCTGGGAGAAAGGATGTAGCAGAAAGAGCTGGGCAATTCTTCTCCGGTAGTTCCAAATTTGTGTACCTTACTAATATTTTCTTCCTGGTCCTAATCTTATGATGCTGAAATTTTAAGCTATCTGACATTCCAGATCTTGACAAAACCAATTACTTTGTCGAAGGTGAATTTGGCTGCAGCAGGTTGCTTTCTTTCCTGAAAATAGCAAATATAAGCTAGTCGAGATATCAATTTTCACCCCTTCCATATATTTACCGTACTAACTAGTATAACTATTATTGTCACAATTTGAACCGTATGCCATTTATTTTTACAGCAAAATTAGGTTGGTTAATGACTTGCTGGAGAGCATTCATTTTGTTGCTTCTACTGAAGCAATGTTTATTGGTGTTCGAGCTGGGATTCATCCATCAATTATCTACGACATAATATCAAATGCTGCAGGAAGCTCAAGGTTACAAGGATTATTGAATTCTTTGTGTTCTGTCTGAACTCGTGATACTTGTAGGTTTATATTTCAACTTATGATCATACAATTTATTGACAGGATTTTTGTGGAGGTGGTTCCAAAATTTTTGAGTGAAGATCCATTGCTTATTGATTTTCTGAAGTCACTAAAGAAACATGCAGTAAGTTCCTTTGCTTCTCCTTTGGGGCATGGTATGGGATTCTTAGGATACTTTACGCAATATATGAACAGTGCAGATAATTAAGTATGTCCTTTTTCTCCCTTTTATGAAAGAGGGGATTGCACAAACTAGAATGTTATTTTAAGTAAAATATATAAATATGTAGGTGACGTGCACATTTATTGCTACGGAGTTTGTATGGACAATGAATGAAGGCATCCTATGCGTATTGGAGCCATGACCGGTGGTAAGAGCAGGGTAGCTGGGATAACAACAACAACAACAACAACAACAACAAAGCCTTTAGTCCCAAACAAGTTGGGGTAGGCTAGAGGTGAAACCCATAAGATCTCGCAACCAACTCATGGCTCTGGCACATGGATAGCAAGCTTCCACGCATGACATTCCGCGGTATCTTATCATAGGCCTTCTCCAAGTCAATGAACACCATATGCAAGTCCTTCTTTTGCTCCCTATATCTCTCCATAAGTTGTCGTACCAAGAAAATGGCTTCCATGGTCGACCTCCCAGGCATGAAACCAAACTGATTTTTGGTCATGCTTGTCATTCTTCTTAAGCGGTGCTCAATGACTCTCCCATAGCTTCATTGTATGGCTCATCAGCTTAATTCCACGGTAATTAGTACAACTTTGAACATCCCCCTTGTTCTTGAAGATTGGTACTAATATACTCCGTCTCCATTCTTCTGCCATCTTGTTTGCCCGAAAAATGAGGTTGAAAAGCTTGGTTAGCCATACTATCGCTATGTCCCCGAGACCTTTCTACACCTCAATGGGGATACAATCAGGGCCCATCGCCTTGCCTCCTTTCATCCTTTTTAAAGCCTCCTTGACCTCAGACTCCTGGATTCGCCGCACAAAACGCATGCTGGTCTCATCAAAGGAGTCGTCCAGTTCGATGGTAGAACTCTCATTCTCCCCATTGAATAGTTTGTCGAAGTACTCCCGCCATCTATGCTTAATCTCCTCGTCCTTCACCAAGAGTTGGCCTGCTCCGTCCTTGATGCATTTGACTTGGCCAATATCCCTCGTCTTCCTCTCTCGGATCTTGGCCATCTTATAGATGTCCCTTTCGCAGGATGTGATATTGAAAATGGAGGGTCCGGCACTAGCAATTGGAAGAGAGGAGATTTCTTGTTTGATCAAGGTATTCAATCCTCTTCCCAAAAATAGGGTAGGTACTGGACAGGAGATTAGATATACATGAGAATGTTGCCGCGGCAAAGGTGGCAGGGTGCTGAAAAACCAGGGGAGGGGAACTGGCAGGGACGAAGCTAGGAAAAAAGTTTGTTGGGGTCATGTCTATGGCAGTGGGGTCATCTTCAATAAATAGATATTGTTTAGTACTAAATTAATGAAGGATTTCCTAATTTCATTGGGGTCAATTGACCCTATGCCTACAAGGCAGCTTCGTCCCTGGGAAATGGTGAGAAGTGATAAATAGTAGGGGTATCACATTGTACCTTGATAGATAGCAATCAAGTTACTTAAGAAAGCTAGCTAATCCCGTGGACTTCATTTAGCAGCATATTTGAGATATTTCAGTGATTTTTTTGCACACTTTCTTTTAACTATAGTTTGTATTTAAGTTTTGTGTCCAGTTCCATGCTCTTAATCTTGTAAGTCAACCCCATTTTTGAGAGAGGATTAAATGGTTAATTAGTAATGTGCCACTCTGATCTTACAAACTGTAAAGGAATATTGCTACTGCATGGCTGATTGCCTAACTGCTTCTGTTTATTGAAACAAAGTTTTCGCTCTTAAACAATATAGAGCTACGTGATGGATACGGCTAAAGCAGCAACGTTCCCTCTGCCACTTCTAGCTGTTGCCTATCAACAATTAATACATGGTATGATGTTAAAGCTTCTTTACTTCCAATACATCTTGAAAGTCTCCTGATAGCCCGAACATGCTAGTACCTTCTTATTCATGCACATTTTTGTACTTATCTTCAGGTTCTTCAGCAGTAATTGGCAATGAATCCGCTTCGCCGTTGAAGGTGTGGAATAACTGTCAGCTTTCTCAAGATGCTTCAATTTATCAAAATTATCTTAATTTAAATCATCATTTTGCAGGTTTGGGAACAACTATTTGGAGTAAACATCGTAGATGCTGCCAGTCAACAAATCTATGACGCAAGCAAATTAGCTGACCAGCTTGTTATGGCCTCGAAAGCAGCAAAGAGGATCGGCTTTATTGGTCTTGGAGCTATGGGCTTTGGAATGGCATCTCATTTGCTAAAATCAGGGTTCTCCGTAACTGCTTATGATGTAGGAACTCTTCTGCTCTTGTTCTTCCCAACTGCATAATTGACCGTTTGATTGCTTCAATTCAAAATACAAAGAATGAAAGGGTCTTCACTTGGTTTCTTCTGCATTAGTGCTTCTGTACATATGTTGCATTTCAAGTACCCTGAACAATCTTATGGATCATAGATTTGACACTCTGTGGTTTTAAGCCTGTAATTTTCCTAGATGGAACAGTGGATGGCTTAGATTAATCTTAAGGTATCAGAAAGGGCAGCAGTAATTCGAATAATAAACGAATAAATCACAAACTCTTCTCTTTTCAGTAGTCCATTGGGCGCTCTGAATTTACACAAATATACAGTTGAGGAAATAGGATACTGTTCACCCTTTTCAACTTACATATCTTTTGTTTGTAAACGATGTTATAGACCTATAGTGTTATGCATGTTCTTTTGTAAAAAAAAAGTTCTTCCAATCCACACGTGAATAATTTTATACCCTTTACCCATTAGGTCTACAAGCCAACACTGGCCAGGTTTGCTGCTTTAGGTGGATTGACCAAAGATTCTCCTGAGGAAGTATCAAGAGGTACGTTGCAACGTTCTAGTATAAAGTATAATTCTCGCTTCTTAAGTTTACATTTGTTACCTATAAAGTATAATCAGTACTATATGATTTTCTGACTGAATGAAATTAGTTCAGACCTCATTATTCTCTTGTATTTATCTTATTCTTTATTTTTGTGCCATTCATCGAGTCAAAAGGTAAAGGTAAATGACATGTGGGCCCAGCCCCACCTCATCACTACTTAACAGCAATAGCATGGAATGGAAACACCGTTTATGATGTATCAACCGTGATACCCTGTCTGAAGTATTAACCATAAATTGGTTTCAGATGTGGAAATCCTTATCATCATGGTTGCAAATGAAGTTCAAGCTGAAAGTGTCCTATATGGAAATGCTGGTGCTGTCTCAGGTAACATATAGAGCCCCCCCCCCCCCCCCCCTGCTGGTTCTGCTGATTATTAGCATCTCCCAGGTAGGTTAACTATTTTTTGAGCTGTACTTACATGTTTGTGAATATCCTTTGTCTTTCACTTTCAGTTTTGCCAGCGGGAACATCCATCATTCTGTCATCTACAGTTTCTCCTGGCTTTGTGACTCAACTCAAAGGAAGATTGGAAGGTAGCCGCTCTTTTTTATCTCTCCTGGGCATGTTTGCATTAAAAATAAGAAAGAATTACCACAAACATTACTGGTCAACAAAAGCCACATTGTGTAACTCATAAATCCAACTCATGAAAATCTAAAATCTTCTGTTTGTGATTCCGGGTGAGAGTTGAGTCCTGTAGCTTCAGTGTCTCACAAGTAATGTTCGTTTACCCTTTCTCATTATAAAACACCAGATTTATAATCATTTGCAGAAGAAGTTGCAATGACTGCTGTGACAGCTAGCCTGTATTTTGTGTTGGGATGTTGTTATGAAGTTGAAGTTGCATTTTAAGGGAAATGTTCCGGCAGAAGGCCTTTGGGGTGTTACCCAGGTTCCAAAAGTCACAGAAACATCCAAAAAGTAGTACAACGCTAACAGAAGATGAATCTATTACTAAACAAATCAGATCCATATTTGACCTATGATCAGAAAGCTGTGATAGAAGCTTCAATCATTTTCAGCTATTGTCGAAAGGAATCTGAATTTTGTTTTCTCCATGTGTAGGTTAAATCCGTATCTCGTTGATTTATGTCATGTAAATTGTGTCAAGAGCACTTATTTTTTTGTCACTTTTTATATTGTTTCCCGTAATACACCAAGGCCTTGTAGTACTAGAACCATTCTCTACATTTTAAATATGTCGGCAATGTTGTTTACTGCTTTCACTGATGAATCTTTTGGAATCTTTAACTACTTCTATACAGCTGAATGCCGAGAGATAAAGTTGGTTGATGCTCCGGTATCTGGTGGTGTCAAGAGAGCAGCGGACGGGACACTAACTGTATGATTAGCGAAACATTAATCTAGTAGAAGTGACGATGTAACCTTTATGGATATAAATATTTGCACTTCTTTTGACAGGTAATTGTTTCGGGAACAGATGAAGCTCTGCATTGTACGGGTAGAGTTTTGTCAGGTATGAAAAAGGTTATCTGAACACCATCATATGCTGTATGCAAAGTACGGAAACTGGTCGATAACTATTATCTTATTTTTGCGTAACAGCACTAAGTGAGAAACTATATCTCATCAAAGGAGGATGCGGAGCTGCGAGGTGATACACATATTTTGTAGTCATATACCTTCCTTACAGGGTCTTATTTCTGCATAATGGCTCCTTTTCATTAGTGCTGGCGCTTGTGAATCAACATCTCTCTTCTGCTACTTTTCCCAGTTCTGTTAAAATGGTGAACCAACTTCTTGCTGGGGTCCATATAGCCTCAGCAGCTGAAGCAATGGCATTTGGTGCTCGATTAAATTTGCGAACAAGAAGAGTTTTCGAAATTATTCAACATGCAAGAGGTTACTCGTGGTATGTGTCATTGTTTAATCCACTCTAATGAATCTTCCTTTTTTACTCTTCACTGTTTAGACTTGCTAATATTGGCCGAGTGATAAAATGATTATGAGAAAGAAACAAAACAATTATTTCTACTTGTTTTTGCTATCAGCTCCCTGTAGGTTAGTCTTTCAGATATCTAGTGGTGTGTTCTAGCATGAGTGAAGGTGACTATCTGATGATTGATACACTCAATCTTCATATTGCAGGATGTTTGGAAACCGTGTGCCACACATGCTTGATAATGATTATACACCTTTATCTGCGGTGGACATATTCGTCAAGGATCTTGTATGGATAACCGAGCTATTAACTTATTCATTTTATGAACTATTATCTGTGTTCGTAAATGTAAGACGTTTCTGCAGTTCAATTTGAACTGCAAAAACGTCTTACATTTAGGAACAGAGGGAGTACTTGTTATGGCGCTGCTAGAAGGAGGGTGCGAGAGGGCCGGCCGGGGGCCTTTTGCCCACGGCCGGGCAAGATGGAAGGGATTTCCTTCTTAATTCTTGCTTCATTAGATTGATACACCTCCTCTTTATATATAGAGAGGTTTACTTGACTCCCAAGCAAGCGACCCTTATCTCTAATTAACCCTAAGGCTAACGGGCTATAACGCCAGCCTAGGCCATTAGGCCCATTACGTACTCTAACACTACACCCCACCTAGACATGCAGCTTGTCCTTGAGCTGCAGCCTAACCAACTTATAACCATGATTCGACGCAACACAAACCTAACACCTAAAAACAAGCCTTTTACATCTCGGCTTGTTTTATTACTCTCAACCTGAAATGGACTGGGACGATTTATTTTGGACCCCTTAACAAAAAGTGGACACCATCCGCATGTCGGACGTGCACGTGTACAGCCACCTGGATCCCATGTACACCATCTGGACAAAAGGAGTGCATGTGTATGGCCACCTGGAAGTGGTCGCAAGAGCAACCAGCAGAGGCGCCCTCGCGGCGGCCGGCGGAATGCAGTGGTGCTACGCGGTGCGCTCCTGTCGGTGACACCCTGCCTTCTCCCTGCCGGACGGCTGTTGGTCTGCACCGCACAAGGAAGAGTGGAGGGCTTGCGATGGAGAATGACGAGGAGGGGTGCGCCCCCCCAACCGCCGATGTCGCAGACTTTGAGGTCGCTGCCCGCAGGGAAAACGGCATGCCCAAGATCCCCGACGCAGCGGACAAGATCGAGGTCCTTTGCGCAGCGAAGCGCAGCTGGGAGGAGCGGCAGCTGCAAACCACGCATCTCCCGCACACGCCGACGCGCTAGGAGGCCGCGCGCAGCAGCCTGTAGCCTCACCACCGCCGACACGTGGCGGGTAGTGATCCAAGCCGGAAACGGTGACGGCGACGGCGGGAACTTGATTTGCTGGATGGGGACGCCCTGGGGAAGCGGTAATGGCGACGGCGGGAACTTGATCTGGTGGATCGGGACTCCCACCGGCGCGGTCGATGCGGGGCCGGAGCTGACCGGCGGTGGCTGCTGCAGCGGAGCGCCGGGCGCGGCGGAAGCCGCCAGGAGCGGCGGCTGCCACTGTAGCCAGGGCGGGGCGGTGGTGGCAATGGATGGCAGCTGCAGCGGCTGCTGCACTGCGAGCGCGGCGGGGACGCCCTGGGGCAGCGGCAGCGGCTGCTGCTGCGGAGCGCCGGGCGCGGCGGAGGCCGCACTGCAGCCAGGGCGGGGCAGTGGATGGCAGCTGCAGCGGCTGCTGCAACAGCCAGGCGAGCGCGGCGGAGGCCGCCTGGTGCAGCGGCTGCCACGGCAGCCACGGCGGCACGGTGGCGGCGATGGGCGGTGCAGCCGGGTGCGGCCCGTAGGACCCGGCCAAGAACAGGCGGATCTCCTGGACCGCCTGGGTTAGGTCCAGCAGCGCCCCGGACACCTCCTCCGGGGTGAGGACGGCGGAGGCGGGCGCGACGGAGGATCCCGGCGCGGGCAGGAGCGGGGCGACGGTCGTGGTGGCCGCCGGAGGCGACGAGGTGACCGGCAGCGGAAGAGACGAGTTGGGCGGCGGTGAAGACATGATCGAACCGAAGCTAGCTGATACCAAATTGGTATCAGAGCTGCTATAAGGAGGGCACGAGAGGGCCGGCCGGGGGCCTTTTGCCCACGGCCGGGCAAGATGGAAGGGATTTCCTTCTTAATTCTTGCTTCATTAGATTGATACACCTCCTCTTTTTATATAGAGAGGTTTACTTGACTCCCAAGCAAGCGACCCTTATCTCTAATTAACCCTAAGACTAACGGGCTATAACGCCAGCCCAGGCCATTAGGCCCATTACGTACTCTAACACTAACAAAAGATAATAATGCATTTATCTCGTCTTGAACTGCAAAAACCTCTTACATTTGGGAACAGAGGGAGTACTAGCAAAAGGTAATAATGCACTTATCTCGTCTATTTCAGGGTATAGTATCTCGTGAAAGCTCCAACTTGAGGATCCCATTGCATGTCTCTAGTGTTGCTCATCAGCTGTTTGTTTCAGGTAATTGTCTGTGATAAATCAAAGATCATTTTCCATTCTATGTTTTGGTTCCAATGTTCATCAAGTCGTGCTATATTTCTTATCTTCAATGGAAGTAGTTACCCAAATTAACAGAACGTTGCATGGCCGTCCCCTCTGTTTTCTATACTAGTAGTATATTGCAGTTAGTTTTAGAAGAAAACAAATCAGCTTACAGTTATTGTTTGGATAACAGTCTTAATTTTTAAATAGGATGGCTATTGTTTTATTCATTTGATGTATTCCAACAGGCTCTGCTTCTGGATGGGGTCGATATGATGATAGTGCTGTTGTAAAGGTGAGCAGCTCACAGCTCTCAGTAGACAGAACTTATGCCAAAATATGATATACTGATAGCCTTAATTTAATTTCTTTTCCTAAACGTGCCCGCTGATTGCTCCTGGCACAGGTATATGAGACATTATCTGGAGTAAAAGTAGAAGGAAGACCACCCATGCTTAATAAAGAAGATGTCCTGCGTTCTCTTCCTGTTGAGTGGCCAGAAGTTCCAATGGATGATCTTGTTTCCTCTGCATCTCAAGATAGCAAAAAGGTTCTTGTAGTTTTAGATGATGATCCAACTGGAACTCAAACAGTTCATGATATAGAGGTCTTAACTGAATGGTAAACGAAAGCATATTGTCTTTTAGATTACATATGTTTTCTGCTAGCATTTTCGTTTTGCCCATGTTCCGTACATAGTTCCATAAGCTTTGCTTGTAATTCTGAACTGCAGGCCAGTTGAAGCCCTCACAGAGCAGTTTCTGAAACTACCAACCTGCTTTTTTATTTTGACAAACTCCCGTTCAATGATTGCTGATAAGGTAGGAAAGTGCGGTCTTAAACAGTTTATCTTTAGGGTATTTAAATATATATTTAAAGCGTTCTCACTTTCATAGGCCGCACTGTTAGTAAAAGATATCTGCAGAAATCTTGAAGCTGCAGCAAAGACTGTCCCTGGTATTAGTTATACAGTGGTCTTGCGAGGTGATTCCACTCTACGTGGCCATTTCCCAGAGGTTAATATCTGAAATTCTCTCTGTTTTTCTTTTCCATTTCATTTCTCTGATCATATCATACTCTTCTACTTTCCAGGAAGCTGATGCAGTTGTTTCAGTATTAGGCGACATGGATGCTTGGATAATATGCCCATTCTTCCTTCAAGGTGGGCGGTACACTATTGATGATATTCATTATGTTGCAGACTCTGAAAGGTAGTCTGTGCACGATCACTATGCAATGTATTTAAATACAAAAATAACACATAACAATATTGATGTCCAACATTCTCTAACAGATTGATTCCTGCTGGTGAGACTGAGTTTGCCAAGGATGCAGCTTTTGGCTATACATCTTCCAATCTCAAACAGGTACTTGGAATTGTTTGATTGGTAAAGCGAAGAGCGAAGAACAAGACCTTAGTAACTTGCTTTGTCCTTGAATTTACAGTGGGTTGAAGAAAAGACTAAAGGAAGGATTTTGGAGAACCAAGTTTCAACAATTTCCATAAGTCTTTTACGTAAAGAAGGACCAGATGCAGTCTGTCAGCTTCTCTGCAGCTTGGAGAAGGTATGTGATAGTAAGCATGGAAAATATGATTGGCGGAGCTTGGAACAAAATGAAGGAGAGGCCAACTATGATTGTTACATGAATTAAGATGTATTTTTGTTGTTTAATTAGCAAAGAAGCTTCACCTATTTAAACAAAGTAGTTACAAGATTAAGGGCAGGCCAAGGCGCACCTTAGTCACCACTACGCCCCTCCAGTGCATATGAACAGTTGTTTTGAGTCAGTTTAAACTTTTTAAGGATATAATTATAAATTATTAGTTTTGTAGATATTTCATATTCTCGTTTCTGTATGCACTCATGGCAGCTATTTACTTGAAATGGCTAATAAATGTTGCAGGTGTTTTAGGGACTCTAGAACTGTACTGGCTGATTCAACCATATAGTATCATGAATCCTGTTATGATTCCCTCTCTACTTGATTTACCTTGAGAATTTCCTGACAGGCATTTTGACTCTTTCTAGGGCTCTGCGTGTATTGTCAACGCTGCCAGTGAAAGGGACATGAATGTCTTTGCTGCTGGAATGATCCAGGTATGTTATGCCACTCCATTTCTACGAGAAGCATTTGTTCTTAGTGTTAAGCTTCATGCACTAGCCAACGCAACCAAAAGTCCAAACTGATGGTAAGGGCTAGCAATCCACATATACACTTCAACACCCCTTATCACGTGTGATGCGGAAAGTCAACACATGGATAGACTAAGAGATATGGCTCAAGAGGCCTATATGTGGACACAAAGGGGGGAACAACAATTTTAGATTAATTGTGAAAGCCAGGACTTAAACTCAAGACCTTAGGCTCTGATACCATGTTAAGCTTCATGCACTAGTCAACGCAACCAAAAGTCCGAACTGATGGAAAGGGCTAGGCAATCCACATATACACTTAAACACTCGGTACCTATTCCTTTCGAGTTGATATGATAATATGTCTTATTCTAGTATTGATGCTCTGTTTTGATCTCCTATTCTTCTTTTTCTTTTTCCGTTAACTCAACAGGCTGAATTGCAAGGAAAGCGGTTTCTGTGTCGCACAGCTGCAAGTTTTGTGAGTGCTAGAATTGGAATCAAGCCAAAGCCACCTATCCGCCCAAATGACCTTGGATTAAAAAGAAATTTAGCGGGAGGCCTCATAGTTGTTGGTTCTTATGTGCCAAAAACTACGAAGCAGGTTAGCCTTGTGTTGCTTTAAGATTTTGTCAATCATATTTATCTCATTTGCATGTTCTTTTGCATTTGTTAGGTCGACGAGCTTCGTTCACAATGCGCACAATCCCTCAGAGTGATAGAGGTAATTTATATCTTGGCTGTCATGCCTAAACATGAACGATCAACCATCGTATACATCTACTTGGATAGAGTTTGTGGATAGTACTGTCTTAAGTCGGATGGTTTTGCTTGAGATCAATTTGTGGTGTTAGTATTGTGTTCTCTTTTTACTCACTGTAGGTATCTGTTGAGATGATCTCACTGAAATCAACTGAGGAGAGAGACCAAGAAATCAGCAGAATTGTTGAATTGGGAAATGCTTATATACAGTCTGGGAGAGACACGCTAATCGTCACCAGCCGCCAACTCATTACCGGAAAAAGTTAGCATCTTTCTGACTATCATATGGCTAAATGTGTTGTTTGTTCCATTAGCAAATACAGTGATCAAATGCTGAGTCAATTTTGATTTCTAGTTTCTTAATGCCAACAATTCCTCAGCCACATCCTACCTGCCGAAGTGTTGAGCAAGTTGTGCTGGTAAAAACTGTTTACTAACAAATGATGTGGAGATCCTCAGGGCTAGCACACAATTGCCTCCAAACTCCACACCGTATCAGAGGCTCTCAGCCTAATTCTATTCCCTGAGCTCAACTCAGATCACTTCTGGGGAGATAGTTTGTTTTCTTTTATCATATTCTAGGCATGAACCGGGAGTCTGGGGCTAACTGTATTGGAAGACAGGTCAAACTGTCAAAACTTTTTAAAAGGGGGGAATTGATAAAATAAAGTTGCTTATTGTTGTCAAAATCATGCCCAAAAACGCATTAGCCACCAGTAGAGCCATTGAATGAATCTCTACATTTAACCACAACCACAATCACAACGTGAGTGCATTGTCCCAATCGTTTGAATAGAACATCTCCAGTTTGCATCATAATCTTTTCTAGTGACCAAAACCAAACTAACTTCAGTATACGTTCCTGAAATTGTGTGCTAGAGTCAGATACCTAACTCAGCTTTTCAAAAGAGTATGTTCAAGAATAGCCGCTGTTATCTTCATGTAAGCATTAGCAGTACAAGACAACTGACCTATTAGTATAGTTTGCTACCTTTTGTGTGCCAGACGAAAGAAGATAATTTCTTTCTTGGATATCTGTACTGAAGTAGTTTGACCACCTAACAAGATTGTACCTTTCATGTTGCAGCTCCTGAAGAAAGCTTAGAAATTAATTACAAAGTGAGCTCAGCACTAGTAGAGATTGTGCGAAGAATTGATAGTCGACCCCGTTACATCCTTGCAAAGGTAAAATTACACAGCCGATTTCTTCCTTCTGAACAAATTGAAATTTAGTTTTTACTTGGTTCCTGGAAATTTGCCAATCTCTGGATCTGTTACTGTTAGGTCAAAATATTTGTTCTGTTTTATTGGACCTATGCCATTAAATACTTAATGAAATATCGAGATAGCATTACACCTACATGGTTACATGTTTCATTCATCAGAACAAAAAAGAAGAAGAAAGAATAGCATCTACAAAAAAAACAGTGCCTGCAAATTCAACTGTCCATACCAAACAAAATGATTGATTCCACAAGTTGTGCATGGAATACTTCTGGAGGTACTCCTTACTATATTCTTTATACAGGGAGGAATCACTTCATCTGATCTTGCTACAAAAGCTTTGGAAGCTCGGCGTGCCAAAGTAATGGGACAAGCTTTAGCTGGTGTGCCTTTGTGGCAGCTTGGCCCTGAGAGTAGACATCCTGGTGTCCCCTACATTGTTTTTCCTGGTAACAAACATATCTCATTTCTGTACCAACAGATAAATAACGTTACCTTTTTACTCAAGAAACTATCTTGCCACTCCCCAAATGACAGGTAATGTTGGTGATAACAGTGCTCTTGCAGAAGTGGTCCAGAATTGGGCTTGCCCTTCTAGAAGTTCAACAAAAGAACTTCTCCTTGTAAGTTCTTATCCACCGTCTACTTCTCATCAAGGTTATACATCGTTGTGTATTGCTTTAAATACTTAATCAGCTATTTTTTTCGTATTGTGAAGGACGCGGAGAAGAGTGGCTATGCAGTTGGTGCTTTCAATGTATATAATCTTGAGGGAATTGAAGCTGTAATTGCAGCTGCCGAGGCTGAAGAAAGTCCTGCTATCCTGCAGGTTAGGAATTCTTTGTTAGTATAGGATGTTTTCTTTGGCAGTTTATGGTGAAAATTACTCCCTCCGTCCCATAATATAAGAGCGTTTTTTACACTACACTAGTGTCAAAAACGCTCTTATATTATGGGACAGAGGGAGTATGTAATTTACCATGTTGGGTGTCCTCTGATACCATTCTTTACTGGAGTATGTTATGATAGGACTAGTAAGTAGGTCGCAACAACTAGCTCCAGAGGGATAGAAGCAATACCACACAGATCCTGAAATGGATTTGTACCCCGTGTTTTTTCCGTAGAGAGAGTTGCATTGGTTCTCTCAGAGTACTCGTTCTCTCAACTCATGAGCCTTTCTCACTCTCAACACATGAGCTTTTCTCACTCTCAACTCATGAGCTTTTCTGCACCCATGCTCCCTGAACTCATTATCCATATTTTTTTGTAGATGATATGTATCGTCTTGATTTCTAACCACGCTTATTGTGGTTGTTATTTAGGTTCATCCCAGTTCCCTGAAGCAAGGTGGAGTTCCGTTGGTAGCATGCTGCATTGCTGCAGCAGAACGAGCCAGCGTAAGACAAACAGAACTTAGCATGCTTAAACAGTAAATGTGGACACCCAGTGATCTGGATAAAAATAGTTCATAAATTTTTATATAACTTGGGTTCCCACATATTACCTACTAGCCATCCTTGTTTTTTGAAACAGATAGCCATCCTTGTTTTATGTTCTTTAAAGGTGATCATTCTGTGATTGTCACTCAGGTACCTATCACTGTCCATTATGATCACGGGGCTGACAAGCATGATTTGCTTGGAGCTCTTGAAATGGTAAGTGAAAAAATGCGTTGTTTTCTCAAATTTGCGGTACCAAAATGTTTTCCTAATCATGAATGATAAATGACTACATCTGAATCTTAATTCTCTGGACCATCTACTATGACCTTTTATTTTTCAGGGATTTGATTCAGTCATGGTGGATGGTTCCCATCTAACTTTAGAAAAGAACATCTTATACACAAAGAACATATCTTCCCTGGCTCATGCTAAAGGCATGCTTGTGGAAGCTGAACTTGGGAGACTATCAGGCACTGAAGATGGCCTGACAGTTGAAGAATATGAAGCGAGATTTACTGATATTGCTCAGGTTATTTTATCTTCCCAACAAATTTCAACTCTATGGCATTCTATCATTACAATCCTGTAATAATGCTTAACTTATATGTTACATATTTTCAGGCTGAGCAGTTTATTGATGAAACTGGTATTGATGCATTAGCAGTATGCATTGGGAATGTTCACGGAAAATATCCTCCCAGTGGACCGAACCTGAGACTTGACTTGCTAAAGGTTTGACAGAACAGCTATGAAAATCTTTGCTTGATCTTTCAGATTATGTGTGTGTTACCAGATCTTTTACCCTGTTTTCTGATTTTCCGTTTTTTCTAAAATTGTTAAGATTTTTCCAAAAAAGTATGATCCTACTTTGATTTTGTGTGTGTTTTACTTAATCTGGTAACATGACAAGGTGCTGCGTATATGTGATGCTACTTGTCTTCCATGTGTTGTTTGAGTCAGCTTGAACCGCTTGGAATAGAACCAAGGGGGTAAACCAGTAAACATATTCAACAGTTCAAGGTTTAAGACAACTGGTTTTCTGGTTTTTGAGTTTAAGATTGAAAAATGAACTTCTACAATGGTTCAGGGTTGTAAAGTGTTTTTTTTTCCGACGGCTTGTGAAGTATTCTGTAAAGAATTACCAAGCAAATTTGTGCTTTTTGCAAGCAACTTCTTTTGAGTAATGGACTCGGTATTTTTTTTCATTGAAACGATGACAGGAACTTCGCGCATTAACTATGAAGAAAGGAGTTAGCTTGGTACTTCATGGAGCATCTGGCCTCCCTCATGAACTTGTAAAGGTTTGATAAACTGTTTCCTTCTGTATTAAGATCAGTGAATCTGTACCTTGCAGTAGTTCTTGTTGAGTTTCCGGCATCAACTTATATCCTGAAAGGTTCAAGTTATAGGAGAATTGGGGGGCAATCGTTGCCCTCCATAAGAAAATCATCAATCATGGTTGCAGTGCATAAAATAATTCAACGACAAGGTTAAAAATGCTGGAGGGCTAGAGTTTCTGTGTCTATATCAGCTTCATAGTTTTGCGAACAATCAGTATAACGCTTCCTTTTGTGAAATAATTATACTAGAGCTTATCAGTTGATGAAATTGGTCTTATTATTAACCAATGTCATATTTGAAATGCTGTTGCGCAAGTGATAATAACCTGTGACCAACTTGTGGAGCAGGAGTGCATAGATTTGGGGGTGAGGAAATTCAACGTGAACACGGAGGTCCGCAACAGCTACCTGCAGTCACTCAAGAAGCCAGAAAAGGACCTGGTTCAGGTCATGGAGTCTGCCAAGGAAGCAATGAAAGCTGTTGTAGCAGAGAAGATGCGCCTCTTCGGATCCGCAGGCAAAGCCTGAATGTAGCGATACAATGAAAACTGGAGCCAGCAACACTCCATCATTAGGTGTTGCATGCCCATTTTAGCGATCCTGCTAGGCTTGTGACAGCGAATTGCCGGAACCAGTTTCTTAATAAAGCACTGATACAGTTGCCTGAAGAAATAAAGTAGCAAAAATTAGTAAATCGCAGCAACTTACCGGTGCTCCCGTTCATGGTTGAGTGATTAGTTTTGCCTTTAATATGATGTTTAATAATGAGTTGTTATTTAGTCGTAATTTGTGACATGTGAAATAAATTCCAAAATGATTTCG

>TaFBA20

TGCCATTGCTTCCAGCAAAATAAAAACGTGGTTCCAGCAAAACACACAAAATGGCATGGCCCAAGAACAATGAAAAAGATTCCAGCAAAACAAAATACGATGCTTCCAACAGAAACGAAAATGCTTCCAGCAAAAACACTTGCCGTTTCCAGCAAAATCATGAACTGTCGTCATCGGGTCGTAGCAAAATAAATCGCTGCTTCCAACAAAAAAAAAGTGCTGGTTCCAGCAACGGCGGATCGTCGTCGCGGCCGTCGCAACACGGCTATCGCCGGTTCCAGCTTCCGCTGTGCCGTCCTGCACGCGTGCTCCGCCATATGCAACAAAACTGACAGCCGGTTCCAGCAAATCGGCCTGCTGGTTCCAGCTTTCGTCTTATCCGCCTTTGTAGTAGGCTGCAGCAATGCCGGCGTGCTCCCGCGTATGCGACAAATCGGGCACCAGTTCCAGCAAATCGGCTTACTGGTTCCAGCATCGCGCGACCACCGTGGCAGCACGCCTCCTTGTCGGTTCCAGCATCTCCCGACAACGGTGGCTGCACGCCTCCTTGCCGGTTCCAGCATCGCCCGACCACGGTGGCTGCACGCCTCCTTGCCGGTTCCAGCTCGTGGACGCAGCAACTCCTCAGCCAACACTCTGCATCTCCGCATCTGGCGCATAAAAATCTCTCCCCCATCTCCGCCATTGCAGCTCCTGCCCTCCCGTCGGAGCTACGGATCCAACCTTGAATCAACACCGATTGCCGTGGCTCGGGAGCTCGCCGAAGGCTACCGGAAGGCAGAGGCGATAAGAATCTACCGGAGGCGACAAGAAGCTACTGGAGGACGAGAGAGGAAGGGAACGTCTAGCTCGTGTGGTGGGGAAGAACAGGGTAGAAATGTGTGGATGAGAGAGGATAAGGAGGTGGAAGACGTTGGACACATGGGCCCATGTTGGATGCGTGCACGCACGATCGAGAGGGATCGAGAGGCCCGCGTGCGTCCGGCGCGACGTTTCGGCCGGCGCACTGGGGGGAAACGTTTTCCTTTAACTTTTGTGCAAGGGAAATCCTTACACTTGGATGCACTGTTTATGAACTGTTCTATATCGAGATCAATCGACGACCTACTAAGCAAGTTGATCTTTATATATGGAAGTATAAAAAAATGCTTATCTTTAACAAAGCCAAAAGCAAGATCCTTACATTGCGCTGTGTCCCATTTGTCACGTGCACTTCATTTTTTTTTAAATCTCATGGGACATACACTCCTGTGCAAAGTTCAGTTCCACGATTGGCACGCTGTAGTACAGAATCGCATCATCATCTTTCCGCTCACAGGGCCCACACGCTCAGAACCCGTGAGCATCCTGTCTTTTCGCGCTAACAAAATGGCGTCCTCGTCCGAAATCCAAACGTGGTTCCGTCCTAGCCCACATGCGCCACGCAGGATTAGCACCGCGCATCGGGCCCGCGATCCAGTGACACGGAGCGGTTGGGTAAAAGTCCAGAGCAGTGGAGTCCTCGTGTGTCACTCTTCCTGACTTCCTCACTCCCCTGTCTGCTTCCGCTCTGCTCGCCTCTGCTTCGGGCTGGTCGTCGCCGTCGGCGAGCGGGATCACACGCCACTCGCGGGATGTCGCCGTCGTCCTCGGCGGCGAAGGTCGTCGCCTTCGTCGGCGCCGACGGGCTGAGCGCCGCGCTCGCCGCTTCTTTCACCCGCTCTGGCGCGATCGTCCGCTTCTACATCGACCCCAAGGTCGCCTTTGCTCGATCGCCCTTCCCTCGTTTCCCCGTTCCTTTCACCCCCGTTTTGGTTTTTTGCCTCCTCTCGGAGGTGTTTTCGCGGGGATGCTCTGTCCTGACGGTGCGTGTCCGTGCAGGCGGATGGATCCGCGGCGACGGCGCTCGCGGAGCAGGGCGGCGGCGTCCGATGCGCCAGCCCTGCCGAAGCCACGCGAGGTGAGCGTATATAACGGTCTCCTGCACTGGACTGGTTCCAGATATGGCCTTCTCATGGCGCGCGTGAATGGCATGCGTGCGTACGAGCCGTTTGTTGGAATGCCGAAGAGACCTGCTAAAGAATACTTCGTGCCGTGCGTGTGTTGCTTCTATCTGATCTATCAAAAACTCTTGGCGCCTGCAGATTCTGCGCTAGTCGTTGTGCTCAGTGACGCCGATGGTGTAGATGAGTTGTTCTTCGGAGCTCAGGGTATAGTGAAAGGTATGTACTACGTGCTTTCTGTGTTATTGTGTTGCCTGCCAGTCTGTCAATCTCATGCACCTGTTTCTATACAAGTTATATATACACTTGACATTTGATATGATGTTTGCTTGAGCATTTCATAGGATTGTGCAAAGAGGCCATTGTATTAATTCGATCGACGTTGGTGCCTAGTCATCTTGAGAAGTTGGGGCAGAAGCTTGCAGGTATAATGCTAATTTGTGCATATATTGTCTTAGCTGGGAACAGTAGCTGCTGTGCTTGGAATTTTATGTGCACTTTTAAGAATTAGAAAGCTGCACATTTAAAAATTGTAAGGCCCTCTTTGGATGACTGGAATTGAAGAACATAGGAATATGAATATACACATGATTGGAATTTCATGTACTACTATAGTTTCCTACAGGATTTTTTGTAGTGATGACCTTGGATGGTTCACAGGATGTTGGTGATGAATGACTGTACTAGTTAATCTGGATTTTTTTTTTTTTTTTTTTTGCAGATGAGAAGAAGGGTATCTTCCTTCTTGATGCTTACATTTTTAGTGGTTTATCTGATGAACTGAAGCAAAATATTGTCGTAAGTGATACACAGCTGAGAATCTGAACTGTGTGCTTGTATGCTTACGTTATTCTGTTATCACAATTTAACTTGAATGTCAGGTTGTTGCATCTGGGAGAAAGGATGTAGCAGAAAGAGCTGGGCAATTCTTCTCCGGTAGTTCCAAATTTGTGTACCTTACTAATATTTTCTTCCTGGTCCTCATCTTATGATGCTGAAATTTTAAGCTATCTGACATTCCAGGTCTTGACAAAACCATTTACTTAGTCGAAGGTGAATTTGGCTGCAGCAGGTTGCTTTCTTTCCTGAAAATAGCAAATATAAGCTAGTCGAGATATCGATTTTCACCCCTTCCATATATTTACCGTACTAACTAGTGTAACTATTATTGTCACAACTTGAACCGTATGCCATTTATTTTTACAGCAAAATTAGGTTGGTTAACGATTTGCTGGAGAGCATTCATTTTGTTGCTTCTACTGAAGCAATGTTTATTGGTGTTCGAGCTGGGATTCATCCATCAATTATCTACGACATAATATCAAATGCTGCAGGAAGCTCAAGGTTACAAGGATTATTGAATTCTTTGTGTTCTGTCTGAACTCGTGATACTTGTAGGTTTATATTTCAACTTATGATCATACAATTTATTGACAGGATTTTTGTGGAGGTGGTTCCGAAAATTTTGAGTGAAGATCCATTGCTTATTGATTTTCTGAAGTCACTAAAGAAACATGCTGTAAGTTCCTTTGCTTCTCCTTTGGGGTGTGGTATGGGATTCTTAGGATACTTTGCGCAATAAATGAACAGCGCAGATAATTAAGTATATCCTTTGTCTCCCTTTTATGAAAGAGGGGATTGCACAAATTAGAATGCTATTTCAAGTAAAAGATATAAATAAGTAGGTGACGTGCACATTTATTGCTACGAAGTTTGTATCGACAATGAATGAAGGCATCCTATGCGTATTGGAGCCATGACCGGTGGTTAGAGCAGGGTATCTGGGATGTGATATTGAAAAAGGAGGGCCCGGGACTAGCAATTGGAAGAGAGGAGATTTCTTGTTTGATCAAGGTATTCAATCCTCTTCCCGAAAATAGGGTAGGTACTGGACAGGAGATTAGATATACATGAGAATGTTGCCGCGGCAAAGGTGGCAGGGTGATGAAAAACCAGGGGCGCTTGCCGATCTGGGCGCCGGACCAGGAGCCCACTGTCATGGCATGGTGGCTGCAAGCGAACGCGCACACACCGACGACGCATCGCAAGGCCCTTAGGTCCATTGCCTTGCTTGTTCCATGGATGATCTGGAAACACAGGAACACGTGCGTCTTTGACAATGTGACACCCTCGATCGATTTGCTAATAGATAAGATCAAAGACGAGGCCCGCTGCTGGGCTAGCGCGCCGGTGCCCAGGGGCTTAGGGTTGTTCTTCTGCCCACATCCTGGGATGTACACTGATCGGCCTATGCCATTGTAAAATTCACCTCCTAGGAGGCTTGTTCTATCTTTCTTTTCAATGAAATGAAACGCAAAGGTCCTTTGCGTTTTCTCGAAAAATGAAAAACCAGGGGAGGGGAAATGCCGAAATGGTGAGAAGTGATAAATAGTAGGAGTATCGCACTGTACTACTCCTTGATAGATAGCAATCAAGTTACTTAAGGAAGCTAGCTAATCCCGTGGACTTACTCTTTTAGCAGCATACTTGAGATATTTCAGTGAAATTATTGCACACTTTCTTTTAACTATAGTTTGCATTTCAGTTTTGTGTCCAGTTCCATGCTCTTAATCTTGTAAGTCAAATCCATTTTTGAGAGATGATTAAATGGTTAATTAGTAATGTGCCACTCTGATCTTACAAACTGTAAAGAATATTGCTACTGCATGGCTGATTGCCTGAATGCTTCTGTTTATTAAAACAAAGTTTTCGCTCTTAAACAATATAGAGCTACGTGATGGATACAGCTAAAGCAGCAACATTCCCTCTGCCACTTCTAGCTGTTGCCTATCAACAATTAATACATGGTATGATGTTAAAGCTTCTTTACTTCCAACACATCTTGAAATTCTCCTGATAGCCTGAACATGCTAGTACCTTCTTATTCATGCACATTTTTTGTACTTATCTTCAGGTTCTTCAGCAGTAATTGGAGATGAATCCGCTTCACCGTTGAAGGTGTGGAATAACTGTCAGCTTTCTCAAGATGCTTCAATTTATCAAAATTATCTTAATCTAAATCATCATTTTGCAGGTTTGGGAACAACTATTTGGAGTAAACATCATAGATGCTGCCAGTCAACAAATCTATGATGCAAGCGAATTAGCTGACCAGCTTGTTATGGCCTCTAAAGCAGCAAAGAGGATCGGCTTTATTGGTCTTGGAGCTATGGGCTTTGGAATGGCATCTCATTTACTAAAATCAGGGTTCTCCGTAACTGCTTATGATGTAGGAACTCTTCTGCTCTTGTTCTTCCCAACTGCACAATTGACCGTTTGAATTGCTTCAGTTCAAAATATAAAGAATGAAAGGGTCTTCACTTGGTTTCTTCTGCATTAGTGCTTCTGTACATATGTTGCATTTCAAGTACCCTGAACAATTTTATGGATCATAGATTTGACATCTGTGGTTTTAAGCCTATAATTTTGCTAGATGGAATCGTGGATGGCTTAGATTAATCTTAAGGTATCAGAAAGGGCAGCAGTACTTCGAATAATGAACGAATAGATCACAAACTCTTCTCTTTTCAGTAGTCCATCGGGCGCTCTGAATTTACATAAATATACAGTTGAGGAAATAGGATACTGTTCACCCTTTTCAACTTACATATCTTTTGTTTGTAAACGATGTTATAGACCTACAGTGTTATGCATGTTCTTTTGTAAAAAAAAGTTCTTCGGATCCACATGCGAATAATTTAATACCCTTTACCCATTAGGTCTACAAGCCAACACTGGCCAGGTTTGCTGCTTTAGGTGGATTGACTAAAGATTCTCCTGAGGAAGTATCAAGAGGTATGTTACAATCTTCTAGTATAAAGTATAATTCTCGCTTCTTAAGTTTACATAAATTACGTATAACAGTATAAGCAGTACTATATGATTTTCTGACTGAATGAAATTAGTTCAGACCTCATTATTCTCTTGTATTTTTTTTTGTGCCATTCATCGAGTCAAAAGGTAAATGACATGTGGGCCCAGCCCCACATCATCATTACTTAACAGCAATATCATGGTAGTGGAAATACCATTTATGATATATCAACTGTGATACCATGTCTGAAGTATTAACCATAAATTGGTTTCAGATGTGGAAATCCTTATCATCATGGTTGCAAATGAAGTTCAAGCTGAAAGTGTCCTATATGGAAATGCTGGTGCTGTGTCAGGTAACACATATAGAGCCCCCCCCCCCCTTTCCTCATTTTTCCTGGTTCTGCTGATTATTAGCATCTCCCAGGTAGATTAACTATTTTTGAACTGCACTTACTCTTTCTTTTTGACAAACTTGAGCTGTACTTACATGGTTGTGAATATCCTTTGTCTTTCACTTTCAGTTTTGCCAGCGGGAACATCCATCATTCTGTCATCTACAGTTTCTCCTGGCTTTGTGACTCAACTCAAAGGAAGATTGGAAGGTAGCTGCTCTTTTTTATCTCTCCTGGGCATGTTTGCATTAAAAATAGGAAAGAATACCACGAACATTACTGGTCAACAAAAACCACATTGTGTAAATCATAAATCCAACTCATGATAATCTAAAATCTTCTGTTTGTGGTTCCGGGTGAGAGCTGAGTCCTGTAGCTTCAGTGTCTCACAAGTAATGTTTGTTTACCTTTCTCATTATAAAACACCAGATTTATAATCATTTGCAGCAGAAGTTGCAATGACTGCTGTGACAACTAGCCTGTACTTTGTGTTGGGATGTTATTATGAAGTTGAAGTTGCATTTTAAGGGAAATGTTCCGGCAGAAGGCCTTTGGGGTGTTACCAAGGTTCCAAAAGTCACAGAAACATCCAAAAAGTAGTACAACGCTAACAGAAGATAAATCTATTACTAAACAAATCAGATCCATATTTGACCTATGATCAAGAAAGCTGTGATAGAAGCTTCAATCGTTTTCAGCTATTGTCGAAAGGAATCTGATTTTGTTTTCTCCGTGTGTAGGTTAAACCCGTATCTCATTAGATTTATGTCATGTAAATTGCGTCAAGAGCACTTATTTTTTTGTCACTTTTTATATTGTTTCCCGTAACACACCAAGGCCTTGTAGTACTATAACCATTCTCTACATTTTAAATATGTCGGCAATGTTGTTTACTGCTTTCACTGATGAATCTTTTGGAATTATTAACTACTTCTATACAGCCGAATGCCGAGAGATAAAGTTGGTTGATGCTCCGGTATCTGGTGGTGTCAAGAGAGCAGCGGACGGGACACTAACTGTATGATTAGCGAAACATTAATCTAGTAGAAGTGATGATGTAACCTTCATGGATATAAATATTTGCACTTCTTTTGACAGGTAATTGTTTCGGGAACAGATGAAGCTCTGCATTGTACGGGTAGAGTTTTGTCAGGTATGAAAAAGGTTATCTGAACACCATCATATGCTGTATACAAAGTACGGAAACTGGTCGATAACTATTATCTTGTTTTTGCGTAACAGCACTAAGTGAGAAACTATATCTCATCAAGGGAGGATGCGGAGCTGCGAGGTGATACACACATTTTGTAGTCATATACCTTCCTTAAAGGGTCTTATTTCTGCATAATGGCACTTTTTTCACTAGTGCTGGCGCTTGTGAATCAATATCTCTCTTCTGCTATTGTTCCCAGTTCTGTTAAAATGGTGAACCAACTTCTTGCTGGGGTCCATATAGCCTCAGCAGCTGAAGCAATGGCATTTGGTGCTCGATTAAATTTGCGAACAAGAAGGATTTTCGAAATTATTCAACATGCAAGAGGTTACTCGTGGTATGTGTCATTCTTTTATCCACTCTAATGAATCTTCCTTTTTTACTCTTCACTGTTAGACTTGCTTATATTGGCCGAGTAAAATGATTATGAGAAAGAAATGAAACAATTATTTATACTTGTTTTTGCTATCAGCTCCCTGTAGGTCAGTCTTTCAGATATCTAGTGGTGTGTTCTAGCATGAGTGAAGGTGGATATCTGATGATTGATACACTCAATGTTCATATTGCAGGATGTTTGGAAACCGTGTGCCACACATGCTTGATAATGATTATACACCTTTATCTGCGGTGGACATATTTGTCAAGGATCTTGTATGGATAACCGAGCTATTAACTTATTTGTTTTATGAACTATTACCTCTGTTCCTAAATGTAAGACATTTTTGCAGTTCAAATTAGGAAATTGAACTGCAAAAACGTCTTACATTTAGGAACAGAGGGAGTACTAACAAAAGATAATAATGCATTTATCTCGTCTTGAACTGCAAAAACCTCTTACATGTAGGAACAGAGGGAGTACTAACAAAATATAATAATGCATTTATCTCGTCTATTTCAGGGTATAGTATCTCGTGAAAGCTCCAACTTGAGGATCCCATTGCATGTCTCTAGTGTTGCTCATCAGCTGTTTGTTTCAGGTAATTGTCTGTGATAAATCAAAGATCATTTTCCATTCTATGTTTTGGTTCCAATGTTCATCAAGTCATGCTATATTTCTTATCTTCAATGAAAGTAGTTTCCCAAATTAACAGAACATTGCATGGCCATCCCCTCTGTTTTCTATACTAGTATATTACAGTTAGTTTTAGAAGAAAACAAATCAGCTTACAGTTATTGTTTGGATAACACTCTTAAAATAGGATGGCTATTGTTTTATTCATTTGATGTATTTCAACAGGCTCTGCTTCTGGATGGGGTCGATATGATGATAGTGCTGTTGTAAAGGTGAGCAGCTCACAGCTCTCAGTAGACGGAACTTATGCCAAAATATGATATACTAATGGCCTTAATTTAATTCCTTTCCTAAATGTGCCCGCTGATTGCGCCTGGCACAGGTATATGAGACATTAACTGGGGTAAAAGTAGAAGGAAGGCCGCCCATGCTTAATAAAGAAGATGTCCTGCGTTCTCTTCCTGTTGAGTGGCCAGAAGTTCCAATGGATGATCTTGTTTCCTCTGCATCTCATGATAGCAAAAAGGTTCTTGTAGTTTTAGATGATGATCCAACTGGAACTCAAACAGTTCATGATATAGAGGTCTTAACTGAATGGTAAACGAAAGCATATTGTCTTTTAGATTACATATGTTTTCTGCTAGCATTTTCGTTTTGCCCATGTTCCATACATAGTTCCATAAGCTTTGCTTGTAATGCTGAACTGCAGGCCAGTTGAAGCCCTCACAGAGCAGTTTCTGAAACTACCAACCTGCTTTTTTATTTTGACAAACTCCCGTTCAATGACTGCTGATAAGGTAGGAAAGTGCAGTCTTAAACAGTTTATCTTTAGGGTCTTTAAATATATATTTAAAGCCTTCTCACTTTCATAGGCTGCACTGTTAGTAAAAGATATCTGCAGAAATCTTGAAGCTGCAGCAAAGACTGTCCCTGGTATTAGTTATACAGTGGTCTTGCGAGGTGATTCCACTCTACGTGGCCATTTCCCAGAGGTTAATATCCGAAATCCTCTCTGTTTTTCTTTTCCATTTCATTTCTCTGATCATATCATACTCTTCTACTTTCCAGGAAGCTGATGCAGTTGTTTCAGTATTAGGTGACATGGATGCTTGGATAATATGCCCATTCTTCCTTCAAGGTGGGCGGTACACTGTTGATGATATTCATTATGTTGCAGACTCTGAAAGGTAGTCTGTGCACAATCACTATGCAATATATTTGAATACAAAAATAACACATAACAATATTGAGGTCCAACATTCTCTAACAGGTTGATTCCTGCTGGTGAGACTGAGTTTGCCAAGGATGCAGCTTTTGGCTATACATCTTCCAATCTCAAACAGGTACTGTTGTAGAATTGTTTGATTGGTAAAGCGAAGAGCAAGACCTTAGTAACTTGCTTTGTACATGAATGTACAGTGGGTTGAAGAAAAGACTAAAGGAAGGATTTTGGAGAACCAAGTTTCATCAATTTCCATAAGTCTTTTACGTAAAGAAGGACCAGATGCAGTCTGTCAGCTTCTCTGCAGCTTGGAGAAGGTATATGATAATAAACATGGAACATACGATTGGCGGAGCTTGGAACAAAATGAAGGAGAGGCCAACACTAATTGTTAGATGAATTAAGATGTACTCCCTCCATTCCTAAATATAAGTCTTTTTAGAGATTCCAATATGAACTACACACGGAGCAAAATGGGTGAATCTACACTCTAAACTACATCTATATACATCCATATGTAGTCCATATTGAAATCTTTAAAAATACTTGTATTTAGGAACGGAGGGAGTATTTTTGTTGTTTAATTAGCAAAGAAGCTTCACCTATTTAAACAAAGTATACAAGATTAAGGGCAGGCCAAGGCGCACCTTGGTCACCACTACGCCCCTCCAGTGCATATGAAGAGTTATTTTTTAGTCAGTTTAAACTTTTTTGGATATAATTATAAATATTTAGTTTTATTGATATTTTATATTCTCATTTCTGTATGCACTCATGGTAGCTATGTACTTGAAATGTCTAATAAAATGTTGCGGGCGTTTTAGGGACCCTAGAATTGTACTGGCCGATTCAACCATATAGTATCATAAATCCTGTTTTGGTTCCCTCTCTTCTTGATTTAGCTGGAGAATTTCCTGACGGGCATTTTGAATCTTTTTAGGGCTCTGTGTGTATTGTCAATGCTGCTAGTGAGAGGGACATGAATGTCTTTGCTGCTGGAATGATCCAGGCACGTTATGCCGCTCCATTTCTACGAGAAGCATCTGTTCTTAGTTCCTATTCCTTACAAAAGATATGATAATACATCTTATTCAAGTATTGATGCTCTGTTTTGATCTGCTATTCTTCTGTTTCTTTTTACGTAAACTCAACAGGCTGAATTGCAAGGAAAGCGGTTTCTATGTCGCACAGCTGCTAGTTTTGTGAGTGCTAGAATTGGAATCAAGCCAAAGCCACCTATCCGCCCAAATGACCTTGGATTAAAAAGAAATTTAGCGGGAGGCCTCATAGTTGTTGGTTCTTATGTGCCAAAAACTACGAAGCAGGTTAGCCTTGTAATGCTTTAAGATTTTGTCAGTCATATTTATCTCATTCACATGTTCTTTTTGCATTTGTTAGGTCGATGAGCTTCGCTCACAATGCGCACAATCCCTCAGAGTGATAGAGGTAATTTATATCTTGGCTGTCATGTCTAAACATGAACCAGCAACCATCATATACATCTACTTGGATAGAATTTGTGGATAGTACTCTCTTAAGTTGGATGATTTTGCTTTAGATCAATTTGTGGTGTTACTACCGTGTTCTCCTTTTCCTCACTGTAGGTATCTGTTGAGATGATCTCACTGAAATCAACTGAGATAGACCAAGAAATCAGCAGAATTGTTGAATTGGGAAATGCTTATATACAGTCCGGGAGAGACACACTAGTCGTCACCAGCCGCCAACTCATTACTGGAAAAAGTTAGCATCTTTCTGATTATCATATGGCTAAATATGTTGTTTGTTCCATTAGCAAATACAGTGATCAAATGCTGATTCAATTTTGATTTCTAGTTTCTTAATGCCAACAATTCCTCAGCCACATCCTACCTGCCGAATTGTTGAGCAAGTTGTGCTGGTAAGGAACTTTTTACTAACAAACGATGTGGAGATCCTCAGGGCTAGCACACAATTGCCCCCAAACTCCACACCGTATCAGAGACTCTCAGCCTAATTCTAGTCCCTGAGCTCAACTCAGATCACCAGTCAACCACCACCAACATTGTCATCTATCAATCTGCCACGATCCCTACACTGACAGCCATTCCCAGGCATTTGACTTATCTGGGGAGATAGTTTGTTTTCTTTTATCATATTCTAGGCATGAACCGGGAGTCTGGGGCTAACTGTATTGGAAGACAGGTCAAACTGTCGAAACTTGTTAAAAGGGGGGGGATTGATAAAATAAAGTTGCTTATTGTTGTCAAAGTCAAGCCCAAAAATGCATTAGTCACAGGTAGAGCCATGAATCTCTACATTCAACCACAACCACAATCACAATGTGAGCGCATTGTCCCAATCATTTGAATAGAACATCTCCAGTTTGCAGCATAATCTTTTCTAGTGACCAACACCAAACTAACTTCAGTTTACGTTCCTGAAATTGTGTGCTAGAGTCTAGATACCTAACTCAGTTTTTTTTTACGGGAGATACCTAACTCAGCTTTTCAAAACAGTATGTTCAAGAACAGCCGCTGTTATCTTCATGTAAGCATTAGCAGTACAAGACAACTGACTAGTATTGTTTGCTACCTTTTGTGTGCCAAACGAAAGAAGATAATTTCTTTCTTGGATATCTGTCCTGAAGTAGTTTGATCACCTAACAAGATTGTACCTGTCATGTTGCAGCTCCTGAAGAAAGCTTAGAGATTAATTACAAAGTGAGCTCAGCACTAGTAGAGATTGTGCAAAGAATTGATAGTCGACCCCGTTACATCCTTGCAAAGGTAAAATTGCACAGCTCATTTCTTCTTTCTGAAAAAATTGAAATTTAGTTGTTACTTGGTTCCTGGAAATTTGCCAATCTCTGAAGCTGTTACAAGTCAAAATATTTGTTCTCTTTTATTGGACCTATGCCATTAAATTATTAATGAAATATTGAGATAGCATTACACCTTGTTACATGTTTACATGGTTACATGTTTCATTCATGAGAACAAAAAATAAAGAAAAAACAATAGTATCTACAAAAAAAAAACAGTGCCTGCAAATTCAACTGTCCATATCAAACAAAATGATTGATTCCACAAGTTGTGCATGGAATACTTCTGAAGGTACTCCTTACTATATTCTTTATACAGGGAGGAATCACTTCATCTGATCTTGCTACAAAAGCTTTGGAAGCTCGGCGTGCCAAAGTAATGGGACAAGCTTTAGCTGGTGTGCCTTTGTGGCAGCTTGGCCCTGAGAGTAGACATCCTGGTGTCCCCTACATTGTTTTTCCTGGTAATAAACATATCTCATTTCCGTACCAACAGATAAATAACGTTATCTTTTTACTCAAGAAACTATCTTGCCACTCCCCCAATGACAGGTAATGTTGGTGATAACAGTGCTCTTGCAGAAGTGGTCCAGAATTGGGCTTGCCCTTCTAGAAGTTCAACAAAAGAACTTCTCCTTGTAAGTTCTTATCCACCGTCTACTTCTCATCAAGGTTATACATCGTTGTGTATTGCTTTAAATACTTAATCCGCTATTTTTTTTAATTGTGAAGAACGCGGAGAAGAGTGGCTATGCAGTTGGTGCTTTCAATGTATATAATCTTGAGGGAATTGAAGCTGTAATTGCAGCTGCTGAGGCTGAAGAAAGTCCAGCTATCCTGCAGGTTAGAATTCTTTGTTAGTATAGGATGTTTTCTTTGGCAGTTTATGGTGAAAATTATGTAATTTATCATGTTGGGTGTCCTCTAATACCACTCTTTACTGGAGTACGTTATGATAGGACTAGTAAGTACTCCCTCCGTCCCAAAATAAGTGTCTCAAACTTAGTATAACTTTGTACTAGAGTTAGTACAAAGTTGAGGCACTTATTTTGGGACGGAGGGAGTAGGTCGCAACAGCTAGCTCCAGAGGGATAGAAGCAATACCACACAGATCCTGAAATGGATTTCTACCCGTGTTTTTTCCGTAGAGAGAATTGCATTGATTCTCTCAACTCATGAGCTTTTCTCACTCTCAACACATGAGCTTTTCTCACTCTCAACTCATGAGCTTTTCTGCACCCATGCTCCCTGAACTCATTATCCATATTTTTTGTAGATGATATGCATCGTCTTGATTTCTAACCACGCTTATTGTGGTTGTTATTTAGGTTCATCCCAGTTCCCTGAAGCAAGGTGGAGTTCCGTTGGTAGCATGCTGCATTGCTGCAGCAGAACGAGCCAGCGTAAGACAAACAGAACCTATAATACTTAAAGAGTAAATGTGGACACCCAGTGATCTGGATAAAAATAGTTCATAAACTTTTATATAACTTGGGTTCCCACATATTACCTTCTTCTAGCCACCCTTGTTTTTTGAAACAGATAGCCACCCTTGTTTTTATGTTCTTTAAAGGTGATCATTCTGTGATTGTCACTCAGGTACCTATCACCGTCCATTATGATCACGGGGCTGACAAGCATGATTTGCTTGGAGCTCTTGAAATGGTAAGCGAAAAAATGTGTTGTTTTCTCAAATTTGCGGTACCAAAATGTTTTCCTAATCATGAATGATAAATGACCACATCTGAATCTTAATTCTCTGGACCATCTACTATGACCTTTTATTTTTCAGGGATTTGATTCAGTCATGGTGGATGGTTCCCATCTAACTTTAGAAGAGAACATCTTATACACAAAGAACATATCTTCCCTGGCTCATGCGAAAGGTATGCTTGTGGAAGCTGAACTTGGGAGACTATCAGGCACTGAAGATGGCCTGATAGTTGAAGAATATGAAGCGAGATTTACTGATATTGCTCAGGTTATTTTATCTTCCTGATAAATTTCAACTCTATGGCATTCTATCATTACAATCCTGTAATAATGCTTAACTTATATGTTACATATTTTCAGGCTGAGCAGTTTATTGATGAAACTGGTATTGATGCATTAGCAGTATGCATTGGGAATGTTCACGGAAAATATCCTCCCAGTGGACCGAACTTGAGACTTGACTTGCTAAAGGTTTGACAGAACAGCTATGAAAATCTTTGTTTGATCTTTCAGATTATGTGTGTGTTAACAGATCTTTTACCCTGTTTTCTGATTTTCTGATTTTTTTTTTCTAAATTTGTTAAGCTTTTTCCAAAAAAGTATGATCCTTCTCTGTCTTTTTTTATGTTTTACTTAATCTGTTAACATGACAAGGTGCTAATGCGTATATGTGATGCCATGTGTCTGCCATGAGTTGTTTGAGTCAGCTCAAACCGCTTGGAATAGGACCAAGGGGGTAAACCAGTAAAGCAAACATATTCAACAATTCAAGGTTAAAAACAGCTGGTTTTCTGGTTTTCGAGTTTAAGGTTGAAAAATGAACTTCTGCAATAGTTCAGGGTTGTTGGTAGGACTAGGGTTCCTACCGGGCCTTCGGCGTAGATTGTATGGGCAGGGAGGAGGGAGACGAGGGCTGGAGCGGGCACGCCGGCGATGAGGCGCCGTCGCGGCTAGGGTTTAGGTGCGGCGGCGGTTGGCTGGTGGCGGCTAGGGTTCCGGCTCCTCAGGGAGCCGGGCAACAGAAGATGATAATATCTTTATTGCTTGATCTCAAACGATGTCTTACAACTAGTATTTATAACTTTAGCCTAAGATAACTTGCCTAAAATAACTTGCCTAAGATAACCTGTGGGTCAAGCCCCTAATACTAATGCCCGGTGGAACTCTTCCTGGTATAAGACCAAACCGGTCATAACATCTCTCCCCGCGTGCGCAAACAGCTCGTCCTCGAGCTGAAAATCTGGATAGTGTTGGCGGAATTCTTCACGCTGCTCCCAAGTAGCCTCCTCCTCCGGAAGGCCTGTCCACTGAACGAGGATGAACCCGACGCCACGACGGAGCTGTGCCTACAACACCTTGGCCGGCTCTGGAAGAATGCGACCATCGGCGATGGGAGGAAGCGCCGGAGGAGCCGCCGGTGGCTCGCCACGGAAAGGCTTGAGCAGCCCCACATGGAAGACGTCGTGGATGCGAGCGCGTGCTGGAAGCTGAAGACGATAGGCCACCTTCCCAATGCGCTCCAAGATAGGGAAGGGCCCGGCGTAGCGAGGACCGAGCTTGCGCTTTGCTCGCGGGTCGAGTGACTGCGTAGAGCGGTGGAGAAGACGCAGCCACACCCAATCACCCACCGCGAACTCCGCCTCGCGATGATGATCGTCGTAGTAGTGCTTGGCCAGCTGCTGGGCCTGAAGGAGACGCTGACGAACCTCAGCAAGCATCTCGTCACGAGTGCGGATGAGTTCACCCGCAACCTCCGTCCGAGCTGTCTCCGGATCCACCGGAAGGATAGGTGGGGGTGGTCGACCATAGACCACCTCAAATGGCGTAGCGCGCAGGGCGGAGTGATAAGAGGTGTTGTAGCAGTACTCCGCCCAAGAAAGCCAGTCCACCCAAGCGCGAGGCCGATCACCTGTCACACAACGCAAATACATGGCAATGACCTTGTTAACCACCTCGGACTGTCCGTCCGTCTGAGGATGGAACGCCGTACTCAGGCGGAGCTGGACACCTGCCATCCTGAAGAGGTCGCGCCAGACATGGCCCGTGAAAACTGGGTCCCGATCACTGACGATCGAAGCAGGGAACCCGTGTAGACGGACGATGCCGTCGAAGAAGGCTCGGGCCACTGACGCCGCCGTGTATGGATGGCCGAGCGCGATGAAGTGGGCGTATTTGGAAAAGCGGTCGACCACCGTGAGGATGACCGACTTGCCACCCACCTTGGGAAGGCCCTCAATGAAGTCCATGGATATATCCGCCCAGACCTGAGATGGCACCTCCAAGGGCTGGAGTAACCCGGCCGGCCTTAGGGTCTCTGTCTTGTTGCGCTGGCATGTCTGACAAGACCGAACCCAGTCGCGGACCAGGGCGCGATCGCCAGGGATGTAGAAGTCGGCGCGAAGACGATGGAGGGTTTTCTGCATACCCTCATGACCCGCCGAGTGGGCGAGCTGTAACACCTGGTGACGGAGATCATCATGCGCCGGAACAAAGACCCGACGCCCATGTAGGAGTAGTCCGTCAGAGAAGCGCCAAGGCTCCTCCAGGTCGCCGGTCGTGAGCTGCTGTTGAAGGCGGACGGCGTCGTCGGCCGTCGCAGTGGCGCGGCGAATGTCGGCGAAGAGGCCGAACGTCGGCCCAGAGCGGATGCACAGGGCCGCCCCGGCGGACTCGTCGGCGGAGGAAGCGAGGTCGGAGTCGCGACGGGACAGCGCGTCGGCCATGGTGTTAAGGCGGCCTGGCCGATACTCGACGGAGAAGTCGAAGCCGAAGAGCTTGCTGATCCACTGGTGCTGCGGCACGGTCGACAGCCGTTGATCCAGCAAGAACTTCAGGCTGTAGTGGTCAGTGCGAATGAGGAAGGACCGGCCCCACAAGTACGGCCGCCAATGACGAACGACCTGTACAAGGCCAATGAGCTGCCGCTCGTATGCCGCCAGCTTAAGGTGACGCGGAGCGAAAGGCCGGCTGAAGAAGGCGAGGGGCCCATCGCCCTGATGAAGCACGGCGCCGAACCCTGCGCCCGAGGCGTCACAGTCCACCACGAACGGGCGGCCGAAGTCGGGCATCTGGAGGACGGGGCCCGTCGTGAGGGCCCCCTTGAGGGCCTCGAACGCCGTCGTCGCCTCATCGTCCCAGGCGAAAGCGTCGCGGCGAAGCAACCGTGTGAGGGGCGCCGCGATGAGCCCGAACTCCCGAATAAATTTTCGGTAGTAGCCGGCGAGGCCGAGGAACCCGCGGAGAGCCCGCGGCGAGTGAGGCGTCGGCCAGGCGGAGACGGCTGCCACCTTGTCGGCGTCCATAGCCACCCCGTCGGCGGAGATGACGGGGCCCAAGTATGCCACCGAAGGCGTCCCGAACGAGCACTTCGAGCGCTTAAGGTGGAGGTGGTGCGCTCGAAGCTCGTTGAAGACGATGGCGACGTGCTGGAGATGCTCGGCCCAAGACGCACTGTAGATTAGAATATCATCAAAGAAAACGAGCACAAACCGGCGTAAGTGGGGCCTGAGGATGTCGTTCATCAGAGCCTGGAAGGTCGCCGGGGCGTTGGTGAGGCCAAAGGGCATCACCAAGAACTCGAAGTGGCCGTGATGAGTCCGAAACGCCGTCTTGGCGATGTCATCCGGGTGCATGCGCACCTGGTGGTACCCCGACCGAAGGTCAAGCTTGGTGAAGAAGCGTGCCCCGTGTAGCTCATCGAGGAGCTCGTCGACCACCGGAATAGGGAACTTGTCCTTAAGTGTGATAGCATTAAGGGCACGGTAGTCGATGCAGAAACGCCATGTGCCGTCTGACTTGCGGACGAGAAGCACCGGCGCAGTGAACGGCGAAGTGGAGAGCCGGATGATGCCTGCCGCGAGCATGAGGGCACACTGCCGCTCCAACTCATCCTTCTGCAACTGCGGATAGCGGTAAGGGCGGACCGCCACCGGCGCGGAGCCTGGCAGAAGATGTATACGGTGATCATACGTGCGGGCGGGCGGCAGCCCCTGTGGCTCGGTGAAGATGTCATCATGCTGCTGTAGGAGACTCTCCAAAAGTGGGTGCTCGGCCGTGGCGGTGGTCGTGGCCAGTTGGAGGGGTGGTGTGGGTGCGGCACCCCCAATGCCATCCCACTGAATGCAGCGGACCAACCGCCAGAAGGTCATCGTCAGCGCATCGAAATCCCAGAGAATAGGACCAAGAGTGCGCAAGAAGTCAACGCCTAGGATGAAGTCGAAGCAGCCCAAGTCGATGCCGGCACAGGTGATGGAGAAGTGCTCGCCGCCGATAGTGATGGGCACGTCACGGGCGAGCCCGTGGCAGTGGAGACGGTCGCCATTAGCGACTGTGACTCGCAACTGCTCGCCGCCGGTGGGTTGTAATGCAAGGCGCCGCATGGTGGTCGCCGGCAGGAAGTTATGTGTGGATCCTGTGTCCACTAGGGCCACCAGGCGCTCGCCCTGGATCATGACCGGTAAGAGCATTATCCGTTCATCACGGATACCGGCCAGCGCGTGTAGGGACACCACGAGGGCGGTGGCCGGGGCGGGCGCGGGCGCCGCTGCGGTCTCCGCAAGGGCTGGGTCGCCGAGGCCATCCTCCAGTGTCTCCTTGCCCGTCTCGTCTGACGTCTCCAAGTAGAAGAGGCGGGGACAGACATGGCCAGGTGCATAGGGCGCATCACAGTTGAAGCACAAACCCAGGCGCCGGCGTTCCAGCTGTTCTGCCTGAGTGAGGCGCCTAAACGGCCGCGTCGGAGCCGGGGTGGCCGCCTGGGCGGCAGTGGACGGTGTCGGTGCTGCCGGAGACGGCCGAGTAGGCTATCGGCCCCCACGTGGCGCGGATGGACGCGTCAAGGCCTGGGCGCGTTGCTCGAATGCCCGGGCATAGTACATGGCCGTCTGAAGGTCTTGGGGCCCCTTCATCTCGACGTCCACGCGGATGTGGTCGGGAAGGCCACCGATGAAAAGCTCCGCCCGGTGTAAGGCCGTCACACCGGGCGCGTGGCACGCCAGGGCCTGGAAGCGGTCGGCGAAGTCTTGCACCGTGGAGGTGAACGGCAGGCGGCCCAACTCGGCCAGGCGGCTCCCGCGGACCGGTGGCCTGAAACGAAGAAGGCACAGCTCCCGGAAACGATCCCACGGGGGCATGCCGCCCTCGTCCTGCTCGAGGGCGTAGTACCATGTCTGGCGGCCCCGCGGAGATGGTAGGAAGCCAGCCAAGTGCGCTCCGACGCGGGCGTGCGCTGTCCGCGGAAGAACTGATCACACTGGTTGAGCCAGTTAAGCGGGTCGTCGGAGCCGTCGTATGTGGCGAAGTCGATCTTGGCGAGCCGGGATGGCTGCTGGTGTGGCACGCCCTGGCCCACTGCCTCGGCGGTGCGGAGGGCGGATGATGGTGCGCGGTCCATGGTGGAAGGCCCGGCGTAGTGCCCGGTGGCGCCCGACGCCTCGGGCTGCAACGGAAAACCGGACGGTGGACGAGCCTCCGGGTAGGCCGAGGGCGGCCCGTGGAGCCAGCCGGGGAGTGGCGATGGTGACGACGGGAAGCGGACCTCCTGGATCGGAAGCCTGCTCGGCGACGACCGGCCCAGGTTAGGGCTGGGCGGGGGCGGTGGAGGAACCTGCTCCGGGCCCGCTGGAAGGGATGGCGCGGCGGGAGACGGCGCGGCCGGCGCGGTCCAAGTGGGCCACTGCCGCCCGTGGGGGCGGCGAGTGGCGCGGTTGCCGTCGCAAGCTGCGGCGGCCATTGTGGCCAAAGCGGCGGTGCGGCCGCCTGGGCGGGGAGCGCCGGGTAGCCTCCGGTGATGGCCCCCGGTACCGAGTACCATGGCAGCGCCTGCTGACTCGTGGCCATGGCTGGATGGATTGGTGGCGGATGCCCTGGACCGCGGCGACGAGGTCGGTGAGGACGCCGGCCATTGCCTCCGGTGTGAACTGCTGCGTCTGCGGAGGAGGTGATGGCGGCGGCGTAGGTTGGATGGGGGCTGGTGGCCGCCCGTGGCCCGGCACGGTGCCGGGTGCCTGGAGGGGCGGCGCCGAGAGCGACGCCGTGATGCCCGCCAGGAGCGAGGCCGTGGCGCCCGGCGCAGAGGCGGCGGCGGTGGAGGAGTTGGCGGCAGCGGCGGTGGTGGAGTTGACGGGCAGCGGCGGTGGTGGTGGCGGTGGAGAAGACATGGTCGAAACCCGGGTATCTGATACCAAATTGGTAGGACTAGGGTTCCTACCGGGCCTTCGGCGTAGATTGTATGGGCAGGGAGGAGGGAGACGAGGGCTGGAGCGGGCGCGCCGGCGATGAGGCGCCGTCGCGGCTAGGGTTTAGGTGCGGCGGCGGTTGGCTGGTGGCGGCTAGGGTTCCGGCTCCTCAGGGAGCCGGGCAACAGAAGATGATAATATCTTTATTGCTTGATCTCAAACGATGCGGCGGCGGTTGGCTGGTGGCGGCTAGGGTTCCGGCTCCTCAGGGAGCCGGGCAACAGAAGATGATAATATCTTTATTGCTTGATCTCAAACGATGTCTTACAACTAGTATTTATAACTTTAGCCTAAGATAACTTGCCTAAAATAACTTGCCTAAGATAACTTGTGGGTCAAGCCCCTAATACTAATGCCCGGTGGAACTCTTCCTGGTATAGACCAAACCGGTCATAACAGTTGTAGAGTGATTTTTTTTTCCAACGGCTCGTGAATTATTTTGTAAAGGATTACCAAGCAAGTTTGTGCTTTTGCAAGCGACTTCTTTTGAGTAATGAACTCAGTATTTTTTTTCACTGAAACGATGACAGGAACTTCGTGCATTAACTATGAAGAAAGGAGTTAGCTTGGTACTTCATGGAGCATCTGGCCTCCCTCATGAACTTGTAAAGGTTTGATAAACTGTTTCCTTCTTTATTAAGATCAGCGAATTTGTACCTTGTTGCAGTAGTCTACCTCAGAGCGAACAATGAAAAAGGCATCGAGCGGGTGCAAGCTCGAGGAGCTAGGAAGGATGGAAGAAAGCTTGACTGTTTTTTCACTGAACTACTCGAACTTTTTGTTAGAAAAAGCGGAAATAGCTCAGTTCGAGAGAGGGTTGAGCTTCATCGGTTAGTGTGCACCAAAGGATGAGGTTTCTTTCCTGTCCTACAAATTGAAACCGTTTTAGCTTGATACTGCGATATTTGTTGAGTTTCCCCACACCAACTTATATCCTGAAAGGTTCAGGTTATAGGAGAATTGGGGAGCAATCGTTGCCCTCCATAAGACAATCATCAATCATGGTTGCACTGCATAAAATAATTCAACGACAAGGTTAAAAATGCTGGAGGGCTAGAGTTTCTGTGTCTATATCAGCTTCATAGTTTTGCAAAGAATCGGTAAAACGCTTCCTTTTGCGAAATAATTATACTAGAGCTTGTCAGTTGATGAAATTGGTCTTATTATTAACTACTGTCATATTTGAAATGTTGTTGTGCAAGTGATAATAACGTGTAACCAACTTGTGGAGCAGGAGTGCATAGATTTGGGGGCGAGGAAATTCAACGTGAATACGGAGGTCCGCAACAGCTACCTGCAGTCACTCAAGAAGCCAGAAAAAGACCTGGTTCAGGTCATGGAGTCTGCCAAGGAAGCAATGAAAGCTGTTGTAGCAGAGAAGATGCGCCTCTTCGGATCTGCAGGCAAAGCCTGAATGTAGCGACACAATGAAAAACTGAAGCCAGCAACACTCCACCATTAGGTATTGCATGGCCATTTTAGCGATCCTACTAGGCTTGTGACAGCGAACTGCCCGAACCAGTTTCTTAATAAAGCACTGATACAGTTGCGTGAAGAAATAAAATAACAAAAATTAGTAAATTGCAGTAACTTACCGGTACTCCCGTCCATGGTTGAGTGACTAGTTTTGCTTAATATAATATACTCCCTCCGTTCCTAAATCCGTTCCTAAATATAAGTCTTTTTTAGAGTTTCCAATAAGAGACACAATATGGGGCAAAATGAGTGAATCTACACTCTATGCTGCATCTATATACATCGTATGTAGTCCATAGTGAAATATGTAGAAAGACTTATATTTAGGAACGGTGGGAGTATAATGATGAGTTGTTATTTAGTCGTGATCTGTGACATGTGAAATTAAATTCCAAAACAGCTTCGTGAGAGAGGGTCAAGGGGGGGGGGGGGGGGGGGGGGGGGGGGGGGGGTCTGCAACCAATGATCTGGAGAAGATGGTGTGGTGCATGGTCCTCCCAGACGGCTCCAATCTTGACATCAGACTCAGATCTGGTGTGTATCGGCATGGGTGATTTGTCGTTTCTTTAGAAAGTCTTAGTGTCTGGTTTGGGGCGGTGAGACGGTGGCGCTTGTCTAGTGTAGGAATAATGTCCTGCCCACCTTTGTCCCGTTTCGTTGGTGTGCTTATTGCCGGTACAGGGTTGTGGAGTTGTGTTCCCGTCAGGTTTTCCGGGATCCAGTCGGTGTTGTTACCGGTGGATCCGTGTTTTTATTGTTCTCAAATTTTCTACAAGCCCTTTTCTGCATT

>TaFBA21

TCACTCTTCCTGACTTGCTCACTCCCCTGTCCGCTTTCGCTCTGCTCGCCTCTGCTTCGGGCTGGTCGTCGCCGTCGGCGAGCGAGATCACACGCCACTAGCGGGATGTCGCCGTCGTCCTCGGCGGCGAAGGTCGTCGCCTTCGTCGGCGCCGACGGGCTGAGCGCCGCGCTCGCCGCTTCTTTCACCCGCTCCGGCGCGATCGTCCGCTTCTACATCGACCCCAAGGTCGCCCTCGCTCGATCGCCCTTCCCTCGTTTCCCCGTTCCTTTCAACCCCGTTTTGGTTCTGCCTCCTCTCGGAGGTGTTTTCGCGGGATGCTCTGTCCTGACGGTGCCTGTCCTTGCAGGCGGATGGATCGGCGGCGACGGCGCTCGCGGAGCAGGGCGGCGGCGTCCGATGCGCCAGCCCCGCCGAAGCCACGCGAGGTGAGCGTATATAACGGTCTCCTGCACTGGACCGGTTCCAGATATGGACTTCTTGTGACGCGCGTGAATGGCATGCGTGCGTACGAGCCGTTTGTTGGAATGCCGAAGAGACCGTGCTCTTACGTGCTGAAGAATACTTCGCGCCGTGTGTGAGTTGCTTCTATCTGATCTATCAAAAACTCTTGGCGCCTGCAGATTCTGCGCTGGTCGTTGTGCTCAGTGACGCCGATGGTGTAGATGAGTTGTTCTTTGGAGCTCAGGGTATAGTGAAAGGTATGTACCGCGTGTGTGCTATTGTGTTGCCTGCCAGTCTGTCAATCTCATGCACCTGTTTCTGTACAAGTTATATATACACTTGACATTTGATATGATGTTTGCTTAAGCATTTCATAGGATTGTGCAAAGAGGCCATTGTATTAATTCGATCGACGTTGGTGCCTAGTCATCTTGAGAAGTTGGGCCAGAAGCTTGCAGGTATAATGCTAATGTGTGCATATATTGTCTTAGCTGGGAACAGTAGCTGCTGTGCTTGGAATTTTATGTGCCCTTTTAAGAATTAGAAAGCTGCACATTTAACAATTGTAAGGCCCTCTTTGGATGACTGGAATTAAAGAACATAGGAATAGGAATAAACACATGATTGGAATTTCATGTACTACTATAGTCTCCTACATGATTTTTTGTAGTGATGACCTTGGATGGTTCACAGGATGTCGGTGATGAATGACTGTGCTAGTTAATCTGGATTTTTTTTTTGCAGATGAGAAGAAGGGTATCTTCCTTCTTGATGCTTACATTTTTAGTGGTTTATCTGATGAACTGAAACAAAATATTGTCGTAAGTGATACACAGCTGAGAATCTGAACTGTGTGCTTGTATGCTTACGTTATTCTGTTATCACAATTTAGCTTTTGATTGTCAGGTTGTTGCATCTGGGAGAAAGGATGTAGCAGAAAGAGCTGGACAGTTCTTCTCCGGTAGTTCCAAATCTGTGTACCTTACTAATATTTTCTTCCTGGTCCTCATCTTATGATGCTGAAATTTTAAGCTATCTGACATTCCAGGTCTTGACAAAACCATTTACTTTGTCGAAGGTGAATTTGGCTGCAGCAGGTTGCATTCTTTCCTGAAAATAGCAAATATAAGCTAGTCGAGATATCAATTTTCACCCCTTCCATATATTCACCGTACTAAGTAGTGTAACTATTATTGTCACAATTTGAACTGTATGCCATTTATTTTTACAGCAAAATTAGGTTGGTTAATGATTTGCTGGAGAGCATTCATTTTGTTGCTTCTACTGAAGCAATGTTTATTGGTGTTCGAGCTGGGATTCATCCATCAATTATCTACGACATAATATCAAATGCTGCAGGAAGCTCAAGGTTACAAGGATTATTGAATTCTTTGTGTTCTGTCTGAACTCGTGATACTTGTAGGTTTATATTTCAACTTATGATCATACAATTTATTGACAGGATTTTTGTGGAGGTGGTCCCGAAAATTTTGAGTGAAGATCCATTGCTTATTGATTTTCTGAAGTCACTAAAGAAACATGCAGTAAGTTCCTTTGCTTCTCCTTTGGGGCGTGGTATGGGATTCTTAGGATACTTTACGCAATAAATAAACAGTGCAGATAATTAAGTATGTCCCTTTTCTCCCTTTTATGAAAGAGGGGATTGCACAAACTAGAATGTTATTTTAAGTAAAAGATATAAATACGTAGATGATGTGCACATTTATTGCTACGGAGTTTGTATGGACAATGAACGAAGGCATTCTATGCGTATTGGAGCCATGACCGGTGGTAAGAGCAGGGTAGCTGGGATGTGATATTGAAAATGGAGGGTCCGGGACTAGCAATTGGAAGAGAGGAGATTTTTTGTTTGATCAAGGTATTCAATCCTCTTCCCAAAAATAGGGTAGGTACTGGACAGGAGATTAGATATACATGAGACTGTTGCCGTGGCAAAGGTGGCAGGGTGCTGAAAAACCAGGGGAGGGGAAATGGCAGGGACGAAGCTAGGAAAAAAGTTTGTTGGGGTCATGTCTATGGCAGTGGGGTCATCTTCAATAAATAGATATTGTTTAGTGCTAAATTAATGAAGGATTTCCTAATTTCATTGGGGTCAATTGACACTATGCCTACAAGGCAGCTTCGTCCCTGGGAAATGGTGAGAAGTGATAAATAGTAGGAGTATCACATTGTACCTTGATAGATAGCAATCAAGTTACTTAAGAAAGCTAGCTAATCCCATGGACTTACTCATTTAGCAGCATATTTGAGATATTTCAGTGAAATTTTTGCACACTTTCTTTTAACTATAGTTTGCATTTCAGTTTTGTGTCCAGTTCCATGCTCTTAATCTTGTAAGTCAACCCCATTTTTGAGAGAGGATTAAATGGTTAATTAGTAATGTGCCACTCTGATCTTACAAACTGTAAAGGAATATTGCTACTGCATGGCTGATTGCCTGAATGCTTCTGTTTATTAAAACAAAGTTTTTGCTCTTAAACAATATAGAGCTACGTGATGGATACGGCTAAAGCAGCAACGTTCCCTCTGCCACTTCTAGCTGTTGCCTATCAACAATTAATACATGGTACGATGTTAAAGCTTCTTTACTTCCAATACATCTTGAAATTCTCCTGATTGCCCGAACATGCTAGTACCTTCTTATTCATGCACATTTTTTGTACTTATCTTCAGGTTCTTCAGGAGTAATTAGAGATGAATCCGCTTCGCCGTTGAAGGTGTGGAATAACTGTCAGCTTTCTCAAGATGCTTCAATTTATCAAAATTATCTTAATTTAAATCATCATTTTGCAGGTTTGGGAACAACTATTTGGAGTAAACATCGTAGATGCTGCCAGTCAACAAATCTATGACGCAAGCAAATTAGCTGACCAGCTTGTTATGGCCTCGAAAGCAGCAAAGAGGATCGGCTTTATTGGTCTTGGAGCTATGGGCTTTGGAATGGCATCTCATTTGTTAAAATCAGGGTTCTCCATAACTGCTTATGATGTAGGAACTCTTCTGCTCTTGTTCTTCCCAACTGCATAATTGACCGTTTGAATTGCTTCAATTCAAAATACAAAGAATGAAAGGGTCTTCACTTGGTTTCTTATGCATTAGTGCTTCTGTACATATGTTGCATTTCAAGTACCCTGAACAATCTTATGGATCATAGATTTGACATCTGTGGTATTAAGCTTATAATTTTGCTAGATGGAACCGTGGATGGCTTAGATTAATCTTAAGGTATCAGAAAGGGCAGCAGTAATTCGAATAATAACGAATAGATCACAAACTCTTCTCTTTTCAGTAGTCCATTGGGCGCTCTGAATTTACACAAATATACAGTTGAGGAAATAGGATACTGTTCACCCTTTTCAACTTACATATCTTTTGTTTGTAAATGATGTTAGAGACCTATAGTGTTATGCATGTTCTTTTGAAAAAAAAAAAGTTCTTCGGCTCCACACGTGAATAATTTTATACCCTTTACCCATTAGGTCTACAAGCCAACACTGGCCAGGTTTGCTGCTTTAGGTGGATTGACTAAAGATTCTCCTGAGGAAGTATCAAGAGGTACGTTGCAACCTTCTAGTATAAAGTATAATTCTCTCTTCTGAAGTTTACATTTGTTACCTATAACAGTATAATCAGTATTATATGATTTTCTGACTGAATGAAATTAGTTCAGACCTCATTATTCTCTTGTATTTATCTTATTCTTTATTTTTGTGCCATTCATCGAGTCAAAAGGTAAATGACATGTGGGCCCAGCCCCACCTCATCACTACTTAACAGCAATAGCATGGAATGGAAATACTGTTTATGATGTATCAACCGTGATACCGTGTCTGAAGTATTAACCATAAATTGGTTTCAGATGCGGAAATCCTTATCATCATGGTTGCAAATGAAGTTCAAGCTGAAAGTGTCCTATATGGAAATGCTGGTGCTGTGTCAGGTAACATATAGACCCCCCCCCTTTCCTGGCTCTGCTGATTATTAGCATCTCCCAGGTAGGTTAACTAATTTTTGAGCTGTACGTACATGGTTGTGAATATCCTTTGTCTTTCACTTTCAGGTTTGCCAGCAGGAACATCCATCATTCTGTCATCTACAGTTTCTCCTGGCTTTGTGACTCAACTCAAAGGAAGATTGGAAGGTAGCCGCTCTTTTTTATCTCTCCTGGGCATGTTTGCATTAAAAATAAGAAAGAATACCACAAACATTACTGGTCAACAAAAACCACATTGTGTAACTCATAAATCCAACTCATGAAATCTAAAATCTTCTGTTTGTGATTCTGGGTGAGAGTTGAGTCCTGTAGCTTCAGTGTCTCACAAGTAATGTTCGTTTACCTTTTCTCATTATAAAACACCAGATTTATAATCATTTGCAGAAGAAGTTGCAATGTCTGCTGTGACAGCTAGCCTGTATTTTGTGTTGGGATGTTGTTATGAAGTTGAAGTTGCATTTTAAGGGAAATGTTCCGGCAGAAGGCCTTTGGGGTGTTACCCAGGTTCCAAAAGTCACAGAAACATCCAAAAAGTAGTACAATGCTAACAGAAGATGAATCTATTACTAAACAAATCAGATCCATATTTGACCTATGATCAAGAAAGCTGTGATAGAAGCTTCAATCGTTTTCAGTTATTGTCGAAAGGAATCTGAATTTTGTTTTCTCCATGTGTAGGTTAAATCCGTATCTCGTTAGATTTATGTCGTGTAAATTGCGTCAAGAGCACTTCTTTTTGGTCACTTTTTATATTGTTTCCCGTAATACACCAAGGCCTTGCAGTACTAGAACCATTCTCTACATTTTAAATATGTCGGCAATGTTGTTTACTGCTTTCACTGATGAATCTTTTGGAATCTTTAACTACTTCTATACAGCTGAATGCCGAGAGATAAAGTTGGTTGATGCTCCGGTATCTGGTGGTGTCAAGAGAGCAGCGGACGGGACACTAACTGTATGATTAGCGAAACATTAATCTAGTAGAAGTGATGATGTAACCTTTATGGATATAAATATTTGCACTTTTTTTGACAGGTAATTGTTTCGGGAACAGATGAAGCTCTGCATTGTACGGGTAGAGTTTTGTCAGGTATGAAAAAGGTTATCTGAACACCATCATATGCAGTATACAAAGTGCGGAAACTGGTCGATAACTATTATCTTATTTTTGCGTAACAGCACTAAGTGAGAAACTATATCTCATCAAAGGAGGATGCGGAGCTGCGAGGTGATACACACATTTTGTAGTGATATACCTTCCTTACAGGGTCTTATTTCTGCATAATGGCTCTTTTTTCATTAGTGCTGGCGCTTGTGAATCAACATCTCTCTTCTGCCATTTTTCCCAGTTCTGTTAAAATGGTGAACCAACTTCTTGCTGGGGTCCATATAGCCTCAGCAGCTGAAGCAATGGCGTTTGGTGCTCGATTAAATTTGCGAACAAGAAGAGTTTTCGAAATTATTCAACATGCAAGAGGTTACTCGTGGTATGTGTCATTGTTTAATCCACTCTAATGAATCTTCCTTTTTTACTCTTCACTGTTTAGACTTGCTAATATTGGCCGAGTAATAAAATGATTATGAGAAAGAAACGAAACAATTAGTTCTACTTGTTTTTGCTATCAGCTCCCTGTAGGTCAGTCTTTCAGATATCTAGTGGTGTGTTCTAGCATGAGTGAAGGTGGCTATCTGATGATTGATACACTCAATGTTCATATTGCAGGATGTTTGGAAACCGTGTGCCACACATGCTTGATAATGATTATACACCTTTATCTGCGGTGGACATATTCGTCAAGGATCTTGTATGGATAACCGAGCTATTAACTTATTTGTTTTATGAACTATTTTCTCTGTTCGTAAATGTAAGACGCTTTTGCAGTTCAATTCAATTTGAACTGCAAAAGCGTCTTACATTTAGGAACAGAGGGAGTACTAAAAAAGATAATAATGCATTTATCTCGTCTTGAACTGCAAAAACCTCTTACATTTGGGAACAGAGGGAGTACTAACAAAAGATAATAATGCATTTATCTCGTCTATTTCAGGGTATAGTATCTCGTGAAAGCTCCAACTTGAGGATCCCATTGCATGTCTCTAGTGTTGCTCATCAGCTGTTTGTTTCAGGTAATTGTCTGTGATAAATCAAAGATCATTTTCCATTCTATGTTTTGGTTCCAATGTTCATCAAGTCATGCTATATTTCTTATCTTCAATGGAAGTAGTTACCCAAATTAACAGAACGTTGCATGGCCACCCCCTCTGTTTTCTATACTAGTAGTATATTACAGTTAGTTTTAGAAGAAAACAAATCAGCTTACAGTTATTGTTTGGATAACAGTCTTAAAATAGGATGGCTATTGTTTTATTCATTTGATGTATTCCAACAGGCTCTGCTTCTGGATGGGGTCGATATGATGATAGTGCTGTTGTAAAGGTGAGCAGCTCACAGCTCTCAGTAGACAGAACGTATGCCAAAATATGATACTCCCTCTGTTCATTAATATAAGACGTTTTGGCAGTTTAATTTGAACTGCCAAAACATCTTATATTAATGAACAGAGGTAGTATACTGATAGCCTTAATTTAATTCCCTTTCCTAAACGTGCCCGCTGATTGCGCCTGGCACAGGTATATGAGACATTATCTGGAGTAAAAGTAGAAGGAAGACCACCCATGCTTAATAAAGAAGATGTTCTGCGTTCTCTTCCTGTTGAGTGGCCAGAAGTTCCAATGGATGATCTTGTTTCCTCTGCATCTCATGATAGCAAAAAGGTTCTTGTAGTTTTAGATGATGATCCAACTGGAACTCAAACAGTTCACGATATAGAGGTCTTAACTGAATGGTAAATGAAAGCATATTGTCTTTTAGATTACATATGTTTTCTGCTAGCATTTTCGTTTTGCCCATGTTCCGTACATAGTTCCATAAGCTTTGCTTGTAATTCTTAACTGCAGGCCAGTTGAAGCCCTCACAGAGCAGTTTCTGAAACTACCAACCTGCTTTTTTATTTTGACAAACTCCCGTTCAATGATTGCTAATAAGGTAGGAAAGTGCGGTCTTAAACAGTTTATCTTTAGGGTCTTTAAATATATATTTAAAGCCTTCTCACTTTCATAGGCCGCACTGTTAGTAAAAGATATCTGCAGAAATCTTGAAGCTGCAGCAAAGACTGTCCCTGGTATTAGTTATACAGTGGTCTTGCGAGGTGATTCCACTCTACGTGGCCATTTCCCAGAGGTTAATATCTGAAACTCTCTCTGTTTTTCTGTTCCATTTCATTTCTCTGATCATATCATACTCTTCTACTTTCCAGGAAGCTGATGCAGTTGTTTCAGTATTAGGTGACATGGATGCTTGGATAATATGCCCATTCTTCCTTCAAGGTGGGCGGTACACTATTGATGATATTCATTATGTTGCAGACTCTGAAAGGTAGTCTGTGCACGATCACTATGCAATGTATTTAAATATGAAAATAACACATAACAATATTGATGTCCAACATTCTCTTAACAGATTGATTCCTGCTGGTGAGACTGAGTTTGCCAAGGATGCAGCTTTTGGCTATACATCTTCCAATCTCAAACAGGTACTGTTGTGGAATTGTTTGATTGGTAAAGCGAAGAGCGAAGAGCAAGACCTTAGTAACTTGCTTTGTCCTTGAATTTACAGTGGGTTGAAGAAAAGACTAAAGGAAGGATTTTGGAGAACCAAGTTTCAACAATTTCCATAAGTCTTTTACGTAAAGAAGGACCAGATGCAGTCTGTCAGCTTCTCTGCAGCTTGGAGAAGGTATATGATAGTAAACATGGAAAATATGATTGGCGGAGCTTGGAACAAAATGAAGGAGAGACCAACAATTATTGTTAGATGAATTAAGATGTATTTTTGTTGTTTAATTAGCAAAGAAGCTTCACCTATTTAAACAAAGTAGTTACAAGATTAAGGGCAGGCCAAGGCGCACCTTAGTCACCACTATGCCCCTCCAGTGCATATGAACAGTTATTTTGAGTCAGTTTAATTTTTTTAGGATATAATTATGAATTATTAGTTTTGTAGATATTTCATATTCCCATTTCTGTATGCACTCATGGCAGCTATTTACTTGAATGGCTAATAAATGTTGCAGGTGTTTTAGGGACTCTAGAACTGTACTGGCTGATTCAACCATATAGTATCATAAATCCTGTTTTGATTCCCTCTCTTCTTGATTTACCTTGAGAATTTCCTGACAGGCATTTTGACTCTTTCTAGGGCTCTGTGTGTATTGTCAACGCTGCCAGTGAAAGGGACATGAATGTCTTTGCTGCTGGAATGATCCAGGTATGTTATGCCACTCCATTTCTACGAGAAGCATTTGTTCTTAGTGTTAAGCTTCATGCACTAGCCAACGCAACCAAAAGTCCGAACTGATGGAAAGGACTAGCAATCCACATATACACTTCAACACCCCTTATCACGTGTGACGCGGAAAGTCAACACGTGGATAGACTTAGAGGTATGGCTCAAGAGGCCTATACGTGGACACAAAGGGGAGAACAACAATTTTTAGATTAATTGTGTAAGCCAGGACTTAAACTCAAGACCTTAGGCTCTGATACCATGTTAAGCTTCATGCACTAGTCAACGCAACCAAAAGTGCCAACTGATGGAAAGGGCTAGGCAATCCACATATACACTTAAACACTTGGTACCTATTCCTTTCGAGTTGATACGATAATGCGTCTTATTCTAGTATTGATGCTCTGTTTTGATCTCCTATTCTTCCTTTTCTTTTTACGTTAACTCAACAGGCTGAATTGCAAGGAAAGCGGTTTCTGTGCCGCACAGCTGCAAGTTTTGTGAGTGCTAGAATTGGAATCAAGCCAAAGCCACCTATCCGCCCAAATGACCTTGGATTAAAAAGAAATTTAGCGGGAGGCCTCATAGTTGTTGGTTCTTATGTGCCAAAAACTACGAAGCAGGTTAGCCTTGTGATGCTTTAAGATTTTGTCAGTCATATTTATCTCATTCGCATGTTCTTTTGCATTTGTTAGGTCGACAAGCTTCGTTCACAATGCGCACAATCCCTCAGAGTGATAGAGGTAATTTATATCTTGGCTGTCATGCCTAAACATGAACCATCAACCATCGTATACATCTACTTGGATAGAGTTTGTGGATAGTACTATCTTAAGTCGGATGGTTTTGCTTGAGATCAATTTGTGGTGTTAGTATTGTGTTCTCTTTTTACTCACTGTAGGTATCCGTTGAGATGATCTCACTGAAATCAACTGAGGAGAGAGACCAAGAAATCAGCAGAATTGTTGAATTGGGAAATGCTTATATACAGTCCGGGAGAGACACGCTAGTCGTCACCAGCCGCCAACTCATTACCGGAAAAAGTTAGCATCTTTCTGATTATCATATGGCTAAATGTGTTGTTTGTTCCATTAGCAAATAAAGTGATCAAATGCTGAGTCAATTTTGATTTCTAGTCTCTTAATGCCAACAATTCCTAAGCCACAACCTACCTGCCGAAGTGTTGAGCAAGTTGTGCTGGTAAAAACTGTTTACTAACAAATGATGTGGAGATCCTCAGGGCTAGCACACAATTGCCCCCAAACTCCACACAGTATCAGAGGCTCTCAGCCTAATTCTATTCCCTGAGCTCAACTCAGATCACTTCTGGGGAGATAGTTTGTTTACTTTTATCATATTCTAGGCATGAACCGGGAGTCTGGGGCTAACTGTATTGGAAGACAGGTCAAACTGTCAAAACTTTTTAAAAGGGAGGAATTGATAAAATAAAGTTGCTTATTGTTGTCAAAGTCATGCCCAAAAACGCATTAGCCACCAGTAGAGCCATTGAATGAATCTCTACATTCAACCACAACCACAATCACAACGTGAGTGCATTGTCCCAATCATTTGAATAGAACATCTCCAGTTTGCATCATAATCTTTTCTAGTGACCAAAACCAAACTAACTCCAGTATACGTTCCTGAAATTGTGTGCTAGAGGCAGATACCTAACTCAGCTTTTCAAAAGAGTATGTTCAAGAATAGCCGCTGTTATCTTCATGTAAGCATTAGCAGTACAAGATAACTGACCTATTAGTATAGTTTGCTACCTTTTGTGTGCCAAACGAAAGAAGATAATTTCTTTCTTTCATATCTGTCCTGAAGTAGTTTGACCACCTAACAAGATTGTACCTTTCATGTTGCAGCTCCTGAAGAAAGCTTAGAAATTAATTACAAAGTGAGCTCAGCACTAGTAGAGATTGTGCGAAGAATTGATAGTCGACCCCGTTACATCCTTGCAAAGGTAAAATTACACAGCCGATTTCTTCTTTCTGAACAAATTGAAATTTAGTTGTTACTTGGTTTATGGAAATTTGCCAATCTCTGGAGCTGTTACTGTTAGGTCAAAATAGTTGTTCTGTTTTATTGGACCTATGCCATTAAATACTTAATGAAATATTGAGATAGCATTACACCTACATGGTTACGTGTTTCATTCATCAGAACAAAAAAGAAGAAGAAAAAAGAATAGCATCTACACACACACACAAAAAAAACAGTGCCTGCAAATTCAACTGGCCATATCAAACAAAATGATTGATTCCACAAGTTGTGCATGGAATACTTCTGGAGGTACTCCTTACTATATTCTTTATACAGGGAGGAATCACTTCATCTGATCTTGCTACAAAAGCTTTGGAAGCTCGGCGTGCCAAAGTAATGGGACAAGCTTTAGCTGGTGTGCCTTTGTGGCAGCTTGGCCCTGAGAGTAGACATCCTGGTGTCCCCTACATTGTTTTTCCTGGTAATAAACGTATCTCATTTCTGTACCAACAGATAAATAACGTTACCCTTCTACTCAAGAAACTATCTTGCCACTCCCCAAATGACAGGTAATGTTGGTGATAACAGTGCTCTTGCAGAAGTGGTCCAGAATTGGGCTTGCCCTTCTAGAAGTTCAACAAAAGAACTTCTCCTTGTAAGTTCTTATCCACCGTCTACCTCTCATCAAGGTTATACATCGTTTTGTATTGCTTTAAATACTTAATCAGCTATTTTTTTTCTTATTGTGAAGAACGCGGAGAAGAGTGGCTATGCAGTTGGTGCTTTCAATGTATATAATCTTGAGGGAATTGAAGCTGTAATTGCAGCTGCTGAGGCTGAAGAAAGTCCTGCTATCCTGCAGGTTAGGAATTCTTTGTTAGCATAGGATGTTTTCTTTGGCAATTTATGGTGAAAATTATGTAATTTATCATGTTGGGTGTCCTCTGATACCACTCTTTACTGGAGTACGTTATGATAGGACTACTAAGTAGGTCGCAACAACTAGCTCCAGAGGGATAGAAGCAATACCACACAGATCCTGAAATGGATTTCTACCCGTGTTTTTTCCATAGAGAGAATTGCATTGGTTCTCTCAACTCATGAGCTTTTCTCACTCTCAACACATGAGCTTTTCTCACTCTCAACTCATGAGCTTTTCTGCACCCATGCTCCCTGAACTCATTATCCATATTTTTTGTAGATGATATGTATCGTCTTGATTTCTAACCACGCTTAATGTGGTTGTTATTTAGGTTCATCCCAGTTCCCTGAAGCAAGGTGGAGTTCCGTTGGTAGCATGCTGCATTGCTGCAGCAGAACGAGCCAACGTAAGACAAACAGAACTTAGCATGCTTAAAGAGTAAATGTGGATACTCAGTGATCTGGATAAAAATAGTTCGTAATTTTTTATATAACTTGGGTTCCCACATATTACCTTCTAGCCATCCTTGTTTTTTGAAACAGATAGCCATCCTTGTTTTATGTTCTTTAAAGGTGATCATTCTGTGATTGTCATTCAGGTACCTATCACCGTCCATTATGATCACGGGGCTGACAAGCATGATTTGCTTGGGGCTCTTGAAATGGTAAGCGAAAAAATGTGTTGTTTTCTCAAATTTGCAGTACCAAAATGTTTTCCTGATCATGAATGATAATGACTACATCTGAATCTTAATTCTCTGGACCATCTACTATGACCTTTTATTTTTCAGGGATTTGATTCAGTCATGGTGGATGGTTCCCATCTAACTTTAGAAGAGAACATCTTATACACAAAGAACATATCTTCCCTGGCTCATGCTGAAGGCATGCTTGTGGAAGCTGAACTTGGGAGACTATCAGGCACTGAAGATGGCCTGACAGTTGAAGAATATGAAGCGAGATTTACTGATATTGCTCAGGTTATTTTATCTTCCCAACAAATTTCAACTCTATGGCATTCTATCACTACAATCCTGTAATAATGCTTAACTTATATGTTACATATTTTCAGGCTGAGCAGTTTATTGATGAAACTGGTATTGATGCATTAGCAGTATGCATTGGGAATGTTCATGGAAAATATCCTCCCAGTGGACCAAACCTGAGACTTGATTTGCTAAAGGTTTGACAGAACAGCTATGAAAATCTTTGTTTGATCTTTCAGATTATGTGTGTGTTACCAGATCTTTAACCCTGTTTTCGGATTTTCCGATTTTTCTTAAATTGTTAAGATTTCTCCAAAAAAGTATGATCCTTCTCTGATTTTGTGTGTGTTTTACTTAATCTGGTAACATGACAAGGTGCTAATGCGTATATGTGATGCTATCTGTCTTCCATGTGTTGTTTGAGTCAGCTTGAACCGCTTGGAATAGAACCAAGGGGGTAAACCAGTAAACATATTCAACAGTTCAAGGTTTAAGACAACTGGTTTTCTCGTTTTTGAGTTTAAGATTGAAAAATGAACTTCTGCAATGGTTCAGGGTTGTAAAGTGTTTTTTTTCCAACGGCTTGTGAAGTACAGTATTCTGTAAAGAATTACCATGCAAATTTGTGCTTTTGCAAGCGACTTCTTTTGAGTAATGAACTCGGTATTTTTTCCCATTGAAATGATGACAGGAACTTCGCGCATTAACTATGAAGAAAGGAGTTAGCTTGGTACTTCATGGAGCATCTGGCCTCCCCCATGAACTTGTAAAGGTTTGATAAACTGTTTCCTTCTGTATTAAGATCAGTGAATCTATACCTTGCAGTAGTTCTTGTTTAGTTTCCCGCACCAACTTATATCCTGAAAGGTTCAAGTTATAGGAGAATCGGGGGGCAATCGTTGCCCTCTATAAGACAATCATCAATCATGGTTGCAGTGCATAAAATAATTCATCGACAAGGTTAAAAATGCTGGAGGGCTAGAGTTTCTGTGTCTATACCAGCTTCATAGTTTTGCAAAGAATCAGTAAAACGCTTCCTTTTTGTGAAATAATTATACTAGAGCTTATGAGTTGATGAAACCGCTCTTATTATCAACTACTGTCATATTTGAAATGCTGTTGTGCAAGTGTTAATAACGTGTAACCAACATGTGGAGCAGGAATGCATAGATTTGGGGGTGAGGAAATTCAACGTGAACACGGAGGTCCGCAACAGCTACCTGCAGTCACTCAAGAAGCCAGAAAAAGACCTGGTTCAGGTCATGGCGTCCGCCAAGGAAGCAATGAAAGCTGTTGTAGCAGAGAAGATGCGCCTCTTCGGATCCGCAGGCAAAGCCTGAATGTAGCGATACAATGAAAACTGGAGCCAGCAACACTCCACCATTAGGGGTGTTGCATGGCCATTTTAGCGATCCTGCTAGGCTTGTGACAGCGAATTGCCCGAACCAGTTTCGTAATAAAACACTGATACAGTTGCGTGAAGAAATAAAGTAACAAAAAATAGTAAATCGCAGCAACTTACCGGGGCTCCAGTTCATGGTTGAGTGATTAGTTTTGCCTTTGATGTATAATGAGGAGTTGTTATTTAGTCGTAATTTGTGACATGTGAAATAAAATTATTGAACTAGAGAGGGTCAAGCGGGCGTCTGAAACCCTAGCCGTG
